# Transcriptomic Analysis of Drought Stress Responses in *Ammopiptanthus mongolicus* Leaves Using the RNA-Seq Technique

**DOI:** 10.1371/journal.pone.0124382

**Published:** 2015-04-29

**Authors:** Fei Gao, Jianyue Wang, Shanjun Wei, Zhanglei Li, Ning Wang, Huayun Li, Jinchao Feng, Hongjie Li, Yijun Zhou, Feixiong Zhang

**Affiliations:** 1 College of Life and Environmental Sciences, Minzu University of China, Beijing 100081, China; 2 College of Life Science, Capital Normal University, Beijing 100048, China; 3 The National Key Facility for Crop Gene Resources and Genetic Improvement (NFCRI), Institute of Crop Science, Chinese Academy of Agricultural Sciences, Beijing 100081, China; Institute of Crop Sciences, CHINA

## Abstract

*Ammopiptanthus mongolicus* (Maxim. Ex Kom.) Cheng f., a relic tree of the Tertiary period, plays a critical role in maintaining desert ecosystems in the Mid-Asia region. Genome-scale gene expression profiling studies will provide deep insight into the molecular mechanism underlying the drought tolerance of *A*. *mongolicus*. In the present study, we investigated the transcriptional changes induced by drought treatment in *A*. *mongolicus* leaves by establishing a comprehensive transcriptome database and then performing a Digital Gene Expression (DGE) analysis using Solexa sequencing technology. A comprehensive transcriptome database was obtained by assembling the Illumina unigenes with expressed sequence tags (EST) available publicly, and other high throughput sequencing data. To analyze the dynamic and complicated gene regulation network during PEG6000-induced drought treatment in leaves of *A*. *mongolicus*, a time-course gene expression analysis was performed using tag-based DGE technology, which identified 437, 1,247 and 802 differentially expressed transcripts in 1, 24 and 72 h drought stress libraries, respectively. GO and KEGG analyses revealed hormone signal transduction and phenylpropanoid biosynthesis were enriched during drought treatment. A batch of drought-regulated transcription factor transcripts were identified, including the subsets of HD-ZIP, bZIP, WRKY, AP2/ERF and bHLH family members, which may play roles in drought response in *A*. *mongolicus*. The sequence collection assembled in the present study represents one of the most comprehensive transcriptome databases for *A*. *mongolicus* currently. The differentially expressed transcripts identified in our study provide a good start for identifying the key genes in stress response and performing functional analysis to reveal their roles in stress adaptation in planta.

## Introduction

Forest ecosystems have been transformed, to a large extent, by global climate change. A typical example is the evolution process of dry lands in the mid-Asia region, including northern China and the Republic of Mongolia. Fossil evidence showed that the vegetation in this area had been dominated by evergreen broadleaf forest in early Holocene and middle Holocene. As the climate became colder and drier in late Holocene, which was partly caused by change in the intensity of the Asian summer monsoon, the forest was gradually replaced by temperate steppe and then by desert [1,2]. Now, almost all evergreen broadleaf trees in mid-Asia have vanished. However, two evergreen broadleaf plant species, *Ammopiptanthus mongolicus* (Maxim. ex Kom.) Cheng f. and *A*. *nanus* (M. Pop.) Cheng f., the only two species in genus *Ammopiptanthus* (Leguminosae), survive the dramatic climate change. Thus, *Ammopiptanthus* is believed to play an essential role in maintaining desert ecosystems in the Mid-Asia region [3]. Genome level studies on plants in genus *Ammopiptanthus* will promote our understanding on how the plant responds to climate change.

In China, *A*. *mongolicus* mainly distributes in the desert and arid regions of Inner Mongolia and Ningxia Autonomous Regions, as well as Gansu province. The tree species is a relic of the Tertiary period and can grow in the environment with multiple stresses. Previous studies revealed that *A*. *mongolicus* showed high levels of tolerance to drought, high salinity, high temperature, cold, and freezing stresses [4,5]. Several genes presumed to play important roles in stress tolerance of *A*. *mongolicus* were cloned and characterized, including *AmNHX2* [6], *AmLEA* [7], *AmVP1* [8], *AmCBL1* [9], *AmCIP* [10], *AmGS* [11], and *AmHDG1* [12]. These studies have provided important data for understanding how individual genes are involved in the stress response in *A*. *mongolicus*. However, systematic investigation into the gene regulation network under stress conditions, using whole genome gene expression profiling methods, is needed. The efforts had been hindered by the limited nucleotide sequences available in the public database before 2012. Recently, two studies had been carried out using next generation technology (NGS) to establish a large-scale transcriptome database from *A*. *mongolicus* tissues and to screen for the stress responsive genes [13,14]. These studies validated the effectiveness of NGS in studying stress response mechanisms in a non-model plant like *A*. *mongolicus*; however, tissue-specific whole-genome gene expression profiling is needed to further elucidate the complicated gene regulation network in *A*. *mongolicus* under stress condition.

Drought is one of the most severe environmental constraints to plant growth and productivity. A number of studies had been performed to investigate drought responses in plants using transcriptomic and proteomic approaches [15,16]. A batch of stress related genes were identified and further analyzed functionally via transgenic studies [17,18]. These studies made substantial contributions to our understanding on plant drought tolerance mechanisms. However, most genome-scale studies were conducted in model plants like *Arabidopsis* and rice, and far less research has focused on non-model plants. This is partly due to the deficiency of nucleotide data of non-model plants and the newly emerging NGS technology will probably change the situation [19]. At the same time, among the small amount of drought tolerance studies on non-model plant, most works were conducted in herbaceous plant, and less attention was paid to woody plant, such as plants in the genus *Ammopiptanthus*.

In the present study, we established a comprehensive transcriptome database by sequencing and assembling the leaf transcriptome of *A*. *mongolicus* and then assembling the leaf transcriptome sequences with other EST sequences. We conducted time-course gene expression profiling in *A*. *mongolicus* leaves in response to drought treatment, so as to investigate metabolic adjustment and identify the genes that may play a vital role in drought adaptation in *A*. *mongolicus*. The present work provides important data for understanding the drought tolerance mechanism of this desert plant and establishes an important transcriptomic database for further study.

## Results

### Sequencing and assembly of *A*. *mongolicus* transcriptome

A cDNA library from leaves of *A*. *mongolicus* seedlings was constructed and sequenced using the Illumina HiSeq 2000 platform. In total, around 68 million paired-end 90 bp reads were generated. All read data were deposited in the National Center for Biotechnology Information (NCBI) and can be accessed in the Short Read Archive (SRA) under the accession number SRX381742. After trimming adapters and filtering out low quality reads, more than 62 million reads were obtained, with the total nucleotides of 5.4 G, approximately 7-fold coverage of the estimated genome size of *A*. *mongolicus* [14]. Using Trinity [20], these reads were further assembled into 154,309 contigs (Table 1). The mean contig size was 304 bp with a length range of 90 to 4,823 bp. Using paired-end joining and gap-filling, these contigs were further assembled into 73,725 unigenes with a mean size of 697 bp, including 10,560 unigenes with a length ≥1,000 bp (Table 1). The size distribution of these contigs and unigenes is shown in S1 Fig.

**Table 1:** Summary for the *A*. *mongolicus* leaf unigenes.

Total number of reads	62,742,168
Total base pairs (bp)	5,646,795,120
Average read length (bp)	90
Total number of contigs	154,309
Mean length of contigs (bp)	304
N50 of contigs (bp)	471
Total number of unigenes	73,725
Mean length of unigenes (bp)	697
N50 of unigenes (bp)	844

**Fig 1:**
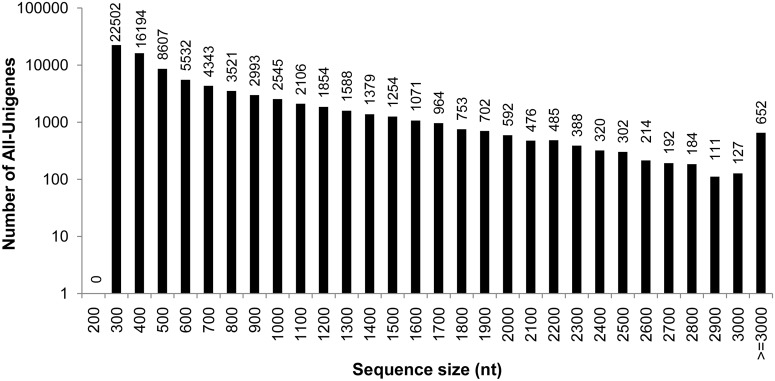
Size distribution of sequences in the All-Unigenes.

To obtain a comprehensive transcriptome database, our unigenes dataset was further assembled with the ESTs deposited in the GenBank database (the last update time: Feb. 8, 2014), and the transcriptome dataset (29,056 unique sequences) assembled in a previous study [13] using Phrap (Release 23.0) [21]. A total of 81,951 unigenes (designated All-Unigene) was obtained with a mean size of 665 bp, including 15,714 unigenes ≥1,000 bp (Fig 1). Compared with the leaf unigenes, the quantity of reads ≥1000 increased by 49% (from 10,560 to 15,714), although the mean length of unigenes in All-Unigene decreased slightly by 4.6% (from 697 to 665 bp).

### Functional annotation of *A*. *mongolicus* transcriptome sequences

**Fig 2:**
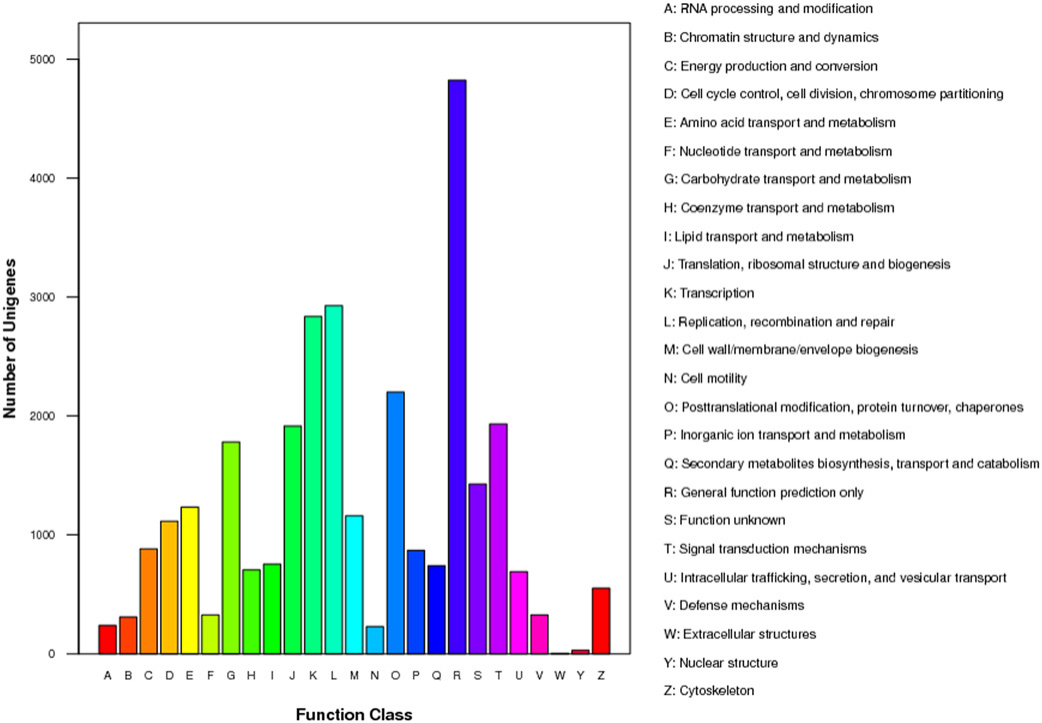
Histogram presentation of clusters of orthologous groups (COG) classification.

For the functional annotation, the All-Unigene sequences were aligned against NR (non-redundant protein sequences in NCBI), NT (Nucleotide collection), Swiss-Prot, KEGG (Kyoto Encyclopedia of Genes and Genomes database), COG (Clusters of Orthologous Groups of proteins) and GO (Gene Ontology) database using the BLASTX algorithm. A typical cutoff value of E<10^-5^ was used. A total of 59,814 sequences (73%) were annotated successfully (Table 2, Fig 2 and S2 Fig).

**Table 2:** Statistics of annotation results of All-Unigene sequences.

All-Unigene	NR	NT	Swiss-Prot	KEGG	COG	GO	All
81,951	53,948	54,693	30,621	28,378	16,467	41,377	59,814

**Fig 3:**
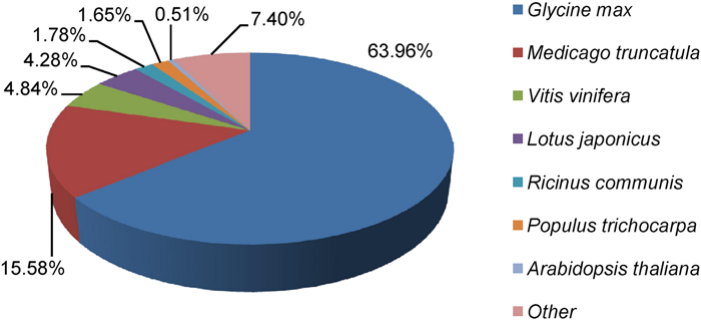
Species distribution of the sequences in All-Unigene.

To study the sequence conservation of *A*. *mongolicus* in other plant species, we analyzed the species distribution of the All-Unigene datasets by aligning sequences against NR database. The result shows that 64% of the distinct sequences have top matches (first hit) with the sequences from *Glycine max*, followed by *Medicago truncatula* (16%), *Vitis vinifera* (5%), and *Lotus japonicus* (4%) (Fig 3). As expected, more than 83% of the distinct sequences in the All-Unigene datasets have top matches (first hit) with the sequences from the plants in family Leguminosae.

After searching All-Unigene sequences against the protein databases using BLASTX (E-value < 0.00001) in the priority order of NR, Swiss-Prot, KEGG and COG, we extracted 53,835 coding sequences (CDS) from All-Unigene sequences and translated them into peptide sequences. For the sequences with no BLAST hits, we used ESTScan [22] to predict the 2,391 CDS and translated them into peptide sequences. In total, more than 56,000 CDS were predicted from All-Unigene sequences.

Taken together, the All-Unigene represented by far the most comprehensive transcriptome database of *A*. *mongolicus*, and the dataset was used as the reference transcriptome database in the gene expression analysis.

### Analysis of digital gene expression (DGE) libraries

To investigate the transcriptome response to drought stress in the leaves of *A*. *mongolicus*, the IlluminaHiSeq 2000 platform was used to perform high throughput tag-seq analysis on *A*. *mongolicus* leaf libraries that were constructed at 4 time-points before and during the 72 h period of PEG-6000 treatment. The samples collected at 0, 1, 24, and 72 h after PEG-6000 treatment were named libraries D1, D2, D3, and D4, respectively. The major characteristics of these libraries are summarized in Table 3. Approximately 7 million sequence tags per library were obtained. Prior to mapping these tag sequences to the reference transcriptome sequences, adaptor, low quality and single copy tags were filtered, resulting in more than a total of 6.9 million clean sequence tags per library. The clean tag data is deposited in NCBI’s Gene Expression Omnibus [23] and is accessible through GEO Series accession number GSE54251.

**Table 3:** Statistics of DGE sequencing.

Summary		D1	D2	D3	D4
Raw data	Total tags	7,624,805	7,046,950	7,059,565	7,598,720
Clean tags	Total number	7,486,538 (100.00%)	6,933,363 (100.00%)	6,963,563 (100.00%)	7,461,926 (100.00%)
	Total basepairs	366,840,362 (100.00%)	339,734,787 (100.00%)	341,214,587 (100.00%)	365,634,374 (100.00%)
All tag mapping to reference sequences	Total number	6,405,505	5,938,557	5,976,133	6,402,895
	Total % of clean tags	85.56%	85.65%	85.82%	85.81%
Unambiguous tag mapping to reference gene sequences (Perfect match)	Total number	5,278,533	4,931,825	4,945,585	5,339,304
	Total % of clean tags	70.51%	71.13%	71.02%	71.55%
Unique match	Total number	4,673,249	4,305,590	4,300,030	4,639,933
	Total % of clean tags	62.42%	62.10%	61.75%	62.18%
Unmapped Reads	Total number	1,081,033	994,806	987,430	1,059,031
	Total % of clean tags	14.44%	14.35%	14.18%	14.19%

Clean tags are tags that remained after filtering out low quality tags from raw data. Unambiguous tags are clean tags remaining after the removal of tags mapped to reference sequences from multiple genes.

To reveal the molecular events behind DGE profiles, we mapped the tag sequences (clean tags) of the four DGE libraries to the All-Unigene database of the *A*. *mongolicus* generated in the above-mentioned Illumina sequencing and assemblies. 70.51%–71.55% of the total clean tags were mapped unambiguously (perfectly) to the All-Unigene database (Table 3).

To estimate whether or not the sequencing depth was sufficient for the transcriptome coverage, the sequencing saturation was analyzed in the four libraries. The genes that were mapped by all clean tags increased with the total number of tags. However, when the sequencing counts reached 6 million tags or higher, the number of detected genes was nearly saturated (S3 Fig).

### Changes in gene expression profile during drought treatment

**Fig 4:**
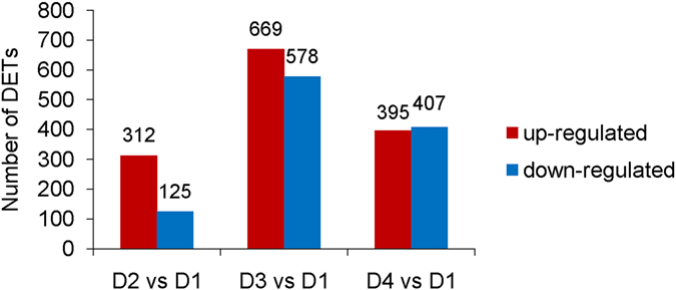
The up-regulated and down-regulated transcript numbers during drought treatment.

**Fig 5:**
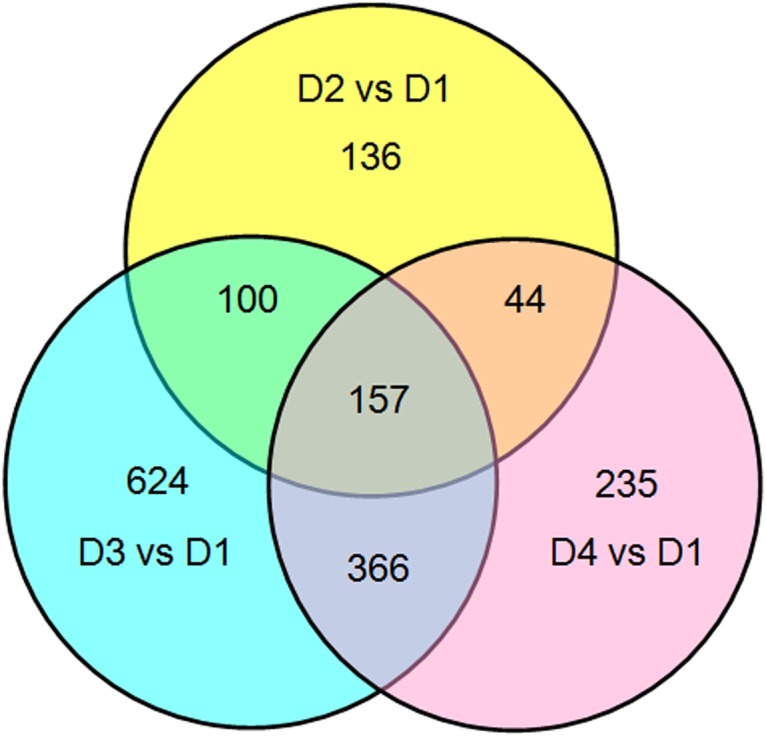
The Venn diagram analysis of the quantity of the 1664 DETs identified at different time points of drought treatment. One hundred and fifty-seven DETs were identified at the three time points, while some DETs were identified only at specific time-point. There were relatively large overlaps between the DETs identified at 24 h and 72 h time-points and small overlaps between the DETs identified at 1 h and 72 h time-points.

To identify the genes showing significant change in expression level during drought stress, the differentially expressed transcripts (DETs) between D1 and other three libraries (D2, D3 and D4) were identified using an algorithm developed by Audic *et al*. [24]. Our digital expression analysis identified 437 to 1,247 DETs with at least two-fold difference in expression levels during 72 h drought treatment and a false discovery rate (FDR) < 0.001 (Fig 4). The differential expression patterns among libraries revealed that the largest differences occurred between D1 and D3, and 669 up-regulated and 578 down-regulated DETs were identified in D3; while the smallest differences existed between D1 and D2, and only 312 up-regulated and 125 down-regulated DETs were identified in D2 (Fig 5).

To validate the gene expression patterns revealed by DGE analysis, quantitative real-time PCR assay were performed using the primers (S1 Table) designed according to sequences of the nine selected unigenes. All 9 transcripts showed similar expression patterns as the *in silico* differential analysis results from the DGE sequencing (S4 Fig). Thus, the DGE results were reliable for the identification of DETs during drought stresses in the present study. In addition, the production of expected fragment sizes using designed primers have also supported the reliability of the *de novo* assembly.

### Functional analysis of differentially expressed genes based on RNA-Seq data

**Fig 6:**
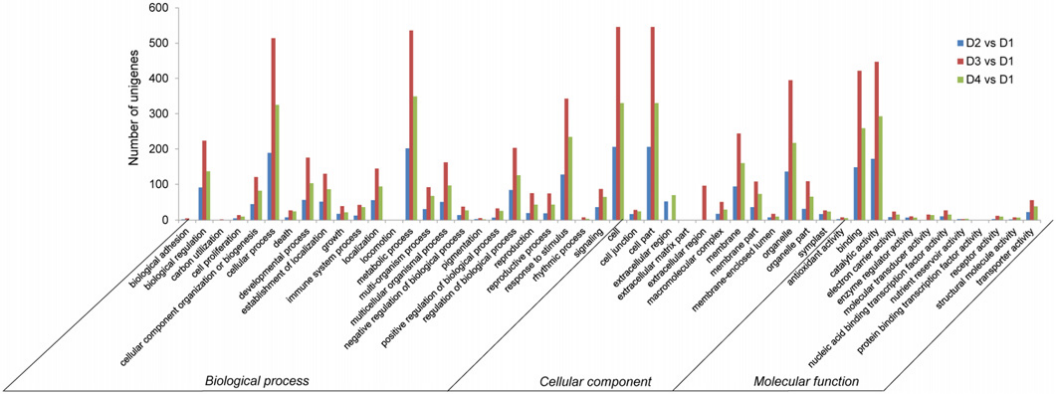
Gene Ontology (GO) functional classification analysis of differentially expressed transcripts (DETs) based on RNA-Seq data. GO functional classification analysis of DETs in D2 vs D1, D3 vs D1, and D4 vs D1. Based on sequence homology, 2,573 differentially expressed genes could be categorized into three main categories (biological process, cellular component, and molecular function), which include 25, 13, and 12 functional groups, respectively.

Gene Ontology functional classification analyses were performed to classify the functions of the DETs during drought treatment [25]. Based on sequence homology, all DETs could be categorized into 50 functional groups (Fig 6). In the three main categories [Biological process (BP), Cellular component (CC), and Molecular function (MF)] of the GO classification, there were 25, 13, and 12 functional groups, respectively (Fig 6). Among these groups, the terms metabolic process (GO: 0008152), cell part (GO: 0044464), and catalytic activity (GO: 0003824) were dominant in each of the three main categories, respectively. We also noticed a considerable quantity of genes from the functional groups of cellular process (GO: 0009987), and response to stimulus (GO: 0050896), organelle (GO: 0043226), membrane (GO: 0016020), binding (GO: 0005488), and transporter activity (GO: 0005215) (Fig 6).

We also conducted Gene ontology enrichment analysis to identify significantly enriched BP, CC, or MF terms in DETs during drought treatment. There were 4, 21 and 24 enriched BP terms at time-points 1, 24 and 72 h, respectively (S2 Table). We studied ontology terms enriched specifically in one or more time-points. Although no BP terms were found to be present across all time-points, 13 BP terms were enriched in both 24 h and 72 h time-points, showing a higher level of similarity between the 24 h and 72h time-points. The enriched ontology terms at time-point 1h included "GO:0033273: response to vitamin", and one of its sub-processes "GO:0033591: response to L-ascorbic acid", and two sub-processes of "GO:0009664: plant-type cell wall organization", indicating change in cell wall structure was induced by drought treatment at the first 1 h period. The 13 common GO terms in 24 h and 72 h can be classified into three categories (Table 4). The first category associated with "response to stimulus", including "response to hormone stimulus" and "response to jasmonic acid stimulus". The second category associated with "secondary metabolite biosynthetic process", including "phenylpropanoid biosynthetic process". The third category was related with "cellular component assembly", including "chromatin assembly" and "nucleosome assembly ". This suggests that hormone response, phenylpropanoid biosynthetic process and nucleosome assembly were involved in the drought response in *A*. *mongolicus* leaves in 24 h to 72 h period. The enriched CC terms mainly included cell wall, apoplast and nucleosome (GO term: cell wall, cell periphery, apoplast, nucleosome, etc). The enriched MF terms included oxidoreductase activity, ethylene binding, etc.

**Table 4:** Biological processe (BP) terms significantly affected by drought treatment in *A*. *mongolicus* leaves during 24 to 72 h period.

Category	GO terms
Metabolic process	
	Secondary metabolite biosynthetic process
	Phenylpropanoid metabolic process
	Phenylpropanoid biosynthetic process
Response to stimulus	
	Response to stimulus
	Response to endogenous stimulus
	Response to chemical stimulus
	Response to hormone stimulus
	Response to jasmonic acid stimulus
Cellular component assembly	
	Chromatin assembly
	Nucleosome organization
	Nucleosome assembly
	Protein-DNA complex assembly
	Protein-DNA complex subunit organization

The significance of effects was determined by examining enrichment of gene ontology (GO) terms associated with differentially expressed transcripts (DETs) versus the corresponding GO terms in the whole transcriptome database distribution using a hypogeometric test and a threshold of Benjamini and Hochberg false discovery rate (FDR) corrected at *P* ≤ 0.05.

To further identify significantly altered biochemical pathways during drought treatment, we mapped the genes to terms in the KEGG database (http://www.genome.ad.jp/kegg/) [26] and compared this with the whole transcriptome background, with a view of searching for the genes involved in metabolic or signal transduction pathways that were significantly enriched. Among all genes with a KEGG pathway annotation, 227, 636 and 425 DETs were identified between the D2 and D1 libraries, D3 and D1 libraries, and D4 and D1 libraries, respectively (S3 Table).

There were 24, 30 and 25 enriched pathways at time-points 1, 24 and 72 h, respectively (S3 Table). Notably, the enrichment of 19 pathways was always observed in analyses from D2 vs D1, D3 vs D1, and D4 vs D1, and these pathways included 1) biosynthesis of secondary metabolites, including stilbenoid, diarylheptanoid and gingerol biosynthesis, phenylpropanoid biosynthesis, diterpenoid biosynthesis, and flavone and flavonol biosynthesis, 2) protein processing in endoplasmic reticulum, 3) plant hormone signal transduction (S5 Fig), 4) nitrogen metabolism, including alanine, aspartate and glutamate metabolism, 5) endocytosis, 6) plant-pathogen interaction, 7) lipid metabolism, including ether lipid metabolism, and 8) carbohydrate metabolism, including pentose and glucuronate interconversions. The results suggest that, besides the hormone signal transduction and secondary metabolites biosynthesis, amino acid metabolism, lipid metabolism, sugar metabolism, endocytosis, and plant-pathogen interaction were also involved in the drought response in *A*. *mongolicus* leaves.

Meanwhile, enrichment of genes in specific pathways was only observed in analysis of specific time-point. For example, the enrichment of genes in pathway "Tryptophan metabolism", "Fatty acid elongation", and "Linoleic acid metabolism" were only observed in the analyses from D2 vs D1, D3 vs D1, and D4 vs D1, respectively. This indicates that there are considerable differences between the physiological processes in the early and prolonged stages of drought stress response.

### Candidate transcription factors associated with drought response in *A*. *mongolicus*

Various drought-responsive transcription factors and their expression patterns are listed in Table 5. These 36 transcription factors belong to HD-ZIP, bZIP, WRKY, ERF/AP2, bHLH, C3H, C2C2-CO-like, NF-Ya, TCP, MYB, HSF, and NAC families. Although the four bZIP family transcription factors were all up-regulated by drought treatment, and the four HD-ZIP family transcription factor genes were all down-regulated, the expression patterns of the other genes varied among the transcription factor genes.

**Table 5:** Putative transcription factor genes regulated by drought stress in *A*. *mongolicus*.

Gene ID	Annotation	TF family	log2 ratio		
			1 h	24 h	72 h
CL5346.Contig1_All	Homeobox-leucine zipper protein HDG11-like	HD-ZIP	-0.58	**-1.03** ^a^	-0.87
Unigene12132_All	Homeobox-leucine zipper protein ATHB-40-like	HD-ZIP	-1.10	-0.54	**-2.14**
Unigene33019_All	Homeobox-leucine zipper protein ANTHOCYANINLESS 2-like	HD-ZIP	-0.62	**-1.09**	-0.61
Unigene2219_All	Homeobox-leucine zipper protein ATHB-7	HD-ZIP	-0.71	**-1.15**	**-1.35**
Unigene6504_All	bZIP transcriptional repressor ROM1	bZIP	1.22	**1.83**	0.97
Unigene33132_All	Transcription factor bZIP48	bZIP	**1.66**	**1.79**	**1.68**
Unigene2931_All	bZIP protein	bZIP	**1.78**	**2.65**	**1.44**
Unigene392_All	Probable transcription factor PosF21	bZIP	**2.37**	**2.66**	**2.84**
CL4621.Contig4_All	Probable WRKY transcription factor 3-like	WRKY	**1.78**	**-0.66**	**-0.77**
Unigene5908_All	Probable WRKY transcription factor 40-like isoform 1	WRKY	**1.40**	-0.46	-0.05
CL2023.Contig1_All	Probable WRKY transcription factor 33	WRKY	**1.27**	**-1.37**	**-1.86**
CL4465.Contig3_All	Probable WRKY transcription factor 33	WRKY	1.26	-3.20	-2.31
Unigene40346_All	Probable WRKY transcription factor 41-like	WRKY	2.29	-7.43	-7.43
Unigene5149_All	Transcription factor	WRKY	0.04	**-1.65**	**-1.64**
Unigene45786_All	Probable WRKY transcription factor 41-like	WRKY	-1.04	**-1.68**	-1.08
CL1965.Contig1_All	Transcription factor WRKY	WRKY	-1.53	**-4.34**	-0.86
Unigene22759_All	Ethylene-responsive transcription factor ERF061-like	ERF/AP2	**-1.00**	**1.57**	**1.52**
Unigene30830_All	Ethylene-responsive transcription factor 9-like	ERF/AP2	-0.74	**-2.26**	-1.54
Unigene1090_All	ERF and B3 domain-containing transcription repressor TEM1	ERF/AP2	**1.58**	-0.22	-0.09
Unigene25323_All	Ethylene-responsive transcription factor 1A	ERF/AP2	0.82	**-2.35**	**-1.41**
Unigene28591_All	Dehydration-responsive element-binding protein 3-like	ERF/AP2	-0.41	**-1.53**	-0.91
Unigene237_All	Dehydration-responsive element-binding protein 2C-like	ERF/AP2	1.34	0.93	1.37
Unigene1202_All	Ethylene-responsive transcription factor RAP2-1-like	ERF/AP2	-0.50	**1.05**	0.24
CL2735.Contig1_All	Ethylene-responsive transcription factor 4-like	ERF/AP2	-0.90	**-1.10**	**-1.20**
Unigene7039_All	Ethylene-responsive transcription factor 4-like	ERF/AP2	-0.70	**-1.33**	**-1.33**
CL1129.Contig2_All	AP2/ERF and B3 domain-containing transcription repressor TEM1-like	ERF/AP2	**1.48**	-0.93	-0.25
Unigene13243_All	Transcription factor bHLH87-like	bHLH	**4.37**	**2.71**	0.01
CL1914.Contig1_All	Transcription factor bHLH49-like	bHLH	**1.34**	**1.59**	**1.60**
Unigene31971_All	Zinc finger CCCH domain-containing protein 20-like isoform 1	C3H	**-1.10**	-0.35	-0.60
Unigene13412_All	Zinc finger CCCH domain-containing protein 23-like	C3H	**1.21**	**3.24**	**1.51**
CL6133.Contig2_All	Zinc finger protein CONSTANS-LIKE 6-like	C2C2-CO-like	0.03	**2.32**	**2.52**
CL4100.Contig3_All	Nuclear transcription factor Y subunit A-1-like	NF-Ya	**-1.20**	**-1.08**	-0.58
CL714.Contig2_All	NAC domain-containing protein 72-like isoform 1	NAC	0.32	-0.76	**-1.43**
Unigene26008_All	Transcription factor TCP9-like	TCP	**1.87**	1.09	0.65
Unigene18198_All	MYB transcription factor MYB124	MYB	**1.43**	**1.49**	**2.42**
Unigene5756_All	Heat shock transcription factor (HSF)	HSF	**1.31**	**1.48**	**1.49**

^**a**^ Significant difference (FDR ≤ 0.001 and the absolute value of log2 ratio ≥ 1) in relative levels are shown in boldface.

## Discussion

As one of the two species in genus *Ammopiptanthus*, the only broadleaf evergreen plant family in the Mid-Asia desert, *A*. *mongolicus* can survive in the environments with alternating extreme drought, cold, and heat conditions, and thus, *A*. *mongolicus* could be a suitable model plant for investigating the biochemical, physiological, and molecular mechanism behind tolerance of trees to individual or combined environmental stresses [9,13]. Since the genomic information of *A*. *mongolicus* is not available in the public database, so far, establishment of an extensive and high quality transcriptome database rapidly using the NGS technology will be one of the essential tools and, a good start, for elucidation of the molecular mechanism underlying tolerance of *A*. *mongolicus* and other non-model plant species. In the present study, RNA-seq technology was applied for *A*. *mongolicus* transcriptome profiling, in which the transcriptome was sequenced on the Illumina HiSeq2000 sequencing platform. Based on *de novo* assembly with the ESTs from Genbank and the 454 pyrosequencing data, we have established an extensive transcriptome database containing 81,951 unigenes (Table 2).

Of the three mainstream high throughput sequencing technologies, namely, 454 pyrosequencing, Solexa/Illumina and Solid, the first one had been used for transcriptome sequencing most frequently [27–29], probably owing to the longest sequencing length. However, due to the increases in sequencing length of Solexa/Illumina reads to 150 bp or more, more transcriptome sequencing studies using Illumina technology have been published since 2012 [30,31], indicating that the Illumina sequencing is becoming the first choice in the field of genomics. In the present study, we assembled the unigenes obtained by Illumina sequencing, the unigenes obtained previously by 454 pyrosequencing, and the ESTs from GenBank. The main parameters of the resulting All-Unigene database were clearly better than the unigenes assembled solely from Solexa reads, or from the 454 reads. Functional annotation by aligning to NR, NT, UNIPROT, GO, COG, KEGG, species conservation analysis, and CDS prediction also demonstrated the All-Unigene can be used as the reference transcriptome sequence for RNA-Seq analysis.

Whole genome gene expression profiling will provide basic data for understanding the gene regulation strategy of the plant under an environmental stress. The most popular method in this field is gene chip / microarray; however, as sequencing costs decrease, NGS will likely replace microarrays for gene expression analysis in the future, mainly due to improved dynamic range, and additional capabilities for detecting expressed single nucleotide variants, and transcript isoform switches [32]. Several studies compared the two gene profiling approaches and found that the two technologies agreed frequently in both detection and differential expression, with slight advantages for both [33,34]. Because the NGS do not need the genome sequence information, most genome-wide expression studies performed in non-model species employ RNA-Seq technology, while the microarray continues to be used frequently in model organisms such as *Arabidopsis*. In our study, DGE, a tag based RNA-Seq technology, was used to reveal the gene regulation network in *A*. *mongolicus* under drought stress.

Drought is one of the vital factors limiting plant growth and distribution, and will become increasingly important in many regions because of the ongoing global climate change. Over the long evolutionary period, plants have evolved various mechanisms helping them to survive in water deficient conditions. These strategies include reducing the water deficit by developing huge root systems to take up water deep in the soil, minimizing water losses by stomatal closure or producing a thick cuticle, and accumulating osmoprotective substances like proline [35,36]. In recent years, *A*. *mongolicus* has attracted increasing attention from botanists and ecologists [9,13]. *A*. *mongolicus* is an ecologically important shrub in the Mid-Asia arid region and desert, which represent a fragile ecosystem with little vegetation. Furthermore, *A*. *mongolicus* is a relic tree of the Tertiary period, with higher tolerance to alternating drought, heat and freezing stresses. Given the success of *A*. *mongolicus* in its stressful growth environment, the secrets to this success remain to be elucidated from the plant's genome sequence and genome expression patterns.

The results from our time-course DGE analysis in drought-treated *A*. *mongolicus* leaves showed that the DETs were effectively identified across a wide range of transcript abundance. Of the three time-points, i.e., 1, 24 and 72 h, fewer DETs were found at the time-point 1 h (437) than those at the time-point 24 h (1,247) and 72 h (802) (Fig 4), indicating that the alterations in gene expression at the time-points 24 h and 72 h were more similar. This observation is supported by the result of Venn diagram analysis (Fig 5) of the 1,664 DETs. We speculated that the change in gene expression became greater during the first 24 h, and then fell gradually.

Upon exposure to drought stress, plants exhibit a large number of responses at the molecular, cellular and whole-plant levels, and the metabolic adjustment under drought stress can be revealed by GO and KEGG enrichment analysis. In the present study, several biological processes were enriched in both GO and KEGG analysis. These biological processes were classified into two categories: "biosynthesis of secondary metabolites (including phenylpropanoid biosynthesis)" and "response to hormone stimulus and plant hormone signal transduction". Phenylpropanoid biosynthesis has been demonstrated to contribute to various aspects of plant biotic and abiotic responses and synthesis of phenylpropanoid-based polymers, such as lignin and flavonoids [37]. The drought-induced alteration in phenylpropanoid biosynthesis pathway in *A*. *mongolicus* leaves may contribute to cell wall modification (enriched in GO analysis) through affecting lignin biosynthesis, regulate anthocyanin biosynthesis by influencing anthocyanidins formation, and promote plant cuticle formation through providing cutin.

Plant hormones play central roles in adaptation to changing environments by influencing growth, development, and source / sink transitions via gene regulation. Although abscisic acid (ABA) is the well-documented stress-responsive hormone, the roles of the other hormones in stress response have attracted emerging attention [38]. The KEGG analysis in our study revealed that multiple hormones were involved in drought response in *A*. *mongolicus* leaves, including auxin, cytokinin, gibberellin, ethylene, brassinosteroids, jasmonate, and salicylic acid (S5 Fig). These hormone-associated pathways exhibited distinct pattern changes during drought treatment, suggesting the importance of a complicated and balanced hormone signal transduction pathway in drought response in *A*. *mongolicus*.

To form a basis for understanding the function of common drought responses in drought-resistant plants, we compared the GO and pathway enrichment results of drought-induced DETs identified in *A*. *mongolicus* to those of *Reaumuria soongorica* and Sorghum bicolor. *R*. *soongorica* is an extreme xerophyte shrub widely distributed in the desert regions similar to habitats of *A*. *mongolicus*. A recent transcriptomic study revealed that transcripts related to biosynthesis of secondary metabolites, plant hormone signal transduction, plant-pathogen interaction, protein processing in endoplasmic reticulum, and starch and sucrose metabolism were differentially expressed in drought-treated *R*. *soongorica* leaves [39]. The results are highly consistent with that of *A*. *mongolicus*, which is not surprising given the similar habitats of the two species. Sorghum is the cereal crop best adapted to water-limited environments. The trancriptomic analysis of the drought stress responses in sorghum identified several enriched GO terms from drought-induced DETs, some of which were similar with that of *A*. *mongolicus*, including plant hormone (abscisic acid) mediated signaling pathway, carbohydrate catabolic process [40]. However, it is noteworthy that secondary metabolite biosynthetic process and phenylpropanoid biosynthetic process were enriched in drought-induced DETs identified in *A*. *mongolicus* and *R*. *soongorica*, but not in identified in drought stressed *Sorghum bicolor*. These results indicate that there are some common drought responses among drought-resistant plants; on the other hand, certain plant exhibits specific characteristics.

Transcription factors have been suggested to play important regulatory roles in environmental stress-induced gene regulation network [17,41]. In the present study, nearly 40 transcript-encoding putative transcription factors were regulated under drought treatment, including several members of HD-ZIP, bZIP, WRKY, AP2/ERF, and bHLH family. Characterized by a DNA-binding HD and a protein—protein interaction Zip domain, the HD-Zip family transcription factors are an abundant group of transcription factors exclusively found in plants [42]. Several studies demonstrated that HD-Zip I and HD-Zip II proteins play a regulatory role in environmental stress [43]. It has been demonstrated that *ATHB7* played an important role in the primary response to drought, functioning as mediators of a negative feedback effect on ABA signalling [44]. Among the four HD-Zip transcription factors down-regulated under drought stress, an *ATHB7* homologue may participate in drought-induced ABA signal transduction in *A*. *mongolicus* in a similar manner with *ATHB7*.

Previous reports have demonstrated that WRKY transcription factors participated in various abiotic and biotic stress responses [45,46]. For example, *AtWRKY33* was reported to not only be important for plant resistance to necrotrophic pathogens, but also be involved in regulation of the heat-induced ethylene-dependent response. *AtWRKY40* was demonstrated to act as a central negative regulator in ABA signalling through directly inhibiting the expression of several important ABA responsive genes, such as *AtABF4* [47]. We found two *AtWRKY33* homologues and one *AtWRKY40* homologue that exhibited a rapid and transient induced expression pattern upon drought treatment (Table 5), indicating their potential roles in controlling the signalling processes associated with transcriptional reprogramming in plants encountering environmental stress.

## Conclusions

In the present study, we performed rapid and cost-effective transcriptome and DGE analyses using Illumina sequencing technology. Through deep-sequencing and assembly, a comprehensive transcriptome database was obtained. Sequencing and bioinformatics analysis of DGE identified a batch of drought-responsive transcripts, including 36 transcription factors, and revealed several features of the metabolic adjustment strategy in *A*. *mongolicus* upon drought stress. These findings provide a substantial contribution to existing nucleotide sequence resources for *A*. *mongolicus*, will accelerate studies on molecular mechanism underlying the drought-stress tolerance of *A*. *mongolicus*, and are helpful for identification the critical genes that play core roles in abiotic stress response in *A*. *mongolicus*. Further investigation should focus on the roles of these drought-responsive transcription factors and the enriched biological processes under drought stress.

## Materials and Methods

### Ethics statement

The seeds of *Ammopiptanthus mongolicus* were collected, and research activities were scientifically conducted under the permits issued by Zhongwei Forest Bureau. The experimental procedures were approved by the Ethics Committee for Plant Experiments of Minzu University of China and the State Forestry Administration, China.

### Plant material and stress treatment

Seeds of *A*. *mongolicus*, collected from the desert region in Zhongwei City, Ningxia Autonomous Region, China, were surface-sterilized with ethanol, soaked in water for 48 h at 25°C, and then sown in 9 cm diameter commercial pots containing vermiculite and perlite (1:1, w/w) in a greenhouse at approximately 25°C and 35% relative humidity under a photosynthetic photon flux density of 120 μmol m^-2^ s^-1^ with a photoperiod of 16 h light and 8 h dark. The seedlings were watered in a three-day interval with half-strength of Hoagland’s solution. Two to three weeks after germination, one part of the seedlings were harvested and used for Illumina transcriptome sequencing, and the other part of seedlings were used for RNA-Seq sequencing (gene expression study).

For stress treatments, the seedlings were randomly divided into four groups. The first group was harvested before treatment and served as the control (0 h), whilst the second (1 h), the third (24 h), and the fourth (72 h) was irrigated with 20% PEG-6000 for 1, 24, and 72 h, respectively. The leaf samples of all groups were used for RNA-Seq sequencing. All tissue samples collected were snap-frozen immediately in nitrogen and stored at -80°C until further processing.

### RNA extraction and cDNA library preparation

Total RNA was extracted using TRIzol reagent according to the manufacturer’s protocol (Invitrogen, Burlington, Canada). The quality and purity of RNA were assessed by determining the absorbance at 280, 260 and 230 nm using NanoDrop 2000 Spectrophotometer (Thermo Scientific, Wilmington, USA). RNA was only used when the OD260/280 was greater than 1.8. RNA integrity was checked with the Agilent 2100 Bioanalyzer (Agilent Technologies, Santa Clara, USA) and the value was no less than 7. The extracted total RNA was stored at -80°C for later use.

After DNase I digestion, magnetic beads with Oligo (dT) were used to isolate mRNA from the total RNA. The mRNA was cleaved into short fragments with divalent cation at elevated temperature. Then, cDNA was synthesized using the mRNA fragments as templates. Short fragments were purified and dissolved with EB buffer for end reparation and single nucleotide A (adenine) addition. The short fragments were ligated to random hexamer adapters. The final products were size selected and enriched by PCR to create the final cDNA library for transcriptome sequencing and DGE sequencing. Agilent 2100 Bioanaylzer (Agilent Technologies, Santa Clara, USA) and Bio-Rad my IQ2 Real-Time PCR System (Bio-Rad Laboratories, Hercules, USA) were used for quantification and quality control of the sample library during the quality control steps.

### Quantitative real-time PCR analysis

Approximately 1 μg of DNase I-treated total RNA was converted into single-stranded cDNA using M-MLV Reverse Transcriptase (Promega, Madison, USA). The cDNA products were then diluted 20-fold with deionized water before use as templates in real-time PCR. The quantitative reaction was performed on an MyiQ2 two-color real-time PCR detection system (Bio-Rad Laboratories, Hercules, USA) using the GoTaq qPCR Master Mix (Promega, Madison, USA). The reaction mixture (20 μL) contained 2× qPCR Master Mix, 0.9 μM each of the forward and reverse primers, and 1 μL of template cDNA. PCR amplification was performed under the following conditions: 95°C for30 s, followed by 40 cycles of 95°C for 5 s and 60°C for10 s. Three independent biological replicates for each sample and three technical replicates of each biological replicate were analyzed in quantitative real-time PCR analysis. The gene expressions of selected unigenes were normalized against an internal reference gene, 18S rRNA. The relative gene expression was calculated using the 2^-ΔΔCt^ method [48]. All primers used in this study are listed in S1 Table.

### Transcriptome sequencing and assembly

The sequencing raw reads were cleaned by removing reads with adaptors, reads with unknown nucleotides larger than 5%, and low quality reads. Transcriptome *de novo* assembly was carried out with Trinity software [20]. In brief, Trinity firstly assembled the reads into unique sequences of transcripts, known as contigs, clustered the contigs into clusters and constructed complete *de Bruijn* graphs for each cluster, and then partitioned the full read set among these disjoint graphs. Finally, Trinity processed the individual graphs in parallel, tracing the path that reads and pairs of reads take within the graph, ultimately reporting full-length transcripts for alternatively spliced isoforms, and teasing apart transcripts that corresponds to paralogous genes. The final sequences of trinity assembly were called Unigenes.

### Functional annotation of unigenes

To perform the functional annotation of the unigene sequences, blastx alignment between the unigenes and protein databases NR (http://www.ncbi.nlm.nih.gov), Swiss-Prot (http://www.expasy.ch/sprot), KEGG (http://www.genome.jp/kegg) [27] and COG (http://www.ncbi.nlm.nih.gov/COG) [49] was conducted, and the best aligning results are used to determine the direction. If results of different databases conflict with each other, a priority order of NR, Swiss-Prot, KEGG, and COG was followed. If a unigene did not aligned within any of the above databases, the software named ESTScan [22] was used to determine the sequence direction. Blastn alignment between the unigenes and nucleotide databases NT was also conducted to annotate the unigenes sequences. Phrap (release 23.0, parameters:-repeat_stringency 0.95-minmatch 35-minscore 35) [21] was used to assemble the unigenes with the *A*. *mongolicus* ESTs.

### DGE sequencing and analysis

Tag libraries were prepared at the different time-points (0, 1, 24, and 72 h) during drought treatment. Briefly, mRNA was purified with magnetic oligo (dT) beads from DNase I-digested total RNA. To minimize sampling bias, the total RNA sample at each time-point was pooled from three independent experiments equally. The mRNA was interrupted into short fragments. Then, the first strand and the second strand cDNAs were synthesized, and purified with magnetic beads. End reparation and 3'-end single nucleotide addition was conducted. Then, sequencing adaptors were ligated to the fragments. The fragments were size selected and enriched by PCR amplification. At last, the library was sequenced using Illumina HiSeq 2000 and 50 bp reads (DGE tag) were obtained.

The raw reads were cleaned as described above to get the clean reads. Clean reads were mapped to reference transcriptome sequences using SOAPaligner/SOAP2 [50]. No more than two mismatches were allowed in the alignment. The expression level for each gene was determined by the numbers of reads uniquely mapped to the specific gene and the total number of uniquely mapped reads in the sample. The gene expression level was calculated by using RPKM [51] method (Reads Per kb per Million reads).

## Supporting Information

S1 Fig Size distribution of the contigs (A) and unigenes (B). (DOCX)

S2 Fig Histogram presentation of Gene Ontology classification. The results are summarized in three main categories: biological process, cellular component and molecular function. The right y-axis indicates the number of genes in a category. The left y-axis indicates the percentage of a specific category of genes in that main category.
(DOCX)

S3 Fig Relationship between the number of detected genes and sequencing amount. When the sequencing amount reaches 6 million or higher, the growth curve of detected genes flattens, indicating that the number of detected genes tends to saturation.
(DOCX)

S4 Fig Gene expression levels revealed by qRT-PCR (red column) and RNA-Seq (blue column). Data from qRT-PCR are means of three replicates and bars represent SE.
(DOCX)

S5 Fig KEGG enrichment analysis revealed that multiple hormones signal transduction pathways were involved in the drought response in *A*. *mongolicus* leaves. The up-regulated genes are boxed in red and the down-regulated genes are boxed in green. (A) Auxin, (B) Cytokinine, (C) Gibberellin, (D) Abscisic acid, (E) Ethylene, (F) Brassinosteroid, (G) Jasmonic acid, and (H) Salicylic acid.
(DOCX)

S1 Table Primer sequences used for qRT-PCR analysis. (DOCX)

S2 Table GO enrichment analysis of the DETs during drought treatment. (XLSX)

S3 Table KEGG enrichment analysis of the DETs during drought treatment. (XLS)
