# Supplementary figures and images for: Transcriptomic Analysis of Drought Stress Responses in Ammopiptanthus mongolicus Leaves Using the RNA-Seq Technique
Source: PLoS One. 2015 Apr 29;10(4):e0124382. doi: 10.1371/journal.pone.0124382 (PMC4414462; doi:10.1371/journal.pone.0124382)

|  | A | B |
| --- | --- | --- |
|  | 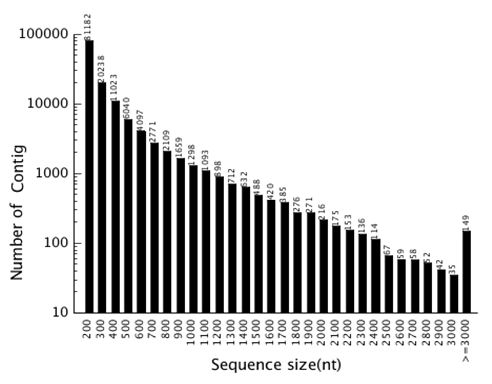 | 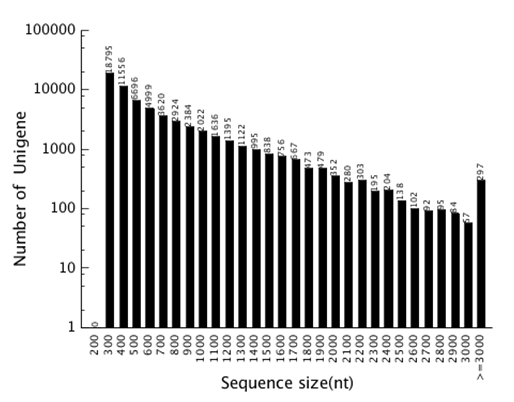 |

**Figure S1** **Size distribution of the contigs (A) and unigenes (B).**

Supplement: S1 Fig — (DOCX) [file pone.0124382.s001.docx]
