# Supplementary material for: Transcriptomic Analysis of Drought Stress Responses in Ammopiptanthus mongolicus Leaves Using the RNA-Seq Technique
Source: PLoS One. 2015 Apr 29;10(4):e0124382. doi: 10.1371/journal.pone.0124382 (PMC4414462; doi:10.1371/journal.pone.0124382)
Supplement: S2 Fig — The results are summarized in three main categories: biological process, cellular component and molecular function. The right y-axis indicates the number of genes in a category. The left y-axis indicates the percentage of a specific category of genes in that main category. (DOCX) [file pone.0124382.s002.docx]

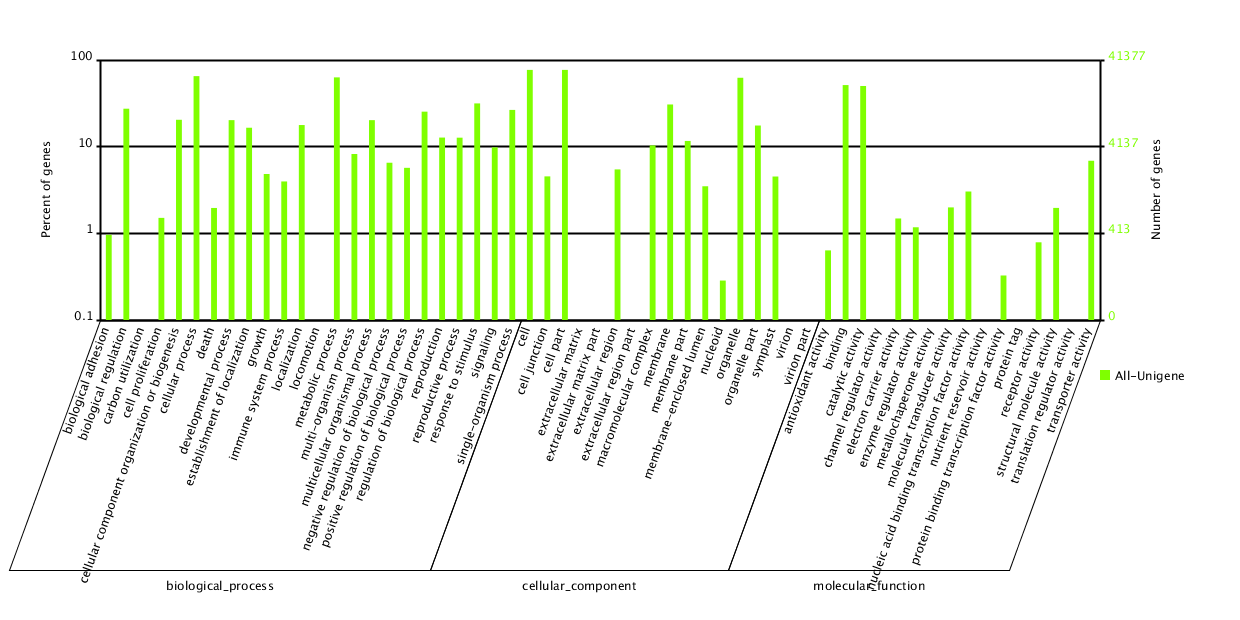


0

4137

41377

413

**Figure S2 Histogram presentation of Gene Ontology classification.** The results are summarized in three main categories: biological process, cellular component and molecular function. The right y-axis indicates the number of genes in a category. The left y-axis indicates the percentage of a specific category of genes in that main category.
