# Supplementary material for: Transcriptomic Analysis of Drought Stress Responses in Ammopiptanthus mongolicus Leaves Using the RNA-Seq Technique
Source: PLoS One. 2015 Apr 29;10(4):e0124382. doi: 10.1371/journal.pone.0124382 (PMC4414462; doi:10.1371/journal.pone.0124382)
Supplement: S3 Fig — When the sequencing amount reaches 6 million or higher, the growth curve of detected genes flattens, indicating that the number of detected genes tends to saturation. (DOCX) [file pone.0124382.s003.docx]

|  | D1 | D2 |
| --- | --- | --- |
|  | 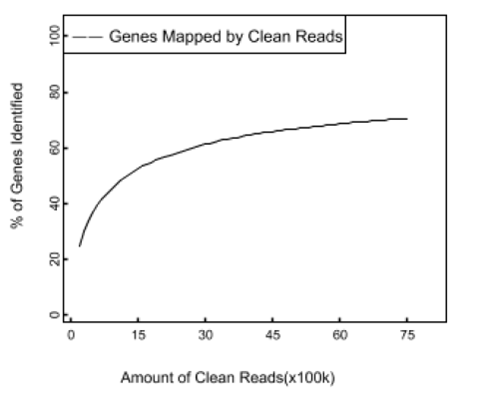 | 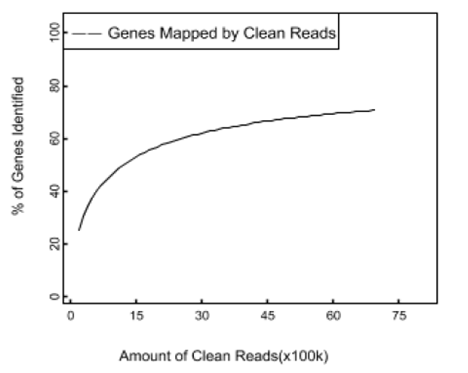 |
|  | D3 | D4 |
|  | 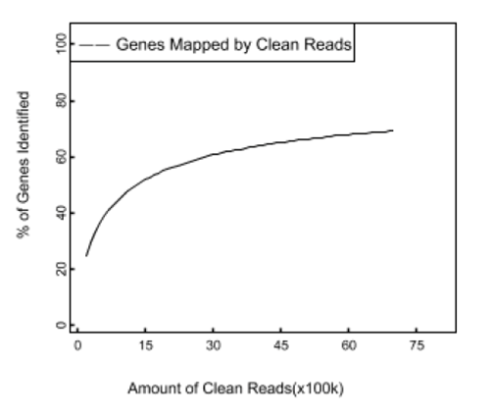 | 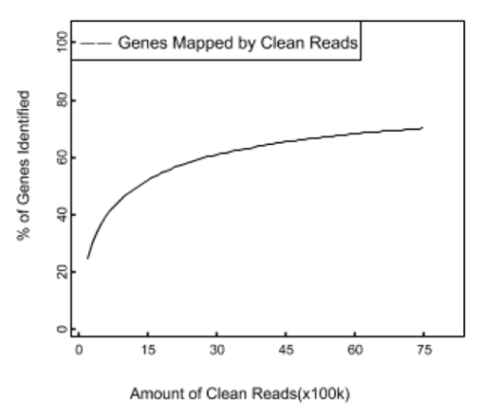 |

**Figure S3 Relationship between the number of detected genes and sequencing amount.** When the sequencing amount reaches 6 million or higher, the growth curve of detected genes flattens, indicating that the number of detected genes tends to saturation.
