# Supplementary material for: Transcriptomic Analysis of Drought Stress Responses in Ammopiptanthus mongolicus Leaves Using the RNA-Seq Technique
Source: PLoS One. 2015 Apr 29;10(4):e0124382. doi: 10.1371/journal.pone.0124382 (PMC4414462; doi:10.1371/journal.pone.0124382)
Supplement: S4 Fig — Data from qRT-PCR are means of three replicates and bars represent SE. (DOCX) [file pone.0124382.s004.docx]

| CL2929.Contig1_All:  Nicotianamine synthase 3 | 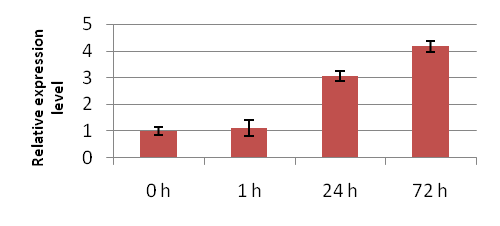 | 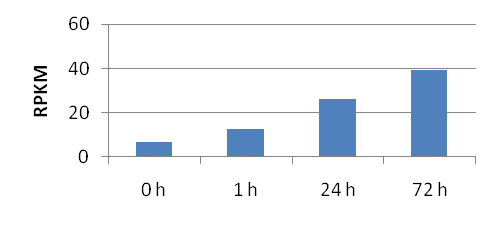 |
| --- | --- | --- |
| CL3605.Contig2_All:  Peroxidase superfamily protein | 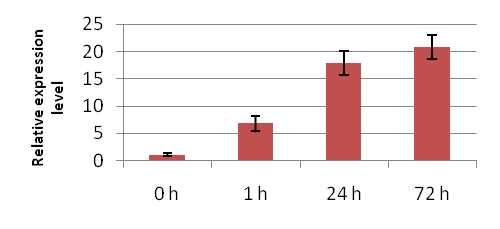 | 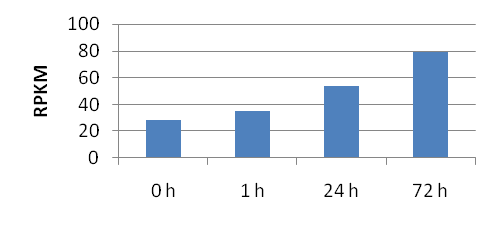 |
| CL6044.Contig2_All:  alcohol dehydrogenase 1 | 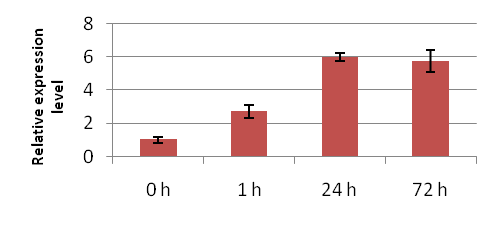 | 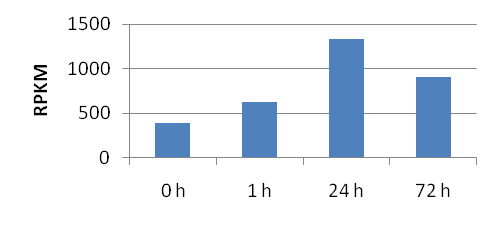 |
| Unigene15170_All  germin-like protein 1 | 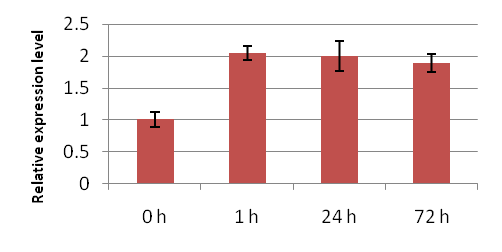 | 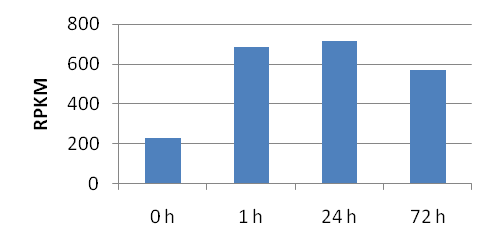 |
| Unigene35165_All:  beta galactosidase 1 | 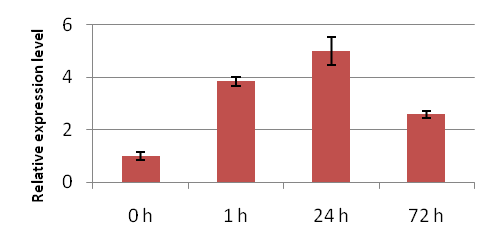 | 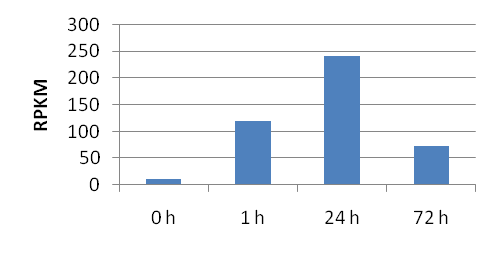 |
| Unigene2216_All:  E3 ubiquitin-protein ligase RMA1H1-like isoform 1 | 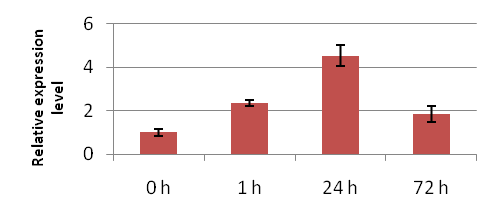 | 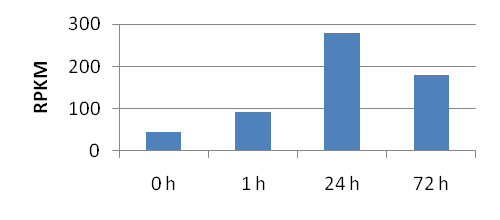 |
| Unigene1196_All:  expansin A4 | 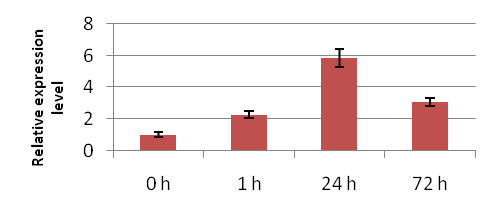 | 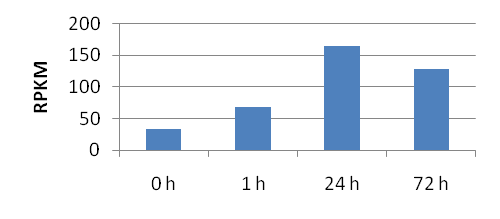 |
| Unigene16704_All:  plant U-box 26 | 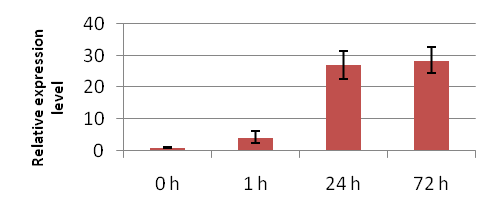 | 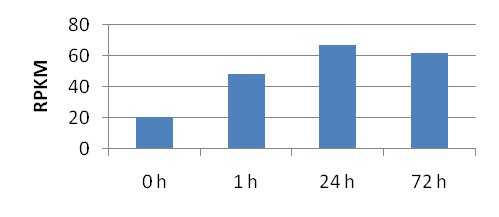 |
| Unigene36201_All:  lipid transfer protein 3 | 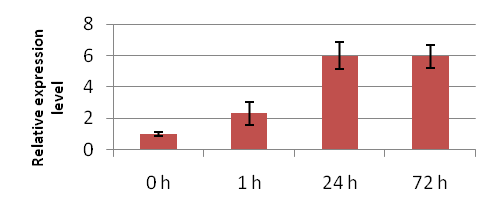 | 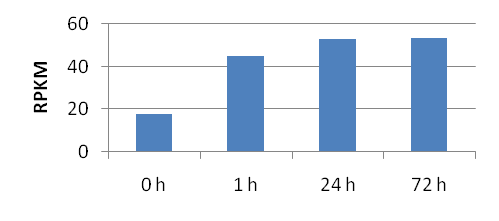 |

**Figure S4 Gene expression levels revealed by qRT-PCR (red column) and RNA-seq (blue column).** Data from qRT-PCR are means of three replicates and bars represent SE.
