# Supplementary material for: Transcriptomic Analysis of Drought Stress Responses in Ammopiptanthus mongolicus Leaves Using the RNA-Seq Technique
Source: PLoS One. 2015 Apr 29;10(4):e0124382. doi: 10.1371/journal.pone.0124382 (PMC4414462; doi:10.1371/journal.pone.0124382)
Supplement: S5 Fig — The up-regulated genes are boxed in red and the down-regulated genes are boxed in green. (A) Auxin, (B) Cytokinine, (C) Gibberellin, (D) Abscisic acid, (E) Ethylene, (F) Brassinosteroid, (G) Jasmonic acid, and (H) Salicylic acid. (DOCX) [file pone.0124382.s005.docx]

**Figure S5.** **KEGG enrichment analysis revealed that multiple hormones signal transduction pathways were involved in the drought response in *A. mongolicus* leaves.** The up-regulated genes were boxed in red and the down-regulated genes were boxed in green. (A) Auxin, (B) Cytokinine, (C) Gibberellin, (D) Abscisic acid, (E) Ethylene, (F) Brassinosteroid, (G) Jasmonic acid, (H) Salicylic acid.

**A**


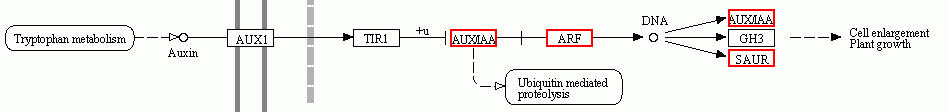


1 h


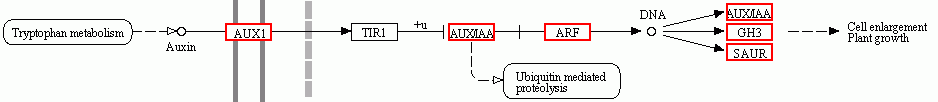


24 h


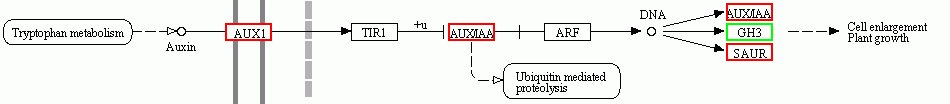


72 h

72 h

**B**


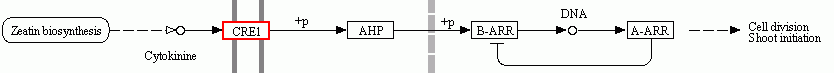


1 h


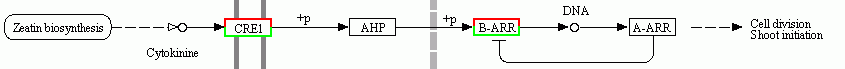


24 h


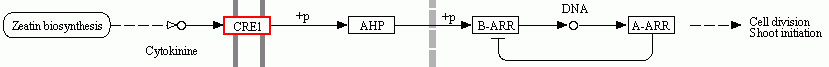


**C**


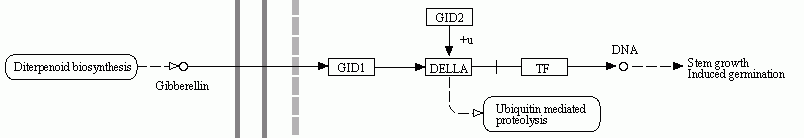


1 h


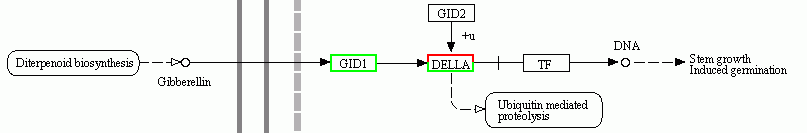


24 h


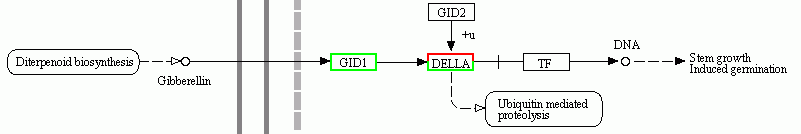


72 h

**D**

**
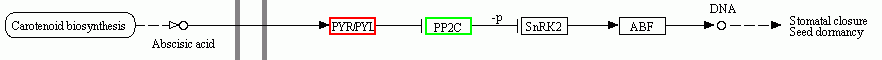
**

1 h

1 h

**
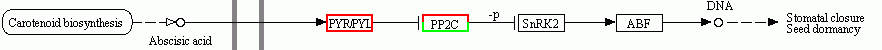
**

24 h

24 h

**
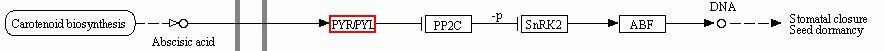
**

**E**

**
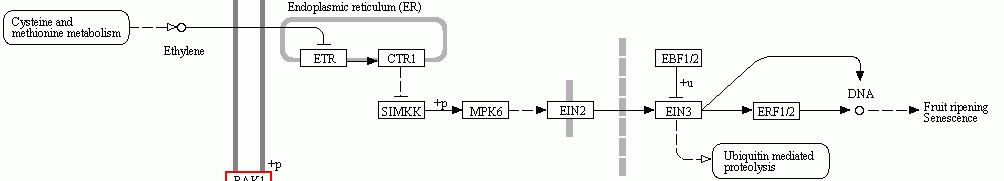
**

**
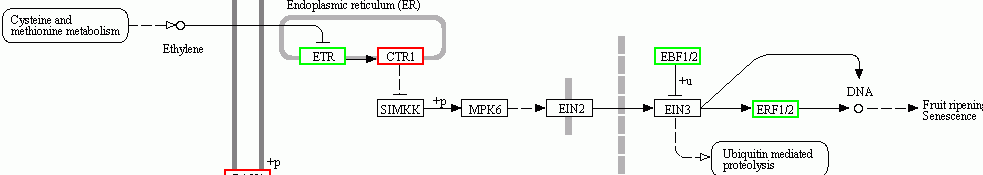
**

**
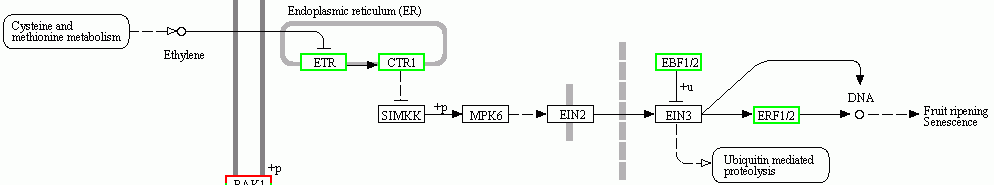
**

72 h

**F**

1 h

**
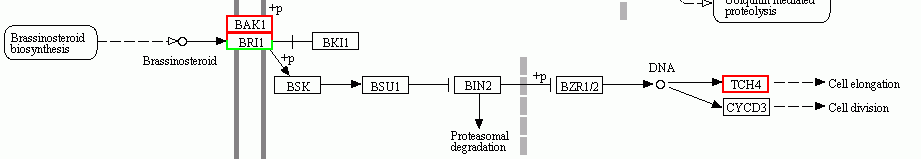
**

**
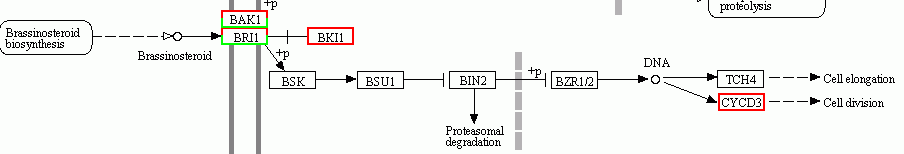
**

24 h

**
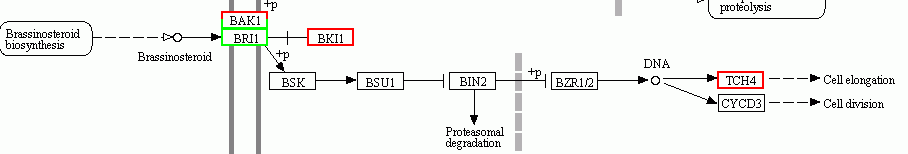
**

72 h

**G**

**
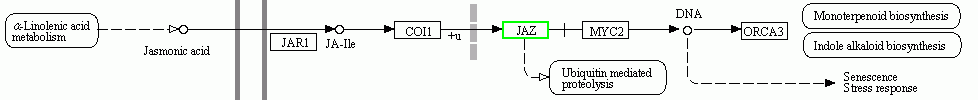
**

1 h

**
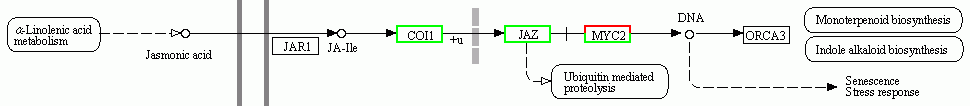
**

24 h

**
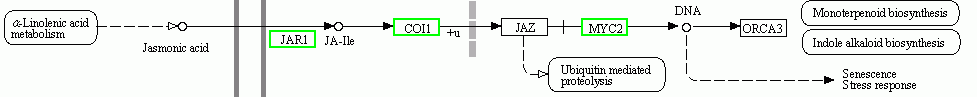
**

72 h

72 h

**H**

**
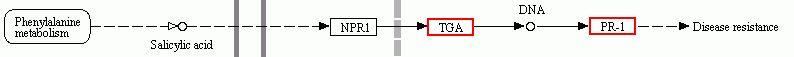
**

1 h

**
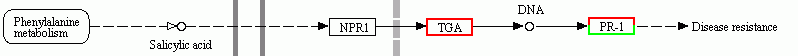
**

24 h

**
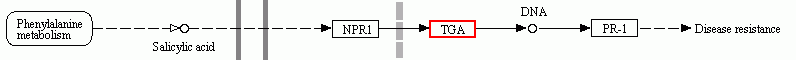
**
