# Supplementary material for: Transcriptomic Analysis of Drought Stress Responses in Ammopiptanthus mongolicus Leaves Using the RNA-Seq Technique
Source: PLoS One. 2015 Apr 29;10(4):e0124382. doi: 10.1371/journal.pone.0124382 (PMC4414462; doi:10.1371/journal.pone.0124382)
Supplement: S1 Table — (DOCX) [file pone.0124382.s006.docx]

**Table S1. Primer sequences used for qRT-PCR analysis.**

| Unigenes ID | Anotations /gene name | Foward primers | Reverse primers |
| --- | --- | --- | --- |
| CL2929.Contig1_All | nicotianamine synthase 3 | TCAACCTCAACCATGGGAAT | TCAAGCTTGCATGTGTGGAT |
| CL3605.Contig2_All | Peroxidase superfamily protein | TGGAAGAGGTGGAAGAAGTGA | ACACGCAGAGTCAATCCCTAA |
| CL6044.Contig2_All | alcohol dehydrogenase 1 | ACAAACCGCTCACTGACCTT | TTCTCCCAAAGCAGAGGCTA |
| Unigene15170_All | germin-like protein 1 | TGAAGACACTGTCAAAGGGAGA | AGGATTTGAAGGCCTGGATT |
| Unigene35165_All | beta galactosidase 1 | TTGTGTTGGACAGAACTGGTG | CTGAGGTTGAAATGATCAGGTG |
| Unigene2216_All | E3 ubiquitin-protein ligase RMA1H1-like isoform 1 | GTTATCATCCCCGTCCTCCT | GCCTTCTGATCCTTGGGTTAC |
| Unigene1196_All | expansin A4 | CGCACCTCTACCTCTTGGAA | ATGAAGGGGTGTCGTGAGTG |
| Unigene16704_All | plant U-box 26 | GCCGCTGTTGGTGAAGATAA | ACTCTGCACAAGAAGCAGCA |
| Unigene36201_All | lipid transfer protein 3 | GACACAGGGTGGAACTGCTT | GGTGGAGGTGCTGATTTTGT |
| - | 16S rRNA | GGCTCTGCCCGTTGCTCT | CGTCACCCGTCACCACCA |

Unigene33150_All

Unigene31532_All

| Unigene31282_All | 825 |
| --- | --- |
| CL2423.Contig2_All | 327 |
| Unigene17299_All | 614 |
| Unigene2580_All | 1897 |

**1** nicotianamine synthase

72h

| CL2929.Contig1_All | 1291 | 40 | 234 | 6.63002 | 39.06411 | 2.558759 | Up |
| --- | --- | --- | --- | --- | --- | --- | --- |

24h

| CL2929.Contig1_All | 1291 | 40 | 146 | 6.63002 | 26.29996 | 1.987976 | Up |
| --- | --- | --- | --- | --- | --- | --- | --- |

1h

| CL2929.Contig1_All | 1291 | 40 | 69 | 6.63002 | 12.41338 | 0.904811 | Up |
| --- | --- | --- | --- | --- | --- | --- | --- |

Encodes a nicotianamine synthase，[nicotianamine biosynthetic process](http://www.arabidopsis.org/servlets/TairObject?type=keyword&id=10324), [phloem transport](http://www.arabidopsis.org/servlets/TairObject?type=keyword&id=19800), [pollen development](http://www.arabidopsis.org/servlets/TairObject?type=keyword&id=11329), [pollen tube growth](http://www.arabidopsis.org/servlets/TairObject?type=keyword&id=10927)

>CL2929.Contig1_All size 1291 gap 0 0%

GGCTTGCAAGATGGGGACTCGATCTAAAACACATAATCGATCTCTTCTTTTATTTCTTCAAGATTTAAGAACAACATCACGAAAAACATACATAGTGCTAGATAGTGCACAAACGAGTACTCAGACATGGATGGAAATATTAACTATGCTGCTAAAGCTACTGCAAGACGTACATCATCCCTATGCATGTCAAGCTTGCATGTGTGGATCGGAGACACCAAATTGAAAATTAGGCTGTAGTCCGATCGAGAGTTTAGGATAGCTGCTCCTGATCAATGACCAATTCCTCAATCATATGCATATTCCCATGGTTGAGGTTGAGGGGGTTAAATACTTTGATCTCATTATTACAACAATTATAATTTGGTAGTATCGTAGAGCCAAGGCCTAGCTCAAGTGAGTGTGTAGGCATAGGATACTTGCGTGCTATGACAACTGAATTGATAACCTCATTCGTAGGGTGAAACACAGATAGGACCTCAAAACCTCGAAGATCGCAAGGATCAACCACCGGATAGAGAAAAGCCCGAGCAGCATGGGCACTTCTAAGCATGAGAAGGGCTCCTGGGGCCATGTACTTGGCCAAGTGATCAATGATTCGGTTCTTTTCCTCATTGTCCATGCCCACAAGTGCTGCCAAGTAAACAACTTCATACTCTTTCAAAGCATTTGACACGTCCAATATATCACTAGTGTGAAACACCATACGCTTTGACATGTTAGGGTCAGAGGAGACCAATTGCAGGGCTTTGGAATTGGCTAAAGGGTCGATGTCATAGTTGTGAAAAATGGTGGTAGGTAAGTGATTAGAGGCCAAGACAACTGAAGTCAAGGGAAGGGGACCTGAGCCCACAAAGGCAATTTTGGTAGGGATATGAGTGCAGTGCTGGCTGAGGATAGTGAATTCAAGGAGACCAAGCTTGATGTAATTGGAATAGTAAGGGAAAATATGGAGATGATGGAGTGGGTTCTCGTATGGAGCTAAGATGGATGAGTAGTGGCTCTCCATAAGGCCTTCAGCTTCACCACATAGCCTTATGAGCTTGGTTCTAATCTCTTGCACCCTTGTGCTCAGCTTAGTAACATCAATGGGGCTAGGTGGCATGCATGTGAGAACGAGTTGGGTGAAAAGGGTGTTGACATTTTTGCAGGGTTTGAGACTCTCTAGGCTTGAGATTTGTTCATACAGATCGCAGACTTTTTCAATCAACACCTCTTCCTGGCTAACCATGTCGACACTATGTTGTTATGGTAAGGAGCGTGAGACTTGGCTCGAGAAAACGGTACTGAG

**reverse complement:**

CTCAGTACCGTTTTCTCGAGCCAAGTCTCACGCTCCTTACCATAACAACATAGTGTCGACATGGTTAGCCAGGAAGAGGTGTTGATTGAAAAAGTCTGCGATCTGTATGAACAAATCTCAAGCCTAGAGAGTCTCAAACCCTGCAAAAATGTCAACACCCTTTTCACCCAACTCGTTCTCACATGCATGCCACCTAGCCCCATTGATGTTACTAAGCTGAGCACAAGGGTGCAAGAGATTAGAACCAAGCTCATAAGGCTATGTGGTGAAGCTGAAGGCCTTATGGAGAGCCACTACTCATCCATCTTAGCTCCATACGAGAACCCACTCCATCATCTCCATATTTTCCCTTACTATTCCAATTACATCAAGCTTGGTCTCCTTGAATTCACTATCCTCAGCCAGCACTGCACTCATATCCCTACCAAAATTGCCTTTGTGGGCTCAGGTCCCCTTCCCTTGACTTCAGTTGTCTTGGCCTCTAATCACTTACCTACCACCATTTTTCACAACTATGACATCGACCCTTTAGCCAATTCCAAAGCCCTGCAATTGGTCTCCTCTGACCCTAACATGTCAAAGCGTATGGTGTTTCACACTAGTGATATATTGGACGTGTCAAATGCTTTGAAAGAGTATGAAGTTGTTTACTTGGCAGCACTTGTGGGCATGGACAATGAGGAAAAGAACCGAATCATTGATCACTTGGCCAAGTACATGGCCCCAGGAGCCCTTCTCATGCTTAGAAGTGCCCATGCTGCTCGGGCTTTTCTCTATCCGGTGGTTGATCCTTGCGATCTTCGAGGTTTTGAGGTCCTATCTGTGTTTCACCCTACGAATGAGGTTATCAATTCAGTTGTCATAGCACGCAAGTATCCTATGCCTACACACTCACTTGAGCTAGGCCTTGGCTCTACGATACTACCAAATTATAATTGTTGTAATAATGAGATCAAAGTATTTAACCCCCTCAACCTCAACCATGGGAATATGCATATGATTGAGGAATTGGTCATTGATCAGGAGCAGCTATCCTAAACTCTCGATCGGACTACAGCCTAATTTTCAATTTGGTGTCTCCGATCCACACATGCAAGCTTGACATGCATAGGGATGATGTACGTCTTGCAGTAGCTTTAGCAGCATAGTTAATATTTCCATCCATGTCTGAGTACTCGTTTGTGCACTATCTAGCACTATGTATGTTTTTCGTGATGTTGTTCTTAAATCTTGAAGAAATAAAAGAAGAGATCGATTATGTGTTTTAGATCGAGTCCCCATCTTGCAAGCC

>**[AT1G09240](http://www.arabidopsis.org/servlets/TairObject?type=locus&name=AT1G09240).1** | Symbols: NAS3, ATNAS3 | nicotianamine synthase 3 |

chr1:2984950-2985912 FORWARD LENGTH=320

Length = 320

Plus Strand HSPs:

Score = 991 (353.9 bits), Expect = 3.9e-100, P = 3.9e-100

Identities = 197/328 (60%), Positives = 254/328 (77%), Frame = +1

Query: 61 MVSQEEVLIEKVCDLYEQISSLESLKPCKNVNTLFTQLVLTCMPPSP-IDVTKLSTRVQE 237

M Q+E L++ +CDLYE+IS LESLKP ++VN LF QLV TC+PP+P IDVTK+ RVQE

Sbjct: 1 MGCQDEQLVQTICDLYEKISKLESLKPSEDVNILFKQLVSTCIPPNPNIDVTKMCDRVQE 60

Query: 238 IRTKLIRLCGEAEGLMESHYSSILAPYE-NPLHHLHIFPYYSNYIKLGLLEFTILSQHCT 414

IR LI++CG AEG +E+H+SSIL Y+ NPLHHL+IFPYY+NY+KLG LEF +L Q+

Sbjct: 61 IRLNLIKICGLAEGHLENHFSSILTSYQDNPLHHLNIFPYYNNYLKLGKLEFDLLEQNLN 120

Query: 415 -HIPTKIAFVGSGPLPLTSVVLASNHLPTTIFHNYDIDPLANSKALQLVSSDPNMSKRMV 591

+P +AF+GSGPLPLTS+VLAS HL TIFHN+DIDP ANS A LVSSDP++S+RM

Sbjct: 121 GFVPKSVAFIGSGPLPLTSIVLASFHLKDTIFHNFDIDPSANSLASLLVSSDPDISQRMF 180

Query: 592 FHTSDILDVSNALKEYEVVYLAALVGMDNEEKNRIIDHLAKYMAPGALLMLRSAHAARAF 771

FHT DI+DV+ +LK ++VV+LAALVGM+ EEK ++I+HL K+MAPGA+LMLRSAH RAF

Sbjct: 181 FHTVDIMDVTESLKSFDVVFLAALVGMNKEEKVKVIEHLQKHMAPGAVLMLRSAHGPRAF 240

Query: 772 LYPVVDPCDLRGFEVLSVFHPTNEVINSVVIARKYPMPTHSLELGLGSTILPNYNCCNNE 951

LYP+V+PCDL+GFEVLS++HPT++VINSVVI++K+P+ + G S +L NC

Sbjct: 241 LYPIVEPCDLQGFEVLSIYHPTDDVINSVVISKKHPVVSIGNVGGPNSCLLKPCNCSKTH 300

Query: 952 IKVFNPLNLNHGNMHMIEELVIDQEQLS 1035

K+ + NM MIEE +EQLS

Sbjct: 301 AKM-------NKNM-MIEEFGAREEQLS 320

OLIGO [start](http://bioinfo.ut.ee/primer3-0.4.0/primer3_www_results_help.html#PRIMER_START)  [len](http://bioinfo.ut.ee/primer3-0.4.0/primer3_www_results_help.html#PRIMER_LEN)  [tm](http://bioinfo.ut.ee/primer3-0.4.0/primer3_www_results_help.html#PRIMER_TM)  [gc%](http://bioinfo.ut.ee/primer3-0.4.0/primer3_www_results_help.html#PRIMER_GC)  [any](http://bioinfo.ut.ee/primer3-0.4.0/primer3_www_results_help.html#PRIMER_ANY)  [3'](http://bioinfo.ut.ee/primer3-0.4.0/primer3_www_results_help.html#PRIMER_REPEAT) [seq](http://bioinfo.ut.ee/primer3-0.4.0/primer3_www_results_help.html#PRIMER_OLIGO_SEQ)

LEFT PRIMER 88 20 60.17 45.00 6.00 2.00 TCAACCTCAACCATGGGAAT

RIGHT PRIMER 219 20 60.27 45.00 8.00 2.00 TCAAGCTTGCATGTGTGGAT

SEQUENCE SIZE: 250

INCLUDED REGION SIZE: 250

PRODUCT SIZE: 132, PAIR ANY COMPL: 5.00, PAIR 3' COMPL: 2.00

1 CTACACACTCACTTGAGCTAGGCCTTGGCTCTACGATACTACCAAATTATAATTGTTGTA

61 ATAATGAGATCAAAGTATTTAACCCCCTCAACCTCAACCATGGGAATATGCATATGATTG

>>>>>>>>>>>>>>>>>>>>

121 AGGAATTGGTCATTGATCAGGAGCAGCTATCCTAAACTCTCGATCGGACTACAGCCTAAT

181 TTTCAATTTGGTGTCTCCGATCCACACATGCAAGCTTGACATGCATAGGGATGATGTACG

<<<<<<<<<<<<<<<<<<<<

241 TCTTGCAGTA

KEYS (in order of precedence):

>>>>>> left primer

<<<<<< right primer

ADDITIONAL OLIGOS

[start](http://bioinfo.ut.ee/primer3-0.4.0/primer3_www_results_help.html#PRIMER_START)  [len](http://bioinfo.ut.ee/primer3-0.4.0/primer3_www_results_help.html#PRIMER_LEN)  [tm](http://bioinfo.ut.ee/primer3-0.4.0/primer3_www_results_help.html#PRIMER_TM)  [gc%](http://bioinfo.ut.ee/primer3-0.4.0/primer3_www_results_help.html#PRIMER_GC)  [any](http://bioinfo.ut.ee/primer3-0.4.0/primer3_www_results_help.html#PRIMER_ANY)  [3'](http://bioinfo.ut.ee/primer3-0.4.0/primer3_www_results_help.html#PRIMER_REPEAT) [seq](http://bioinfo.ut.ee/primer3-0.4.0/primer3_www_results_help.html#PRIMER_OLIGO_SEQ)

1 LEFT PRIMER 88 20 60.17 45.00 6.00 2.00 TCAACCTCAACCATGGGAAT

RIGHT PRIMER 209 20 60.82 55.00 5.00 3.00 ATGTGTGGATCGGAGACACC

PRODUCT SIZE: 122, PAIR ANY COMPL: 4.00, PAIR 3' COMPL: 1.00

2 LEFT PRIMER 88 20 60.17 45.00 6.00 2.00 TCAACCTCAACCATGGGAAT

RIGHT PRIMER 220 20 60.88 50.00 8.00 2.00 GTCAAGCTTGCATGTGTGGA

PRODUCT SIZE: 133, PAIR ANY COMPL: 5.00, PAIR 3' COMPL: 3.00

3 LEFT PRIMER 78 21 60.21 47.62 4.00 0.00 TTTAACCCCCTCAACCTCAAC

RIGHT PRIMER 205 20 59.79 50.00 5.00 2.00 GTGGATCGGAGACACCAAAT

PRODUCT SIZE: 128, PAIR ANY COMPL: 3.00, PAIR 3' COMPL: 0.00

4 LEFT PRIMER 78 21 60.21 47.62 4.00 0.00 TTTAACCCCCTCAACCTCAAC

RIGHT PRIMER 219 20 60.27 45.00 8.00 2.00 TCAAGCTTGCATGTGTGGAT

PRODUCT SIZE: 142, PAIR ANY COMPL: 4.00, PAIR 3' COMPL: 2.00

**2 Peroxidase superfamily protein**

72h

| CL3605.Contig2_All | 1253 | 225 | 459 | 38.42488 | 78.94959 | 1.038891 | Up |
| --- | --- | --- | --- | --- | --- | --- | --- |

24h

| CL3605.Contig2_All | 1253 | 225 | 290 | 38.42488 | 53.82393 | 0.486207 | Up |
| --- | --- | --- | --- | --- | --- | --- | --- |

1h

| CL3605.Contig2_All | 1253 | 225 | 191 | 38.42488 | 35.40378 | -0.11814 | Down |
| --- | --- | --- | --- | --- | --- | --- | --- |

>CL3605.Contig2_All size 1253 gap 0 0%

GAAACATAAACTAGAAATATACTGCATAGCAAACTCAAATTCAGGGGTTCTTATTACACGCAGAGTCAATCCCTAAATAAGAAACAAAAGGCCTAAGTTAGAACGTCCATGTCACCACAAACATTTCAGAAAACTAAAAACCAAGAAAACATCACACACACACACATAAGTAATTGACTATGACTCAGTTTAAAACTGATCAATCACTTCTTCCACCTCTTCCACTACAGAGCTTAAGATAGATTGCTTCTTGTTTACCACATTGCATTTGGCACGAATTTCACCTTGATTTCGTGTCAAAACATCCAATTGGCTAATCCTTATCATAGCATTCACAAACTTCTCAAAAAACAAATTCTGGTCATTGGCGAAGGAATTTACTAATCCTTTAGTCTTATCATTATTGGTCAAGTCCTGGTCTGATGTGAACACACCCTGACGATTCAGTAGATCAATGTAGTACTTGTTATCAAAAACCGCTGGGGTTCTGATGTCCAAGTTAGCAGTGTTACCCGAGTTTGCATCTGGGCATGTAGCCTTAAGATTGTTGGCTAAGGTTTTGTCCATGGTGGGGTCTATAGTAGGGGAAAGTCTGTTGAAGAATGTGCCACAGTGGGCACGACCGAAAGTGTGTGCACCTGATAAGGCAACCACATCGGTCTCACTGAAGTTTCTGGCTGCAAAAGTTTTGATGTGTGTATTGGTGGTGTCAAAGGGTGATGGAAGATTATTTGTTCCTGCTATGTTGAAGTTTAGGCCATCCCTCCTTCCTAGTGGCACTGCAAAATCGGGGCCTCCAGAAAGAAAAACAGCATCACGAGCTGCCAATACAGTGATGTCGGCACAGGAGACGGTCCTTCCACACTTCTTGTGGACAAGACCTCGCAAGTCATCAATTGTCTGCAATGCCTCAGTCCTCATACCTCCATTGGCTGGTTGATCCCTTTCACCAGGTTTACCATCCAACAGCACCGACCCATCGCATCCCTGAACAAAGCAGTCATGGAAAAAGATACGGAGTAAGCCAGGCGCTTGGCCATTGTCCTGCTTGAACACCTTCTTGAGATGGTTTCTCACAATGTTTTCAAGATTGGGACAAGTTTTGGCAAAGAAAGAAAATGACAGTCCCTTCACTACTGGGGGTGGGGCATGTGCCTCGGAGACGTGGAAATGAGAGACCAACAGAAGGGAAGAAACTAAGAGCAAAGAGTACAAAGGAGAGTTACAGGCACTAATTACCCTAGCCATCTTTT

**reverse complement:**

AAAAGATGGCTAGGGTAATTAGTGCCTGTAACTCTCCTTTGTACTCTTTGCTCTTAGTTTCTTCCCTTCTGTTGGTCTCTCATTTCCACGTCTCCGAGGCACATGCCCCACCCCCAGTAGTGAAGGGACTGTCATTTTCTTTCTTTGCCAAAACTTGTCCCAATCTTGAAAACATTGTGAGAAACCATCTCAAGAAGGTGTTCAAGCAGGACAATGGCCAAGCGCCTGGCTTACTCCGTATCTTTTTCCATGACTGCTTTGTTCAGGGATGCGATGGGTCGGTGCTGTTGGATGGTAAACCTGGTGAAAGGGATCAACCAGCCAATGGAGGTATGAGGACTGAGGCATTGCAGACAATTGATGACTTGCGAGGTCTTGTCCACAAGAAGTGTGGAAGGACCGTCTCCTGTGCCGACATCACTGTATTGGCAGCTCGTGATGCTGTTTTTCTTTCTGGAGGCCCCGATTTTGCAGTGCCACTAGGAAGGAGGGATGGCCTAAACTTCAACATAGCAGGAACAAATAATCTTCCATCACCCTTTGACACCACCAATACACACATCAAAACTTTTGCAGCCAGAAACTTCAGTGAGACCGATGTGGTTGCCTTATCAGGTGCACACACTTTCGGTCGTGCCCACTGTGGCACATTCTTCAACAGACTTTCCCCTACTATAGACCCCACCATGGACAAAACCTTAGCCAACAATCTTAAGGCTACATGCCCAGATGCAAACTCGGGTAACACTGCTAACTTGGACATCAGAACCCCAGCGGTTTTTGATAACAAGTACTACATTGATCTACTGAATCGTCAGGGTGTGTTCACATCAGACCAGGACTTGACCAATAATGATAAGACTAAAGGATTAGTAAATTCCTTCGCCAATGACCAGAATTTGTTTTTTGAGAAGTTTGTGAATGCTATGATAAGGATTAGCCAATTGGATGTTTTGACACGAAATCAAGGTGAAATTCGTGCCAAATGCAATGTGGTAAACAAGAAGCAATCTATCTTAAGCTCTGTAGTGGAAGAGGTGGAAGAAGTGATTGATCAGTTTTAAACTGAGTCATAGTCAATTACTTATGTGTGTGTGTGTGATGTTTTCTTGGTTTTTAGTTTTCTGAAATGTTTGTGGTGACATGGACGTTCTAACTTAGGCCTTTTGTTTCTTATTTAGGGATTGACTCTGCGTGTAATAAGAACCCCTGAATTTGAGTTTGCTATGCAGTATATTTCTAGTTTATGTTTC

>**[AT1G71695](http://www.arabidopsis.org/servlets/TairObject?type=locus&name=AT1G71695).1** | Symbols: | Peroxidase superfamily protein |

chr1:26964359-26966557 FORWARD LENGTH=358

Length = 358

Plus Strand HSPs:

Score = 895 (320.1 bits), Expect = 5.9e-90, P = 5.9e-90

Identities = 180/308 (58%), Positives = 221/308 (71%), Frame = +3

Query: 114 PVVKGLSFSFFAKTCPNLENIVRNHLKKVFKQDNGQAPGLLRIFFHDCFVQGCDGSVLLD 293

P+VKGLS++F+ K CP +ENI+R LKKVFK+D G A +LRI FHDCFVQGC+ SVLL

Sbjct: 39 PIVKGLSWNFYQKACPKVENIIRKELKKVFKRDIGLAAAILRIHFHDCFVQGCEASVLLA 98

Query: 294 GK---PGERDQPANGGMRTEALQTIDDLRGLVHKKCGRTVSCADITVLAARDAVFLSGGP 464

G PGE+ N +R +A I++LR LV KKCG+ VSC+DI LAARD+V LSGGP

Sbjct: 99 GSASGPGEQSSIPNLTLRQQAFVVINNLRALVQKKCGQVVSCSDILALAARDSVVLSGGP 158

Query: 465 DFAVPLGRRDGLNFNIAGT--NNLPSPFDTTNTHIKTFAARNFSETDVVALSGAHTFGRA 638

D+AVPLGRRD L F T NNLP PF + I FA RN + TD+VALSG HT G A

Sbjct: 159 DYAVPLGRRDSLAFASQETTLNNLPPPFFNASQLIADFANRNLNITDLVALSGGHTIGIA 218

Query: 639 HCGTFFNRLSPTIDPTMDKTLANNLKATCPDANSGNTANLDIRTPAVFDNKYYIDLLNRQ 818

HC +F +RL P DPTM++ AN+LK TCP ANS NT DIR+P VFDNKYY+DL+NRQ

Sbjct: 219 HCPSFTDRLYPNQDPTMNQFFANSLKRTCPTANSSNTQVNDIRSPDVFDNKYYVDLMNRQ 278

Query: 819 GVFTSDQDLTNNDKTKGLVNSFANDQNLFFEKFVNAMIRISQLDVLTRNQGEIRAKCNVV 998

G+FTSDQDL + +T+G+V SFA DQ LFF+ F AMI++ Q+ VLT QGEIR+ C+

Sbjct: 279 GLFTSDQDLFVDKRTRGIVESFAIDQQLFFDYFTVAMIKMGQMSVLTGTQGEIRSNCSAR 338

Query: 999 NKKQSILS 1022

N QS +S

Sbjct: 339 NT-QSFMS 345

OLIGO [start](http://bioinfo.ut.ee/primer3-0.4.0/primer3_www_results_help.html#PRIMER_START)  [len](http://bioinfo.ut.ee/primer3-0.4.0/primer3_www_results_help.html#PRIMER_LEN)  [tm](http://bioinfo.ut.ee/primer3-0.4.0/primer3_www_results_help.html#PRIMER_TM)  [gc%](http://bioinfo.ut.ee/primer3-0.4.0/primer3_www_results_help.html#PRIMER_GC)  [any](http://bioinfo.ut.ee/primer3-0.4.0/primer3_www_results_help.html#PRIMER_ANY)  [3'](http://bioinfo.ut.ee/primer3-0.4.0/primer3_www_results_help.html#PRIMER_REPEAT) [seq](http://bioinfo.ut.ee/primer3-0.4.0/primer3_www_results_help.html#PRIMER_OLIGO_SEQ)

LEFT PRIMER 47 21 59.83 47.62 1.00 1.00 TGGAAGAGGTGGAAGAAGTGA

RIGHT PRIMER 215 21 59.75 47.62 3.00 1.00 ACACGCAGAGTCAATCCCTAA

SEQUENCE SIZE: 270

INCLUDED REGION SIZE: 270

PRODUCT SIZE: 169, PAIR ANY COMPL: 3.00, PAIR 3' COMPL: 1.00

1 AAATGCAATGTGGTAAACAAGAAGCAATCTATCTTAAGCTCTGTAGTGGAAGAGGTGGAA

>>>>>>>>>>>>>>

61 GAAGTGATTGATCAGTTTTAAACTGAGTCATAGTCAATTACTTATGTGTGTGTGTGTGAT

>>>>>>>

121 GTTTTCTTGGTTTTTAGTTTTCTGAAATGTTTGTGGTGACATGGACGTTCTAACTTAGGC

181 CTTTTGTTTCTTATTTAGGGATTGACTCTGCGTGTAATAAGAACCCCTGAATTTGAGTTT

<<<<<<<<<<<<<<<<<<<<<

241 GCTATGCAGTATATTTCTAGTTTATGTTTC

KEYS (in order of precedence):

>>>>>> left primer

<<<<<< right primer

ADDITIONAL OLIGOS

[start](http://bioinfo.ut.ee/primer3-0.4.0/primer3_www_results_help.html#PRIMER_START)  [len](http://bioinfo.ut.ee/primer3-0.4.0/primer3_www_results_help.html#PRIMER_LEN)  [tm](http://bioinfo.ut.ee/primer3-0.4.0/primer3_www_results_help.html#PRIMER_TM)  [gc%](http://bioinfo.ut.ee/primer3-0.4.0/primer3_www_results_help.html#PRIMER_GC)  [any](http://bioinfo.ut.ee/primer3-0.4.0/primer3_www_results_help.html#PRIMER_ANY)  [3'](http://bioinfo.ut.ee/primer3-0.4.0/primer3_www_results_help.html#PRIMER_REPEAT) [seq](http://bioinfo.ut.ee/primer3-0.4.0/primer3_www_results_help.html#PRIMER_OLIGO_SEQ)

1 LEFT PRIMER 47 21 59.83 47.62 1.00 1.00 TGGAAGAGGTGGAAGAAGTGA

RIGHT PRIMER 214 21 60.26 47.62 3.00 0.00 CACGCAGAGTCAATCCCTAAA

PRODUCT SIZE: 168, PAIR ANY COMPL: 3.00, PAIR 3' COMPL: 2.00

2 LEFT PRIMER 47 21 59.83 47.62 1.00 1.00 TGGAAGAGGTGGAAGAAGTGA

RIGHT PRIMER 228 22 60.02 45.45 3.00 0.00 AGGGGTTCTTATTACACGCAGA

PRODUCT SIZE: 182, PAIR ANY COMPL: 6.00, PAIR 3' COMPL: 0.00

3 LEFT PRIMER 47 21 59.83 47.62 1.00 1.00 TGGAAGAGGTGGAAGAAGTGA

RIGHT PRIMER 227 22 60.02 50.00 3.00 1.00 GGGGTTCTTATTACACGCAGAG

PRODUCT SIZE: 181, PAIR ANY COMPL: 6.00, PAIR 3' COMPL: 0.00

4 LEFT PRIMER 47 22 60.11 45.45 2.00 2.00 TGGAAGAGGTGGAAGAAGTGAT

RIGHT PRIMER 215 21 59.75 47.62 3.00 1.00 ACACGCAGAGTCAATCCCTAA

PRODUCT SIZE: 169, PAIR ANY COMPL: 4.00, PAIR 3' COMPL: 2.00

**3 alcohol dehydrogenase 1**

72

| CL6044.Contig2_All | 1800 | 3186 | 7571 | 378.7515 | 906.5026 | 1.25906 | Up |
| --- | --- | --- | --- | --- | --- | --- | --- |

24

| CL6044.Contig2_All | 1800 | 3186 | 10287 | 378.7515 | 1329.06 | 1.811083 | Up |
| --- | --- | --- | --- | --- | --- | --- | --- |

1

| CL6044.Contig2_All | 1800 | 3186 | 4840 | 378.7515 | 624.5111 | 0.721476 | Up |
| --- | --- | --- | --- | --- | --- | --- | --- |

>CL6044.Contig2_All size 1800 gap 0 0%

GCTATTCTACTAACTGGGCCCACATCCAGACCCGCATGACCTTGCATCTTTATCCATATAAAACCCCTGTGCTTTCTCACTCGCCAGCCCACCAGCCAAATTGAAGCTCTTTCAGCCAGCAACGAGCTTCAAAACACTGAAATTTGAACGTGCTGCTCATATAGCCTTCCCTTTTTCAATCCTTCTCTTCTCCAGAATTCAAGACACCAGAACCTTCTGCTTCTGCTCGTTTTCCCTCTCACCTTGCTTCTTATACACCCTGTTGTGTGAGCTCTTCTTCCACTCTCACTATCACATTAATTGAAACAGGTATTCGTCGTCGGATCCATCATATATATTTCATATATATCCTCCTCCTTCGTTAGGAGCCATGCCTAGCAAAGCTGGTGAGGTCATCAAGTGCAAAGCTGCGGTGGCATGGGAAGCAGGGAAGCCACTAGTGATTGAAGAAGTAGAGGTAGCGCCACCACAGGCTGGTGAAGTTCGTTTGAAGATCCTCTACACCTCTCTTTGTCGTTCTGATGTTTACTTCTGGGACGCCAAGGGCCAGAGTTTGTTGTTTCCCCGTATACTTGGTCACGAAGCTGCAGGGATTGTGGAGAGCGTAGGTGAGGGTGTGACTCATCTCAAACCAGGAGACCATGCCCTCCCTGTATTTACTGGAGAGTGTGGGGAATGCCCACATTGTAAGTCAGAAGAGAGCAACTTATGTGACCTACTCAGGGTCAACACTGATAGGGGTGTTATGCTCAGTGATGGTCAATCAAGATTCTCCAAAAATGGACAAACCATATACCACTTTCTGGGAACCTCTACATTTAGTGAATACACTGTTTCCCATGCTAAATGTGTTGCAAAGATCAACCCTGCTGCTCCACTTGACAAAGTTTGTATTCTCAGTTGTGGATTCTGCACAGGACTTGGTGCTACTATAAACGTGGCCAAACCGAAGCCTGGTTCTTCTGTGGCTATCTTTGGATTGGGAGCTGTTGGCCTTGCTGCTGCTGAAGGAGCAAGGCTTTCTGGTGCATCAAGAATCATTGGGGTTGATATAATTTCCAGCAGATTCGAACAAGCCAAAAATTTTGGAGTCACTGACTTTGTGAACCCAAAAGATCATGACAAACCTGTACAGCAGGTAATTGTTGAAATGACTAATGGAGGAGTTGATCGTGCTGTTGAATGCACTGGAAATATTGAAGCCTCCATAGCAGCATTTGAATGTGTTCACGATGGTTGGGGTGTTGCTGTACTTGTTGGTCTGCCAAAAAATGATGATGTATTCAAAACTCATGC**TATCAATTTCTTGTTTGAGAGGACAATTAAGGGAACCTTCTATGGAAACTACAAACCGCTCACTGA****CCTTCCATCTGTTGTAGAGAAGTACATGAACAAGGAGCTAGAAGTGGAGAAATTCATCACTCACTCGGTCCCATTTTCTGAGATTAACAAATCATTTGAATACATGCTGAAAGGGGAATCAATCAGGTGCATCATCCGAATGGAAGGGTAAAAACATAATGGGATAGCCTCTGCTTTGGGAGAATTATGTTTCTGCTTTTTGAATCAATAAAAGTCTTGAGAAATGAGAAATAAAAGTGCCTTTCTTGGTTGTCTGGA**TTGTGCATTAGTGACTTGTGTACAACTTAAGCAGTATCAGTGTTGAGAATTTATAGTTCATTTCTTGGTTTTCACATGTTTTTTCTTTGCTGTTTAGGTTGTGGCTTTTGTGACTTGTATCGAATTTAAGCAGTATATAAATAATATGAGTATTGAGGAGTTATAGTTCAAAAAAAAAAA

>**[AT1G77120](http://www.arabidopsis.org/servlets/TairObject?type=locus&name=AT1G77120).1** | Symbols: ADH1, ADH, ATADH, ATADH1 | alcohol dehydrogenase 1 |

chr1:28975509-28977216 FORWARD LENGTH=379

Length = 379

Plus Strand HSPs:

Score = 1523 (541.2 bits), Expect = 1.7e-156, P = 1.7e-156

Identities = 281/376 (74%), Positives = 318/376 (84%), Frame = +2

Query: 377 SKAGEVIKCKAAVAWEAGKPLVIEEVEVAPPQAGEVRLKILYTSLCRSDVYFWDAKGQSL 556

S G++I+CKAAVAWEAGKPLVIEEVEVAPPQ EVR+KIL+TSLC +DVYFW+AKGQ+

Sbjct: 2 STTGQIIRCKAAVAWEAGKPLVIEEVEVAPPQKHEVRIKILFTSLCHTDVYFWEAKGQTP 61

Query: 557 LFPRILGHEAAGIVESVGEGVTHLKPGDHALPVFTGECGECPHCKSEESNLCDLLRVNTD 736

LFPRI GHEA GIVESVGEGVT L+PGDH LP+FTGECGEC HC SEESN+CDLLR+NT+

Sbjct: 62 LFPRIFGHEAGGIVESVGEGVTDLQPGDHVLPIFTGECGECRHCHSEESNMCDLLRINTE 121

Query: 737 RGVMLSDGQSRFSKNGQTIYHFLGTSTFSEYTVSHAKCVAKINPAAPLDKVCILSCGFCT 916

RG M+ DG+SRFS NG+ IYHFLGTSTFSEYTV H+ VAKINP APLDKVCI+SCG T

Sbjct: 122 RGGMIHDGESRFSINGKPIYHFLGTSTFSEYTVVHSGQVAKINPDAPLDKVCIVSCGLST 181

Query: 917 GLGATINVAKPKPGSSVXXXXXXXXXXXXXXXXXXSGASRIIGVDIISSRFEQAKNFGVT 1096

GLGAT+NVAKPK G SV +GASRIIGVD S RF+QAK FGVT

Sbjct: 182 GLGATLNVAKPKKGQSVAIFGLGAVGLGAAEGARIAGASRIIGVDFNSKRFDQAKEFGVT 241

Query: 1097 DFVNPKDHDKPVQQVIVEMTNGGVDRAVECTGNIEASIAAFECVHDGWGVAVLVGLPKND 1276

+ VNPKDHDKP+QQVI EMT+GGVDR+VECTG+++A I AFECVHDGWGVAVLVG+P D

Sbjct: 242 ECVNPKDHDKPIQQVIAEMTDGGVDRSVECTGSVQAMIQAFECVHDGWGVAVLVGVPSKD 301

Query: 1277 DVFKTHAINFLFERTIKGTFYGNYKPLTDLPSVVEKYMNKELEVEKFITHSVPFSEINKS 1456

D FKTH +NFL ERT+KGTF+GNYKP TD+P VVEKYMNKELE+EKFITH+VPFSEINK+

Sbjct: 302 DAFKTHPMNFLNERTLKGTFFGNYKPKTDIPGVVEKYMNKELELEKFITHTVPFSEINKA 361

Query: 1457 FEYMLKGESIRCIIRM 1504

F+YMLKGESIRCII M

Sbjct: 362 FDYMLKGESIRCIITM 377

OLIGO [start](http://bioinfo.ut.ee/primer3-0.4.0/primer3_www_results_help.html#PRIMER_START)  [len](http://bioinfo.ut.ee/primer3-0.4.0/primer3_www_results_help.html#PRIMER_LEN)  [tm](http://bioinfo.ut.ee/primer3-0.4.0/primer3_www_results_help.html#PRIMER_TM)  [gc%](http://bioinfo.ut.ee/primer3-0.4.0/primer3_www_results_help.html#PRIMER_GC)  [any](http://bioinfo.ut.ee/primer3-0.4.0/primer3_www_results_help.html#PRIMER_ANY)  [3'](http://bioinfo.ut.ee/primer3-0.4.0/primer3_www_results_help.html#PRIMER_REPEAT) [seq](http://bioinfo.ut.ee/primer3-0.4.0/primer3_www_results_help.html#PRIMER_OLIGO_SEQ)

LEFT PRIMER 106 20 60.01 45.00 3.00 1.00 GCTGAAAGGGGAATCAATCA

RIGHT PRIMER 266 20 59.97 45.00 4.00 0.00 ATGCACAATCCAGACAACCA

SEQUENCE SIZE: 270

INCLUDED REGION SIZE: 270

PRODUCT SIZE: 161, PAIR ANY COMPL: 3.00, PAIR 3' COMPL: 0.00

1 CCTTCCATCTGTTGTAGAGAAGTACATGAACAAGGAGCTAGAAGTGGAGAAATTCATCAC

61 TCACTCGGTCCCATTTTCTGAGATTAACAAATCATTTGAATACATGCTGAAAGGGGAATC

>>>>>>>>>>>>>>>

121 AATCAGGTGCATCATCCGAATGGAAGGGTAAAAACATAATGGGATAGCCTCTGCTTTGGG

>>>>>

181 AGAATTATGTTTCTGCTTTTTGAATCAATAAAAGTCTTGAGAAATGAGAAATAAAAGTGC

241 CTTTCTTGGTTGTCTGGATTGTGCATTAGT

<<<<<<<<<<<<<<<<<<<<

KEYS (in order of precedence):

>>>>>> left primer

<<<<<< right primer

ADDITIONAL OLIGOS

[start](http://bioinfo.ut.ee/primer3-0.4.0/primer3_www_results_help.html#PRIMER_START)  [len](http://bioinfo.ut.ee/primer3-0.4.0/primer3_www_results_help.html#PRIMER_LEN)  [tm](http://bioinfo.ut.ee/primer3-0.4.0/primer3_www_results_help.html#PRIMER_TM)  [gc%](http://bioinfo.ut.ee/primer3-0.4.0/primer3_www_results_help.html#PRIMER_GC)  [any](http://bioinfo.ut.ee/primer3-0.4.0/primer3_www_results_help.html#PRIMER_ANY)  [3'](http://bioinfo.ut.ee/primer3-0.4.0/primer3_www_results_help.html#PRIMER_REPEAT) [seq](http://bioinfo.ut.ee/primer3-0.4.0/primer3_www_results_help.html#PRIMER_OLIGO_SEQ)

1 LEFT PRIMER 105 20 60.01 45.00 3.00 3.00 TGCTGAAAGGGGAATCAATC

RIGHT PRIMER 266 20 59.97 45.00 4.00 0.00 ATGCACAATCCAGACAACCA

PRODUCT SIZE: 162, PAIR ANY COMPL: 3.00, PAIR 3' COMPL: 0.00

**2 LEFT PRIMER 58 20 59.97 50.00 3.00 0.00 CACTCACTCGGTCCCATTTT**

**RIGHT PRIMER 266 20 59.97 45.00 4.00 0.00 ATGCACAATCCAGACAACCA**

**PRODUCT SIZE: 209, PAIR ANY COMPL: 3.00, PAIR 3' COMPL: 1.00**

3 LEFT PRIMER 108 20 59.86 45.00 3.00 0.00 TGAAAGGGGAATCAATCAGG

RIGHT PRIMER 266 20 59.97 45.00 4.00 0.00 ATGCACAATCCAGACAACCA

PRODUCT SIZE: 159, PAIR ANY COMPL: 3.00, PAIR 3' COMPL: 1.00

4 LEFT PRIMER 102 20 60.46 45.00 4.00 1.00 ACATGCTGAAAGGGGAATCA

RIGHT PRIMER 266 20 59.97 45.00 4.00 0.00 ATGCACAATCCAGACAACCA

PRODUCT SIZE: 165, PAIR ANY COMPL: 3.00, PAIR 3' COMPL: 0.00

避免连续4个：

OLIGO [start](http://bioinfo.ut.ee/primer3-0.4.0/primer3_www_results_help.html#PRIMER_START)  [len](http://bioinfo.ut.ee/primer3-0.4.0/primer3_www_results_help.html#PRIMER_LEN)  [tm](http://bioinfo.ut.ee/primer3-0.4.0/primer3_www_results_help.html#PRIMER_TM)  [gc%](http://bioinfo.ut.ee/primer3-0.4.0/primer3_www_results_help.html#PRIMER_GC)  [any](http://bioinfo.ut.ee/primer3-0.4.0/primer3_www_results_help.html#PRIMER_ANY)  [3'](http://bioinfo.ut.ee/primer3-0.4.0/primer3_www_results_help.html#PRIMER_REPEAT) [seq](http://bioinfo.ut.ee/primer3-0.4.0/primer3_www_results_help.html#PRIMER_OLIGO_SEQ)

LEFT PRIMER 124 20 59.97 50.00 3.00 0.00 CACTCACTCGGTCCCATTTT

RIGHT PRIMER 250 20 60.09 50.00 3.00 2.00 TTCTCCCAAAGCAGAGGCTA

SEQUENCE SIZE: 324

INCLUDED REGION SIZE: 324

PRODUCT SIZE: 127, PAIR ANY COMPL: 3.00, PAIR 3' COMPL: 1.00

1 TATCAATTTCTTGTTTGAGAGGACAATTAAGGGAACCTTCTATGGAAACTACAAACCGCT

61 CACTGACCTTCCATCTGTTGTAGAGAAGTACATGAACAAGGAGCTAGAAGTGGAGAAATT

121 CATCACTCACTCGGTCCCATTTTCTGAGATTAACAAATCATTTGAATACATGCTGAAAGG

>>>>>>>>>>>>>>>>>>>>

181 GGAATCAATCAGGTGCATCATCCGAATGGAAGGGTAAAAACATAATGGGATAGCCTCTGC

<<<<<<<<<<

241 TTTGGGAGAATTATGTTTCTGCTTTTTGAATCAATAAAAGTCTTGAGAAATGAGAAATAA

<<<<<<<<<<

301 AAGTGCCTTTCTTGGTTGTCTGGA

KEYS (in order of precedence):

>>>>>> left primer

<<<<<< right primer

ADDITIONAL OLIGOS

[start](http://bioinfo.ut.ee/primer3-0.4.0/primer3_www_results_help.html#PRIMER_START)  [len](http://bioinfo.ut.ee/primer3-0.4.0/primer3_www_results_help.html#PRIMER_LEN)  [tm](http://bioinfo.ut.ee/primer3-0.4.0/primer3_www_results_help.html#PRIMER_TM)  [gc%](http://bioinfo.ut.ee/primer3-0.4.0/primer3_www_results_help.html#PRIMER_GC)  [any](http://bioinfo.ut.ee/primer3-0.4.0/primer3_www_results_help.html#PRIMER_ANY)  [3'](http://bioinfo.ut.ee/primer3-0.4.0/primer3_www_results_help.html#PRIMER_REPEAT) [seq](http://bioinfo.ut.ee/primer3-0.4.0/primer3_www_results_help.html#PRIMER_OLIGO_SEQ)

1 LEFT PRIMER 51 20 59.77 50.00 5.00 1.00 ACAAACCGCTCACTGACCTT

RIGHT PRIMER 190 20 60.01 45.00 3.00 1.00 GATTGATTCCCCTTTCAGCA

PRODUCT SIZE: 140, PAIR ANY COMPL: 4.00, PAIR 3' COMPL: 1.00

2 LEFT PRIMER 51 20 59.77 50.00 5.00 1.00 ACAAACCGCTCACTGACCTT

RIGHT PRIMER 191 20 60.01 45.00 3.00 2.00 TGATTGATTCCCCTTTCAGC

PRODUCT SIZE: 141, PAIR ANY COMPL: 4.00, PAIR 3' COMPL: 0.00

**3 LEFT PRIMER 51 20 59.77 50.00 5.00 1.00 ACAAACCGCTCACTGACCTT**

**RIGHT PRIMER 250 20 60.09 50.00 3.00 2.00 TTCTCCCAAAGCAGAGGCTA**

**PRODUCT SIZE: 200, PAIR ANY COMPL: 3.00, PAIR 3' COMPL: 1.00**

4 LEFT PRIMER 51 20 59.77 50.00 5.00 1.00 ACAAACCGCTCACTGACCTT

RIGHT PRIMER 193 20 59.86 45.00 3.00 1.00 CCTGATTGATTCCCCTTTCA

PRODUCT SIZE: 143, PAIR ANY COMPL: 3.00, PAIR 3' COMPL: 1.00

**4 germin-like protein 1**

**72**

| Unigene15170_All | 855 | 900 | 2257 | 225.2462 | 568.9233 | 1.336731 | Up |
| --- | --- | --- | --- | --- | --- | --- | --- |

**24**

| Unigene15170_All | 855 | 900 | 2626 | 225.2462 | 714.2613 | 1.664949 | Up |
| --- | --- | --- | --- | --- | --- | --- | --- |

**1**

| Unigene15170_All | 855 | 900 | 2521 | 225.2462 | 684.8163 | 1.604214 | Up |
| --- | --- | --- | --- | --- | --- | --- | --- |

>Unigene15170_All size 855 gap 0 0%

AAGAACACCCTTAAGCTTCTTCACTATTTCAGGATCAAGGAAAGTAGTCTGTGTCACTAAAGCAGAAGAGAAGTTGCTGGCAAACAGTGCAAAATCAAGGATTTGAAGGCCTGGATTTGAGCTACTGAAAACAGCAAAAGCAACGGCCTTTCTCTTACCATAAGCTACTTGAAAATGCAACAATCCTTGTGGCAAAATGAAAATCTCTCCCTTTGACAGTGTCTTCACATAAACCGAGTTATCTGATGCTATAAATCCAGCAGTGATAAGACCTTGCTTCACTATTAGTAGTTCAGAAGCACCAGGATGAGTGTGCAGTGGGATTACACCACCAGGAGCAAGGTCTAACCGTGCTGCAGAAAGACCAAGTCCATTAACACCCGGAAATTGAGCAACAAATGCTGGGGTTACAGCTGCATTGATTATATTTGAGGTGTTTCCTTCCATTGATAACCCAGAAAATACAAAGTCAAGTGTAGTTACTTTTGTCGGAGGCTTGCAGGGAAAGCCTGCAGGGCCATCTGGACCCTTGAGGTCTGCTACACAAAAGTCATTGACAGAAGCCTGGGAAGTGGATAAGAGAAGAGGAAGGAGGAAAAGAATGTAAAGCATTTTGGTGGTAGATTTGGTTGCAATAGGAATGTGGGATTGGCTAGCTACATAATGCTACCATATATAGAGCTTTTCTAGCAAGTTGGAGCCATTGACAGGAAAAGGATATTTGCATTATAAAATTTTGCATATTACTCTTATATTAATGACTAACATGTAAATAATTAACTGCTAATCATTAAAGATGGCAATCAATTACTATTATATGATGATCAATGTATTATACAGTGATCAATGTAAAAG

CTTTTACATTGATCACTGTATAATACATTGATCATCATATAATAGTAATTGATTGCCATCTTTAATGATTAGCAGTTAATTATTTACATGTTAGTCATTAATATAAGAGTAATATGCAAAATTTTATAATGCAAATATCCTTTTCCTGTCAATGGCTCCAACTTGCTAGAAAAGCTCTATATATGGTAGCATTATGTAGCTAGCCAATCCCACATTCCTATTGCAACCAAATCTACCACCAAAATGCTTTACATTCTTTTCCTCCTTCCTCTTCTCTTATCCACTTCCCAGGCTTCTGTCAATGACTTTTGTGTAGCAGACCTCAAGGGTCCAGATGGCCCTGCAGGCTTTCCCTGCAAGCCTCCGACAAAAGTAACTACACTTGACTTTGTATTTTCTGGGTTATCAATGGAAGGAAACACCTCAAATATAATCAATGCAGCTGTAACCCCAGCATTTGTTGCTCAATTTCCGGGTGTTAATGGACTTGGTCTTTCTGCAGCACGGTTAGACCTTGCTCCTGGTGGTGTAATCCCACTGCACACTCATCCTGGTGCTTCTGAACTACTAATAGTGAAGCAAGGTCTTATCACTGCTGGATTTATAGCATCAGATAACTCGGTTTATGTGAAGACACTGTCAAAGGGAGAGATTTTCATTTTGCCACAAGGATTGTTGCATTTTCAAGTAGCTTATGGTAAGAGAAAGGCCGTTGCTTTTGCTGTTTTCAGTAGCTCAAATCCAGGCCTTCAAATCCTTGATTTTGCACTGTTTGCCAGCAACTTCTCTTCTGCTTTAGTGACACAGACTACTTTCCTTGATCCTGAAATAGTGAAGAAGCTTAAGGGTGTTCTT

[**AT1G72610**](http://www.arabidopsis.org/servlets/TairObject?type=locus&name=AT1G72610)**.1** | Symbols: GLP1, ATGER1, GER1 | germin-like protein 1 |

chr1:27339302-27339928 REVERSE LENGTH=208

Length = 208

Plus Strand HSPs:

Score = 647 (232.8 bits), Expect = 1.0e-63, P = 1.0e-63

Identities = 127/190 (66%), Positives = 147/190 (77%), Frame = +1

Query: 286 SQASVNDFCVADLKGPDGPAGFPCKPPTKVTTLDFVFSGLSMEGNTSNIINAAVTPAFVA 465

S ASV DFCVA+LK + PAG+PC P V DFVFSGL GNT+NIINAAVTPAF A

Sbjct: 15 SNASVQDFCVANLKRAETPAGYPCIRPIHVKATDFVFSGLGTPGNTTNIINAAVTPAFAA 74

Query: 466 QFPGVNGLGLSAARLDLAPGGVIPLHTHPGASELLIVKQGLITAGFIASDNSVYVKTLSK 645

QFPG+NGLGLS ARLDLAP GVIP+HTHPGASE+L V G ITAGF++S N+VYV+TL

Sbjct: 75 QFPGLNGLGLSTARLDLAPKGVIPMHTHPGASEVLFVLTGSITAGFVSSANAVYVQTLKP 134

Query: 646 GEIFILPQGLLHFQVAYGKRKAVAFAVFSSSNPGLQILDFALFASNFSSALVTQTTFLDP 825

G++ + PQGLLHFQ+ GK A A F+S+NPGLQILDFALFA++ + LV TTFLD

Sbjct: 135 GQVMVFPQGLLHFQINAGKSSASAVVTFNSANPGLQILDFALFANSLPTELVVGTTFLDA 194

Query: 826 EIVKKLKGVL 855

VKKLKGVL

Sbjct: 195 TTVKKLKGVL 204

OLIGO [start](http://bioinfo.ut.ee/primer3-0.4.0/primer3_www_results_help.html#PRIMER_START)  [len](http://bioinfo.ut.ee/primer3-0.4.0/primer3_www_results_help.html#PRIMER_LEN)  [tm](http://bioinfo.ut.ee/primer3-0.4.0/primer3_www_results_help.html#PRIMER_TM)  [gc%](http://bioinfo.ut.ee/primer3-0.4.0/primer3_www_results_help.html#PRIMER_GC)  [any](http://bioinfo.ut.ee/primer3-0.4.0/primer3_www_results_help.html#PRIMER_ANY)  [3'](http://bioinfo.ut.ee/primer3-0.4.0/primer3_www_results_help.html#PRIMER_REPEAT) [seq](http://bioinfo.ut.ee/primer3-0.4.0/primer3_www_results_help.html#PRIMER_OLIGO_SEQ)

LEFT PRIMER 30 22 59.88 45.45 7.00 0.00 TGAAGACACTGTCAAAGGGAGA

RIGHT PRIMER 159 20 59.90 45.00 6.00 1.00 AGGATTTGAAGGCCTGGATT

SEQUENCE SIZE: 256

INCLUDED REGION SIZE: 256

PRODUCT SIZE: 130, PAIR ANY COMPL: 5.00, PAIR 3' COMPL: 1.00

1 ATTTATAGCATCAGATAACTCGGTTTATGTGAAGACACTGTCAAAGGGAGAGATTTTCAT

>>>>>>>>>>>>>>>>>>>>>>

61 TTTGCCACAAGGATTGTTGCATTTTCAAGTAGCTTATGGTAAGAGAAAGGCCGTTGCTTT

121 TGCTGTTTTCAGTAGCTCAAATCCAGGCCTTCAAATCCTTGATTTTGCACTGTTTGCCAG

<<<<<<<<<<<<<<<<<<<<

181 CAACTTCTCTTCTGCTTTAGTGACACAGACTACTTTCCTTGATCCTGAAATAGTGAAGAA

241 GCTTAAGGGTGTTCTT

KEYS (in order of precedence):

>>>>>> left primer

<<<<<< right primer

ADDITIONAL OLIGOS

[start](http://bioinfo.ut.ee/primer3-0.4.0/primer3_www_results_help.html#PRIMER_START)  [len](http://bioinfo.ut.ee/primer3-0.4.0/primer3_www_results_help.html#PRIMER_LEN)  [tm](http://bioinfo.ut.ee/primer3-0.4.0/primer3_www_results_help.html#PRIMER_TM)  [gc%](http://bioinfo.ut.ee/primer3-0.4.0/primer3_www_results_help.html#PRIMER_GC)  [any](http://bioinfo.ut.ee/primer3-0.4.0/primer3_www_results_help.html#PRIMER_ANY)  [3'](http://bioinfo.ut.ee/primer3-0.4.0/primer3_www_results_help.html#PRIMER_REPEAT) [seq](http://bioinfo.ut.ee/primer3-0.4.0/primer3_www_results_help.html#PRIMER_OLIGO_SEQ)

1 LEFT PRIMER 30 22 59.88 45.45 7.00 0.00 TGAAGACACTGTCAAAGGGAGA

RIGHT PRIMER 160 20 59.90 45.00 6.00 2.00 AAGGATTTGAAGGCCTGGAT

PRODUCT SIZE: 131, PAIR ANY COMPL: 5.00, PAIR 3' COMPL: 1.00

2 LEFT PRIMER 64 20 60.90 45.00 7.00 1.00 GCCACAAGGATTGTTGCATT

RIGHT PRIMER 190 21 59.67 47.62 6.00 2.00 AGAGAAGTTGCTGGCAAACAG

PRODUCT SIZE: 127, PAIR ANY COMPL: 5.00, PAIR 3' COMPL: 0.00

3 LEFT PRIMER 30 22 59.88 45.45 7.00 0.00 TGAAGACACTGTCAAAGGGAGA

RIGHT PRIMER 149 20 59.84 55.00 4.00 1.00 GGCCTGGATTTGAGCTACTG

PRODUCT SIZE: 120, PAIR ANY COMPL: 5.00, PAIR 3' COMPL: 2.00

4 LEFT PRIMER 30 22 59.88 45.45 7.00 0.00 TGAAGACACTGTCAAAGGGAGA

RIGHT PRIMER 155 20 60.18 45.00 6.00 1.00 TTTGAAGGCCTGGATTTGAG

PRODUCT SIZE: 126, PAIR ANY COMPL: 5.00, PAIR 3' COMPL: 2.00

**5 beta galactosidase 1**

**72**

| Unigene35165_All | 3231 | 170 | 1089 | 11.25882 | 72.64056 | 2.689719 | Up |
| --- | --- | --- | --- | --- | --- | --- | --- |

**24**

| Unigene35165_All | 3231 | 170 | 3332 | 11.25882 | 239.8262 | 4.412861 | Up |
| --- | --- | --- | --- | --- | --- | --- | --- |

**1**

| Unigene35165_All | 3231 | 170 | 1665 | 11.25882 | 119.6863 | 3.41013 | Up |
| --- | --- | --- | --- | --- | --- | --- | --- |

**>Unigene35165_All size 3231 gap 0 0%**

**GCTTTGTTTCTGTGGCTATCTCTTCAATCATTCTGCAATAGATAAACCAATAGTGAGAGAAAGAACCAAAGTCATTTCCTTGCTATTTCCCTCTGTAGATTTTCAACTTTGAAGCATAGAATAAAGTGTGCAAGGAAAAGGCTTAAGCATTGCTTTCTCAAGCTGAGGGAGAAGCTTTTATTATTCCATCATGGTCATGGGCGGCTTCAAGCTCATAAAAATGTGGAATGTGATAGTGGTGCTGTTGCTGCTGGCATCTTCGCTGGTTGGTTCTGCTACAGCCTCTGTGTCCTATGAATCCAAAGCTATCACCATTAATGGCCAAAGAAGGATCCTCATTTCTGGATCCATTCATTACCCCAGAAGCACCCCTGAGATGTGGCCAGATCTTATCCAGAAGGCCAAGGAAGGAGGCTTGGATGTGATTCAGACTTATGTATTCTGGAATGGGCATGAACCTGCACCTGGCGAATATTATTTTGAGGGGAACTATGATCTGGTGAAGTTCATAAAGTTGGTGCAGCAAGCAGGCCTTTATGTTCATCTGCGGATTGGTCCTTATGTCTGTGCTGAGTGGAACTTCGGGGGTTTCCCTGTTTGGCTGAAGTACATTCCAGGTATAAGCTTTAGAACAGACAATGGCCCTTTTAAGTTTCAAATGCGAAAATTTACCGGGAAGATTGTCAACATGATGAAAGCTGAAAGGTTATATGAGTCTCAGGGAGGTCCAATAATTCTATCCCAGATTGAAAATGAATATGGACCTATGGAGTACGAAATTGGCGCTCCTGGTAAATCCTACACTAAGTGGGCAGCAGATATGGCTGTAGGACTTGGTACTGGGGTTCCATGGGTCATGTGCAAGCAAGATGATGCTCCTGATCCTGTTATTAACACTTGCAACGGCTTCTATTGTGATTATTTCTCTCCAAATACAGCTTACAAGCCAAAAATGTGGACAGAAGCTTGGACTGGCTGGTTCACGGAGTTCGGAGGTCCAGTTCCTTATCGGCCTGCTGAAGACTTGGCCTTCTCAGTTGCAAAGTTTATACAGAAAGGGGGATCATTTGTCAATTATTACATGTATCATGGGGGAACAAATTTTGGTCGAACTGCTGGCGGTCCCTTTATTGCTACAAGTTATGATTATGATGCACCTCTTGATGAATATGGACTACTCAGGCAGCCAAAGTGGGGTCATCTGAAGGATTTACACAGAGCAATAAAACTCTGTGAACCTGCTTTAGTATCTGGAGATCCTACTGTAACACGGCTTGGAAACTATCAAGAGGCTCATGTCTTTAAATCAAAGTCTGGAGCTTGTGCTGCATTCCTTGCAAACTATAACCCACGATCTTATGCGACAGTAGCATTTGGAAATTTGCATTACAACTTGCCTCCTTGGTCTATAAGCATTCTTCCTAACTGCAAGCACACTGTTTATAACACTGCAAGGGTTGGTTCCCAGAGTGCACAGATGAAGATGAGTCGTGTTCCTATTCACGGGGGACTCTCTTGGCAAGCATTCAATGAAGAAACAACCTCTACTGATGATAGTTCCTTCACCATGACTGGCTTGTTGGAGCAGTTAAATACAACAAGAGATTTATCTGACTACTTGTGGTACTCCACAGATGTCGTGATTGATCCCAACGAAGGATTTTTGAGGAACGGAAATGGTCCTGTTCTTACAGTATTATCTGCTGGGCATGCTTTGCATGTTTTTGTCAATGGTCAGCTATCAGGAACTGTATATGGGAGCTTAGAATTCCCCAAGCTAACATTTACTGAGAGTGTGAATCTCAGAGCTGGTGTTAACAAAATCTCTCTTCTAAGTGTTGCAGTTGGACTCCCGAATGTTGGTCCACATTTTGAAACATGGAATGCTGGTGTTCTTGGTCCAATTACGTTAAATGGTCTCAATGAGGGGAGAAGGGACTTGTCTTGGCAGAAATGGTCTTACAAGGTTGGTCTTAAAGGTGAAACCTTGAGTCTTCATTCTCTCAGTGGAAGTTCCTCAGTGGAGTGGATTCAAGGGAATTTAATTTCTCGAAGGCAGCCATTGACTTGGTACAAGACTACATTCGATGCCCCGGCTGGAGTTGCACCATTTGCTTTAGACATGGGCAGCATGGGCAAGGGCCAAGTGTGGCTAAATGGACAGAGTCTAGGCCGCTACTGGCCTGCTTATAAAGCATCTGGTACCTGTGATTATTGTAACTATGCTGGAACTTATAATGAGAATAAATGCAGAAGTAACTGTGGCGAGGCTTCTCAAAGATGGTATCATGTTCCTCATTCATGGCTGAAGCCAACTGGAAATTTATTGGTTGTGTTTGAAGAATTGGGTGGAGATCCCAATGGGATCTTTTTGGTTAGAAGGGATATAGATAGTGTGTGTGCTGATATTTATGAGTGGCAGCCAAATGTTAGAAGTTATCAGATGCAAGCTTCTGGCAAAGTTAGCAAACCTGTGAGACCAAAAGCACATTTATCATGTGGCCCTGGACAGAAAATCTCATCAATCAAATTTGCTAGCTTTGGCACTCCAGTAGGGTCTTGTGGAAACTTCCATGAAGGAAGCTGCCA****TGCTCACAAGTCATAT****GATGCCTTTCAAAGGAATTGTGTTGGACAGAACTGGTGCACAGTAACAGTGTCACCTGAAAATTTTGGAGGAGATCCATGTCCAAATGTCATGAAGAAACTCTCAGTGGAAGCCATTTGCACCTGATCATTTCAACCTCAGAAAATAAATGGAATAAGATTGATTTTGTATGCAATTCAATACAGGGTTTTTGATCAACCAAGTCCTCACTTCTAGATCACCACAAACATTGATTTGGCTTTACCAAGGTGAAGTTGCAGATAACACAACACACCAGCTGGTTGAAAGCCTGTAGAAATTGCTGAGATGATTGTGGATATGTGCATATATATTGGTATTCTGCTTGTTATACAGAAGTGAAAGAAAGGTGAAGATTTGATTGATGCACAATGAGACGATACTGATTGAGTCCATGTGCAAAATGTTTTTGTTCCAGTGTGGAAGCCTCTAGCTTCCTGTAATATTATTTTCCCTTGTAAGCTGCATTCATAGCCACTTCCTTGCTTTCTTCTTGTAAGAAACTTCCCGAGATGTTAAGATCAGGAACATTAAATTCATAGCTGTTTTGTTTCTGTTTCATGCTTTATTTTCCACATGATCCTCCTGTTGAGTTCCTAGTTTGGGCT**

>**[AT3G13750](http://www.arabidopsis.org/servlets/TairObject?type=locus&name=AT3G13750).1** | Symbols: BGAL1 | beta galactosidase 1 | chr3:4511192-4515756

FORWARD LENGTH=847

Length = 847

Plus Strand HSPs:

Score = 3757 (1327.6 bits), Expect = 0., P = 0.

Identities = 654/811 (80%), Positives = 736/811 (90%), Frame = +2

Query: 293 YESKAITINGQRRILISGSIHYPRSTPEMWPDLIQKAKEGGLDVIQTYVFWNGHEPAPGE 472

Y+S+AITING+RRILISGSIHYPRSTPEMWPDLI+KAKEGGLDVIQTYVFWNGHEP+PG+

Sbjct: 36 YDSRAITINGKRRILISGSIHYPRSTPEMWPDLIRKAKEGGLDVIQTYVFWNGHEPSPGK 95

Query: 473 YYFEGNYDLVKFIKLVQQAGLYVHLRIGPYVCAEWNFGGFPVWLKYIPGISFRTDNGPFK 652

YYFEGNYDLVKF+KLVQQ+GLY+HLRIGPYVCAEWNFGGFPVWLKYIPGISFRTDNGPFK

Sbjct: 96 YYFEGNYDLVKFVKLVQQSGLYLHLRIGPYVCAEWNFGGFPVWLKYIPGISFRTDNGPFK 155

Query: 653 FQMRKFTGKIVNMMKAERLYESQGGPIILSQIENEYGPMEYEIGAPGKSYTKWAADMAVG 832

QM++FT KIVNMMKAERL+ESQGGPIILSQIENEYGPMEYE+GAPG+SYT WAA MAVG

Sbjct: 156 AQMQRFTTKIVNMMKAERLFESQGGPIILSQIENEYGPMEYELGAPGRSYTNWAAKMAVG 215

Query: 833 LGTGVPWVMCKQDDAPDPVINTCNGFYCDYFSPNTAYKPKMWTEAWTGWFTEFGGPVPYR 1012

LGTGVPWVMCKQDDAPDP+IN CNGFYCDYFSPN AYKPKMWTEAWTGWFT+FGGPVPYR

Sbjct: 216 LGTGVPWVMCKQDDAPDPIINACNGFYCDYFSPNKAYKPKMWTEAWTGWFTKFGGPVPYR 275

Query: 1013 PAEDLAFSVAKFIQKGGSFVNYYMYHGGTNFGRTAGGPFIATSYDYDAPLDEYGLLRQPK 1192

PAED+AFSVA+FIQKGGSF+NYYMYHGGTNFGRTAGGPFIATSYDYDAPLDEYGL RQPK

Sbjct: 276 PAEDMAFSVARFIQKGGSFINYYMYHGGTNFGRTAGGPFIATSYDYDAPLDEYGLERQPK 335

Query: 1193 WGHLKDLHRAIKLCEPALVSGDPTVTRLGNYQEAHVFKSKSGACAAFLANYNPRSYATVA 1372

WGHLKDLHRAIKLCEPALVSG+PT LGNYQEAHV+KSKSGAC+AFLANYNP+SYA V+

Sbjct: 336 WGHLKDLHRAIKLCEPALVSGEPTRMPLGNYQEAHVYKSKSGACSAFLANYNPKSYAKVS 395

Query: 1373 FGNLHYNLPPWSISILPNCKHTVYNTARVGSQSAQMKMSRVPIHGGLSWQAFNXXXXXXX 1552

FGN HYNLPPWSISILP+CK+TVYNTARVG+Q+++MKM RVP+HGGLSWQA+N

Sbjct: 396 FGNNHYNLPPWSISILPDCKNTVYNTARVGAQTSRMKMVRVPVHGGLSWQAYNEDPSTYI 455

Query: 1553 XXXFTMTGLLEQLNTTRDLSDYLWYSTDVVIDPNEGFLRNGNGPVLTVLSAGHALHVFVN 1732

FTM GL+EQ+NTTRD SDYLWY TDV +D NEGFLRNG+ P LTVLSAGHA+HVF+N

Sbjct: 456 DESFTMVGLVEQINTTRDTSDYLWYMTDVKVDANEGFLRNGDLPTLTVLSAGHAMHVFIN 515

Query: 1733 GQLSGTVYGSLEFPKLTFTESVNLRAGVNKISLLSVAVGLPNVGPHFETWNAGVLGPITL 1912

GQLSG+ YGSL+ PKLTF + VNLRAG NKI++LS+AVGLPNVGPHFETWNAGVLGP++L

Sbjct: 516 GQLSGSAYGSLDSPKLTFRKGVNLRAGFNKIAILSIAVGLPNVGPHFETWNAGVLGPVSL 575

Query: 1913 NGLNEGRRDLSWQKWSYKVGLKGETXXXXXXXXXXXVEWIQGNLISRRQPLTWYKTTFDA 2092

NGLN GRRDLSWQKW+YKVGLKGE+ VEW +G ++++QPLTWYKTTF A

Sbjct: 576 NGLNGGRRDLSWQKWTYKVGLKGESLSLHSLSGSSSVEWAEGAFVAQKQPLTWYKTTFSA 635

Query: 2093 PAGVAPFALDMGSMGKGQVWLNGQSLGRYWPAYKASGTCDYCNYAGTYNENKCRSNCGEA 2272

PAG +P A+DMGSMGKGQ+W+NGQSLGR+WPAYKA G+C C+Y GT+ E+KC NCGEA

Sbjct: 636 PAGDSPLAVDMGSMGKGQIWINGQSLGRHWPAYKAVGSCSECSYTGTFREDKCLRNCGEA 695

Query: 2273 SQRWYHVPHSWLKPTGNLLVVFEELGGDPNGIFLVRRDIDSVCADIYEWQPNVRSYQMQA 2452

SQRWYHVP SWLKP+GNLLVVFEE GGDPNGI LVRR++DSVCADIYEWQ + +YQ+ A

Sbjct: 696 SQRWYHVPRSWLKPSGNLLVVFEEWGGDPNGITLVRREVDSVCADIYEWQSTLVNYQLHA 755

Query: 2453 SGKVSKPVRPKAHLSCGPGQKISSIKFASFGTPVGSCGNFHEGSCHAHKSYDAFQRNCVG 2632

SGKV+KP+ PKAHL CGPGQKI+++KFASFGTP G+CG++ +GSCHAH SYDAF + CVG

Sbjct: 756 SGKVNKPLHPKAHLQCGPGQKITTVKFASFGTPEGTCGSYRQGSCHAHHSYDAFNKLCVG 815

Query: 2633 QNWCTVTVSPENFGGDPCPNVMKKLSVEAIC 2725

QNWC+VTV+PE FGGDPCPNVMKKL+VEA+C

Sbjct: 816 QNWCSVTVAPEMFGGDPCPNVMKKLAVEAVC 846

OLIGO [start](http://bioinfo.ut.ee/primer3-0.4.0/primer3_www_results_help.html#PRIMER_START)  [len](http://bioinfo.ut.ee/primer3-0.4.0/primer3_www_results_help.html#PRIMER_LEN)  [tm](http://bioinfo.ut.ee/primer3-0.4.0/primer3_www_results_help.html#PRIMER_TM)  [gc%](http://bioinfo.ut.ee/primer3-0.4.0/primer3_www_results_help.html#PRIMER_GC)  [any](http://bioinfo.ut.ee/primer3-0.4.0/primer3_www_results_help.html#PRIMER_ANY)  [3'](http://bioinfo.ut.ee/primer3-0.4.0/primer3_www_results_help.html#PRIMER_REPEAT) [seq](http://bioinfo.ut.ee/primer3-0.4.0/primer3_www_results_help.html#PRIMER_OLIGO_SEQ)

LEFT PRIMER 34 21 59.63 47.62 5.00 1.00 TTGTGTTGGACAGAACTGGTG

RIGHT PRIMER 157 22 59.59 45.45 6.00 2.00 CTGAGGTTGAAATGATCAGGTG

SEQUENCE SIZE: 269

INCLUDED REGION SIZE: 269

PRODUCT SIZE: 124, PAIR ANY COMPL: 3.00, PAIR 3' COMPL: 1.00

1 TGCTCACAAGTCATATGATGCCTTTCAAAGGAATTGTGTTGGACAGAACTGGTGCACAGT

>>>>>>>>>>>>>>>>>>>>>

61 AACAGTGTCACCTGAAAATTTTGGAGGAGATCCATGTCCAAATGTCATGAAGAAACTCTC

121 AGTGGAAGCCATTTGCACCTGATCATTTCAACCTCAGAAAATAAATGGAATAAGATTGAT

<<<<<<<<<<<<<<<<<<<<<<

181 TTTGTATGCAATTCAATACAGGGTTTTTGATCAACCAAGTCCTCACTTCTAGATCACCAC

241 AAACATTGATTTGGCTTTACCAAGGTGAA

KEYS (in order of precedence):

>>>>>> left primer

<<<<<< right primer

ADDITIONAL OLIGOS

[start](http://bioinfo.ut.ee/primer3-0.4.0/primer3_www_results_help.html#PRIMER_START)  [len](http://bioinfo.ut.ee/primer3-0.4.0/primer3_www_results_help.html#PRIMER_LEN)  [tm](http://bioinfo.ut.ee/primer3-0.4.0/primer3_www_results_help.html#PRIMER_TM)  [gc%](http://bioinfo.ut.ee/primer3-0.4.0/primer3_www_results_help.html#PRIMER_GC)  [any](http://bioinfo.ut.ee/primer3-0.4.0/primer3_www_results_help.html#PRIMER_ANY)  [3'](http://bioinfo.ut.ee/primer3-0.4.0/primer3_www_results_help.html#PRIMER_REPEAT) [seq](http://bioinfo.ut.ee/primer3-0.4.0/primer3_www_results_help.html#PRIMER_OLIGO_SEQ)

1 LEFT PRIMER 33 22 59.93 45.45 5.00 1.00 ATTGTGTTGGACAGAACTGGTG

RIGHT PRIMER 157 22 59.59 45.45 6.00 2.00 CTGAGGTTGAAATGATCAGGTG

PRODUCT SIZE: 125, PAIR ANY COMPL: 3.00, PAIR 3' COMPL: 1.00

**6 EXO**

| CL5360.Contig1_All | 435 | 237 | 1646 | 116.5843 | 815.5092 | 2.806327 | Up |
| --- | --- | --- | --- | --- | --- | --- | --- |

24

| CL5360.Contig1_All | 435 | 237 | 427 | 116.5843 | 228.2796 | 0.969428 | Up |
| --- | --- | --- | --- | --- | --- | --- | --- |

1

| CL5360.Contig1_All | 435 | 237 | 1150 | 116.5843 | 614.0107 | 2.39689 | Up |
| --- | --- | --- | --- | --- | --- | --- | --- |

>CL5360.Contig1_All size 435 gap 0 0%

GGAAAGAGGAGAAGGATTGTTTTTCTTAGAGGTGAGACGGTAGTATTTTTCGGTGGTTTTCCACCACGTGGCAACAGAGGGTTGGTTGGTTTGTGCCGCTGGGCGGGATGAGAGGGAGGTGATGAAATCGGTGATGATGGCCTTTTGGGATGGTTTGAAGTTACCATACCAGATGAGGTTTACAGAGATTTTGCCATATAGAAGAGGACCGTTATGGTAATGAAGCAGCTGAGACTGGTCCTGAACCAGCTCATTCAGACTCCTAGCAGCGAAGGAGATCTGAAATACGGATATGAGAAACAAGATTTTGAAAAAGTATTGGGAAGAAACAAAAGTAGTAGTCATTTTGTTTTGTTAATAGAGCAGAGCCAAGCAGAAAAACGCTGACGGCTTATTTTTTGAAATGGATCAGACAGCAGAGATTGCTATTGTAAT

ATTACAATAGCAATCTCTGCTGTCTGATCCATTTCAAAAAATAAGCCGTCAGCGTTTTTCTGCTTGGCTCTGCTCTATTAACAAAACAAAATGACTACTACTTTTGTTTCTTCCCAATACTTTTTCAAAATCTTGTTTCTCATATCCGTATTTCAGATCTCCTTCGCTGCTAGGAGTCTGAATGAGCTGGTTCAGGACCAGTCTCAGCTGCTTCATTACCATAACGGTCCTCTTCTATATGGCAAAATCTCTGTAAACCTCATCTGGTATGGTAACTTCAAACCATCCCAAAAGGCCATCATCACCGATTTCATCACCTCCCTCTCATCCCGCCCAGCGGCACAAACCAACCAACCCTCTGTTGCCACGTGGTGGAAAACCACCGAAAAATACTACCGTCTCACCTCTAAGAAAAACAATCCTTCTCCTCTTTCC

>**[AT4G08950](http://www.arabidopsis.org/servlets/TairObject?type=locus&name=AT4G08950).1** | Symbols: EXO | Phosphate-responsive 1 family protein |

chr4:5740378-5741322 FORWARD LENGTH=314

Length = 314

Plus Strand HSPs:

Score = 332 (121.9 bits), Expect = 2.5e-30, P = 2.5e-30

Identities = 66/106 (62%), Positives = 81/106 (76%), Frame = +1

Query: 124 FKILFLISVFQISFAARSLNELVQDQSQLLHYHNGPLLYGKISVNLIWYGNFKPSQKAII 303

FK+ +S+ QIS +AR+L +Q QLL YH G LL GKISVNLIWYG FKPSQ+AII

Sbjct: 6 FKLFLFLSLLQISVSARNLASQEPNQFQLLKYHKGALLSGKISVNLIWYGKFKPSQRAII 65

Query: 304 TDFITSLS-SRPAAQT-NQPSVATWWKTTEKYYRLTSKKNNPSPLS 435

+DFITSL+ + P ++T +QPSVATWWKTTEKYY+L + N SPLS

Sbjct: 66 SDFITSLTHTSPTSKTLHQPSVATWWKTTEKYYKLATPSKNSSPLS 111

OLIGO [start](http://bioinfo.ut.ee/primer3-0.4.0/primer3_www_results_help.html#PRIMER_START)  [len](http://bioinfo.ut.ee/primer3-0.4.0/primer3_www_results_help.html#PRIMER_LEN)  [tm](http://bioinfo.ut.ee/primer3-0.4.0/primer3_www_results_help.html#PRIMER_TM)  [gc%](http://bioinfo.ut.ee/primer3-0.4.0/primer3_www_results_help.html#PRIMER_GC)  [any](http://bioinfo.ut.ee/primer3-0.4.0/primer3_www_results_help.html#PRIMER_ANY)  [3'](http://bioinfo.ut.ee/primer3-0.4.0/primer3_www_results_help.html#PRIMER_REPEAT) [seq](http://bioinfo.ut.ee/primer3-0.4.0/primer3_www_results_help.html#PRIMER_OLIGO_SEQ)

LEFT PRIMER 4 21 59.62 52.38 6.00 2.00 GACCAGTCTCAGCTGCTTCAT

RIGHT PRIMER 169 20 59.86 50.00 2.00 0.00 CAGAGGGTTGGTTGGTTTGT

SEQUENCE SIZE: 243

INCLUDED REGION SIZE: 243

PRODUCT SIZE: 166, PAIR ANY COMPL: 4.00, PAIR 3' COMPL: 0.00

1 CAGGACCAGTCTCAGCTGCTTCATTACCATAACGGTCCTCTTCTATATGGCAAAATCTCT

>>>>>>>>>>>>>>>>>>>>>

61 GTAAACCTCATCTGGTATGGTAACTTCAAACCATCCCAAAAGGCCATCATCACCGATTTC

121 ATCACCTCCCTCTCATCCCGCCCAGCGGCACAAACCAACCAACCCTCTGTTGCCACGTGG

<<<<<<<<<<<<<<<<<<<<

181 TGGAAAACCACCGAAAAATACTACCGTCTCACCTCTAAGAAAAACAATCCTTCTCCTCTT

241 TCC

KEYS (in order of precedence):

>>>>>> left primer

<<<<<< right primer

ADDITIONAL OLIGOS

[start](http://bioinfo.ut.ee/primer3-0.4.0/primer3_www_results_help.html#PRIMER_START)  [len](http://bioinfo.ut.ee/primer3-0.4.0/primer3_www_results_help.html#PRIMER_LEN)  [tm](http://bioinfo.ut.ee/primer3-0.4.0/primer3_www_results_help.html#PRIMER_TM)  [gc%](http://bioinfo.ut.ee/primer3-0.4.0/primer3_www_results_help.html#PRIMER_GC)  [any](http://bioinfo.ut.ee/primer3-0.4.0/primer3_www_results_help.html#PRIMER_ANY)  [3'](http://bioinfo.ut.ee/primer3-0.4.0/primer3_www_results_help.html#PRIMER_REPEAT) [seq](http://bioinfo.ut.ee/primer3-0.4.0/primer3_www_results_help.html#PRIMER_OLIGO_SEQ)

1 LEFT PRIMER 4 21 59.62 52.38 6.00 2.00 GACCAGTCTCAGCTGCTTCAT

RIGHT PRIMER 168 20 59.86 50.00 1.00 0.00 AGAGGGTTGGTTGGTTTGTG

PRODUCT SIZE: 165, PAIR ANY COMPL: 4.00, PAIR 3' COMPL: 1.00

2 LEFT PRIMER 4 21 59.62 52.38 6.00 2.00 GACCAGTCTCAGCTGCTTCAT

RIGHT PRIMER 170 20 59.86 50.00 2.00 0.00 ACAGAGGGTTGGTTGGTTTG

PRODUCT SIZE: 167, PAIR ANY COMPL: 4.00, PAIR 3' COMPL: 1.00

3 LEFT PRIMER 4 21 59.62 52.38 6.00 2.00 GACCAGTCTCAGCTGCTTCAT

RIGHT PRIMER 172 20 59.86 50.00 4.00 0.00 CAACAGAGGGTTGGTTGGTT

PRODUCT SIZE: 169, PAIR ANY COMPL: 4.00, PAIR 3' COMPL: 0.00

4 LEFT PRIMER 13 22 59.80 45.45 6.00 3.00 CAGCTGCTTCATTACCATAACG

RIGHT PRIMER 138 20 60.01 55.00 2.00 2.00 GGATGAGAGGGAGGTGATGA

PRODUCT SIZE: 126, PAIR ANY COMPL: 6.00, PAIR 3' COMPL: 3.00

**7. EXO**

72h

| Unigene980_All | 844 | 217 | 685 | 55.01718 | 188.6769 | 1.777964 | Up |
| --- | --- | --- | --- | --- | --- | --- | --- |

24h

| Unigene980_All | 844 | 217 | 362 | 55.01718 | 99.7458 | 0.858374 | Up |
| --- | --- | --- | --- | --- | --- | --- | --- |

1h

| Unigene980_All | 844 | 217 | 927 | 55.01718 | 255.0966 | 2.213089 | Up |
| --- | --- | --- | --- | --- | --- | --- | --- |

>Unigene980_All size 844 gap 0 0%

TTTGGTCCAAAAGTAATATACTTATATTTAAAAGCAAACCAAAGTAAATTTGCTTTGATATCACTAATCCGCATAAGCAAAAAGAAATAAACAAATCAATGAATTAACGCTACATAACGCTTATTTGTACAACTTACCGTTCCCCCATTTCTAATACAGCAGTACTAGTGGAATTAAGCATCAGTATGTTAACCCTCACACAAGCGTTGAGCATGATGATGTAGAAGGATCATACAAAGCAGGAAGCAGAAACTTCCTCCCATTACCACCATTAGCATTGTAGCTAGCACCAGTAGTAGGGTCCACCAACAAGTTACCAGCATAACCCGGGTAAGCGCCTTTTTCATAAACCCCCAGGACAAGCTGATGCAGCTTCGAGTGGTGCCTCGGGTGAACCCTGGAAGTAACCATTTCCAAATGGGTTGGTGGCAGTTCCAGCCAATAGGGTAGCTAGGTTGATCACCATTCCATCAACACCCACATCGTTGTTAGGTGCAACCAAAGGTAGGCTTTGAGGCCCATAAATTGGTTGGTGGAATGGCCACGCGCATTGACCAGGGCATTGAGTTTCAGAATTACCAGCCCAAATGTAAGCGAACTTGTAGTTCTTTCCCTTCACCACAGAAGAAGAAGAAGAAGAAGACCCGTGAGTACCGCAACGGCTTCATGCAGAAAGCTTCGACCGCAACGTCAGCGGATAGTGAGAACTAACGTTTGATTGGCGTTTCTCTGGTCACCTTTTGGAAGCCAATTGAAGGATATGCTTGTTGGTTAGTGATTTTCCGAGTGTGTAGTTCTGGTAAATCTGGTTACCCAATGAAAGGGAAAGAGGAGAAGGATTGTT

AACAATCCTTCTCCTCTTTCCCTTTCATTGGGTAACCAGATTTACCAGAACTACACACTCGGAAAATCACTAACCAACAAGCATATCCTTCAATTGGCTTCCAAAAGGTGACCAGAGAAACGCCAATCAAACGTTAGTTCTCACTATCCGCTGACGTTGCGGTCGAAGCTTTCTGCATGAAGCCGTTGCGGTACTCACGGGTCTTCTTCTTCTTCTTCTTCTGTGGTGAAGGGAAAGAACTACAAGTTCGCTTACATTTGGGCTGGTAATTCTGAAACTCAATGCCCTGGTCAATGCGCGTGGCCATTCCACCAACCAATTTATGGGCCTCAAAGCCTACCTTTGGTTGCACCTAACAACGATGTGGGTGTTGATGGAATGGTGATCAACCTAGCTACCCTATTGGCTGGAACTGCCACCAACCCATTTGGAAATGGTTACTTCCAGGGTTCACCCGAGGCACCACTCGAAGCTGCATCAGCTTGTCCTGGGGGTTTATGAAAAA**GGCGCTTACCCGGGTTATGCTGGTAACTTGTTGGT****GGACCCTACTACTGGTGCTAGCTACAATGCTAATGGTGGTAATGGGAGGAAGTTTCTGCTTCCTGCTTTGTATGATCCTTCTACATCATCATGCTCAACGCTTGTGTGAGGGTTAACATACTGATGCTTAATTCCACTAGTACTGCTGTATTAGAAATGGGGGAACGGTAAGTTGTACAAATAAGCGTTATGTAGCGTTAATTCATTGATTTGTTTATTT**CTTTTTGCTTATGCGGATTAGTGATATCAAAGCAAATTTACTTTGGTTTGCTTTTAAATATAAGTATATTACTTTTGGACCAAA

>[**AT4G08950**](http://www.arabidopsis.org/servlets/TairObject?type=locus&name=AT4G08950)**.1** | Symbols: EXO | Phosphate-responsive 1 family protein |

chr4:5740378-5741322 FORWARD LENGTH=314

Length = 314

Plus Strand HSPs:

Score = 511 (184.9 bits), Expect = 1.8e-71, Sum P(2) = 1.8e-71

Identities = 108/173 (62%), Positives = 122/173 (70%), Frame = +1

Query: 4 NPSPLSLSLGNQIY-QNYTLGKSLTNKHILQLASKR*PEKRQSNVSSHYPLTLRSKLSA* 180

N SPLSL+LG QI ++ +LGKSLT+K I LASK + V + +T+

Sbjct: 106 NSSPLSLTLGKQIIDESCSLGKSLTDKKIQTLASKGDQRNAINVVLTSADVTVTG--FGM 163

Query: 181 SRCGTHGXXXXXXXVVKGKNYKFAYIWAGNSETQCPGQCAWPFHQPIYGPQSLPLVAPNN 360

SRCGTHG +G KFAYIW GNSETQCPGQCAWPFH P+YGPQS PLVAPNN

Sbjct: 164 SRCGTHGHARGLGK--RGS--KFAYIWVGNSETQCPGQCAWPFHAPVYGPQSPPLVAPNN 219

Query: 361 DVGVDGMVINLATLLAGTATNPFGNGYFQGSPEAPLEAASACPGGL*KRRLPG 519

DVG+DGMVINLA+LLAGTATNPFGNGY+QG APLEAASACPG K PG

Sbjct: 220 DVGLDGMVINLASLLAGTATNPFGNGYYQGPQNAPLEAASACPGVYGKGAYPG 272

Score = 228 (85.3 bits), Expect = 1.8e-71, Sum P(2) = 1.8e-71

Identities = 42/52 (80%), Positives = 47/52 (90%), Frame = +2

Query: 491 GVYEKGAYPGYAGNLLVDPTTGASYNANGGNGRKFLLPALYDPSTSSCSTLV 646

GVY KGAYPGYAG+LLVD TTG S+NA G NGRKFLLPALYDP+TS+CST+V

Sbjct: 263 GVYGKGAYPGYAGDLLVDTTTGGSFNAYGANGRKFLLPALYDPTTSACSTMV 314

OLIGO [start](http://bioinfo.ut.ee/primer3-0.4.0/primer3_www_results_help.html#PRIMER_START)  [len](http://bioinfo.ut.ee/primer3-0.4.0/primer3_www_results_help.html#PRIMER_LEN)  [tm](http://bioinfo.ut.ee/primer3-0.4.0/primer3_www_results_help.html#PRIMER_TM)  [gc%](http://bioinfo.ut.ee/primer3-0.4.0/primer3_www_results_help.html#PRIMER_GC)  [any](http://bioinfo.ut.ee/primer3-0.4.0/primer3_www_results_help.html#PRIMER_ANY)  [3'](http://bioinfo.ut.ee/primer3-0.4.0/primer3_www_results_help.html#PRIMER_REPEAT) [seq](http://bioinfo.ut.ee/primer3-0.4.0/primer3_www_results_help.html#PRIMER_OLIGO_SEQ)

LEFT PRIMER 56 20 60.11 50.00 3.00 2.00 CTGAAACTCAATGCCCTGGT

RIGHT PRIMER 203 20 60.66 55.00 5.00 2.00 GTGGCAGTTCCAGCCAATAG

SEQUENCE SIZE: 273

INCLUDED REGION SIZE: 273

PRODUCT SIZE: 148, PAIR ANY COMPL: 6.00, PAIR 3' COMPL: 2.00

1 TCTTCTGTGGTGAAGGGAAAGAACTACAAGTTCGCTTACATTTGGGCTGGTAATTCTGAA

>>>>>

61 ACTCAATGCCCTGGTCAATGCGCGTGGCCATTCCACCAACCAATTTATGGGCCTCAAAGC

>>>>>>>>>>>>>>>

121 CTACCTTTGGTTGCACCTAACAACGATGTGGGTGTTGATGGAATGGTGATCAACCTAGCT

181 ACCCTATTGGCTGGAACTGCCACCAACCCATTTGGAAATGGTTACTTCCAGGGTTCACCC

<<<<<<<<<<<<<<<<<<<<

241 GAGGCACCACTCGAAGCTGCATCAGCTTGTCCT

KEYS (in order of precedence):

>>>>>> left primer

<<<<<< right primer

ADDITIONAL OLIGOS

[start](http://bioinfo.ut.ee/primer3-0.4.0/primer3_www_results_help.html#PRIMER_START)  [len](http://bioinfo.ut.ee/primer3-0.4.0/primer3_www_results_help.html#PRIMER_LEN)  [tm](http://bioinfo.ut.ee/primer3-0.4.0/primer3_www_results_help.html#PRIMER_TM)  [gc%](http://bioinfo.ut.ee/primer3-0.4.0/primer3_www_results_help.html#PRIMER_GC)  [any](http://bioinfo.ut.ee/primer3-0.4.0/primer3_www_results_help.html#PRIMER_ANY)  [3'](http://bioinfo.ut.ee/primer3-0.4.0/primer3_www_results_help.html#PRIMER_REPEAT) [seq](http://bioinfo.ut.ee/primer3-0.4.0/primer3_www_results_help.html#PRIMER_OLIGO_SEQ)

1 LEFT PRIMER 56 20 60.11 50.00 3.00 2.00 CTGAAACTCAATGCCCTGGT

RIGHT PRIMER 220 20 60.98 45.00 8.00 0.00 CATTTCCAAATGGGTTGGTG

PRODUCT SIZE: 165, PAIR ANY COMPL: 4.00, PAIR 3' COMPL: 0.00

2 LEFT PRIMER 59 20 60.50 45.00 5.00 3.00 AAACTCAATGCCCTGGTCAA

RIGHT PRIMER 203 20 60.66 55.00 5.00 2.00 GTGGCAGTTCCAGCCAATAG

PRODUCT SIZE: 145, PAIR ANY COMPL: 4.00, PAIR 3' COMPL: 1.00

3 LEFT PRIMER 56 20 60.11 50.00 3.00 2.00 CTGAAACTCAATGCCCTGGT

RIGHT PRIMER 179 21 59.95 47.62 6.00 1.00 GCTAGGTTGATCACCATTCCA

PRODUCT SIZE: 124, PAIR ANY COMPL: 4.00, PAIR 3' COMPL: 3.00

4 LEFT PRIMER 101 20 59.55 45.00 5.00 2.00 CAATTTATGGGCCTCAAAGC

RIGHT PRIMER 220 20 60.98 45.00 8.00 0.00 CATTTCCAAATGGGTTGGTG

PRODUCT SIZE: 120, PAIR ANY COMPL: 4.00, PAIR 3' COMPL: 2.00

**8 E3 ubiquitin-protein ligase RMA1H1-like isoform 1，**RMA1 encodes a novel 28 kDa protein with a RING finger motif and a C-terminal membrane-anchoring domain that is involved in the secretory pathway. Has E3 ubiquitin ligase activity.

72

| Unigene2216_All | 1277 | 270 | 1061 | 45.24327 | 179.0659 | 1.984715 | Up |
| --- | --- | --- | --- | --- | --- | --- | --- |

24

| Unigene2216_All | 1277 | 270 | 1528 | 45.24327 | 278.2665 | 2.620692 | Up |
| --- | --- | --- | --- | --- | --- | --- | --- |

1

| Unigene2216_All | 1277 | 270 | 509 | 45.24327 | 92.57511 | 1.032921 | Up |
| --- | --- | --- | --- | --- | --- | --- | --- |

>Unigene2216_All size 1277 gap 0 0%

TTGGTTCAAAAGTAAATACATTCTTACTTATTTACTTTATAAACAAGCTCACTTTATAGGATTTCTCTTATCTCTCCTCCTTTTGTAGTAAAACGAGTGAGATATATAACTTGTGAAAGGCTAAAACTAAAACACCACGTTAATAACTTATACAAACATCTATTGTTGCTCAAAGCGAGACTGGTAATTGTACATATCTCAGCATTAATTTGCAACTTGTCTAGCATATTATACATCAGGCAGAATCTGCGAACAAACTAATAAATTGCATTCTATACAAGGATGTATTAGACAAGATGAAATTGAAATAGCATTCAGAATAGGAGAAGACAAAAAACTACGCAGCAAAGGAGGAAAAAACAGATTCTACTAAGTGATTCATCAGCTTGCATTAAATGCCTTCTGATCCTTGGGTTACTGTTCCCTGAATGATGATACGAATTAGGATATGTATATATGTTTGTCACCTGGTTACCAAAGACCGTAGCAAGTATGATACCACCAAAGATTCCAAATGAGGTATCTTGTAAACCACTTGTGCTAAGCATTGGTGAAGTGTAACTGCTTGGAATGGAGTCAAATTGTTGAGGATGATAAGGAGGACGGGGATGATAACTTTGATAAGTTGGATGGGAAACAGTTGTAGTATTATAAGATCTGGGAGGAAGGGGTCTTCGCGGCATGACAATCCCTACTTGATGGGCCTTTCCTTCAGAAGTTCTTGTGGTTTGGCTGCGGCAATATAGTGGAACTAAGGAGGATTCAGAAACTTCTGATTTGCATACTGGACATTGTGGCTTCTGCTGCTCTTCGTTTTCTTGTAAGAGTTACTTTCGGAAATTAAGCCATTTGTAAATGCAGGGCCAGCAGTAGAGATGACCACAAAGAGTGACCACTGGATCTTGCACACTCCAGGCAGATGTTGCAGTCAAAGCCACCAGAGGCATTTCTATCAGAATCTGCAATAGCATCACTGGGAGATTTCCACTTTTCTAAAGATGGTTTATCTTCAAAGGAATTTATTTGGGGCACAGCCTCCTCAAAGTACTGGTCTAAGGCCATTGTTTCTTTTGTTTTTACAATATGCTCTTTTGTCAGATGCCTGAACCTGAAACGCATGTAACAATCCTGAGTGTCTGATATCAGATTAGTTCATCCTTCATAAACGAAGTTTTTGTGAAGATGAAGGGCTTAAAAATCAGTCTGCAAATTCATAACGATGAAGTACTCCTCAAAACTGATTCAGTTTTGATAAAAAAAAAAAAAGATGCCAATGT

ACATTGGCATCTTTTTTTTTTTTTATCAAAACTGAATCAGTTTTGAGGAGTACTTCATCGTTATGAATTTGCAGACTGATTTTTAAGCCCTTCATCTTCACAAAAACTTCGTTTATGAAGGATGAACTAATCTGATATCAGACACTCAGGATTGTTACATGCGTTTCAGGTTCAGGCATCTGACAAAAGAGCATATTGTAAAAACAAAAGAAACAATGGCCTTAGACCAGTACTTTGAGGAGGCTGTGCCCCAAATAAATTCCTTTGAAGATAAACCATCTTTAGAAAAGTGGAAATCTCCCAGTGATGCTATTGCAGATTCTGATAGAAATGCCTCTGGTGGCTTTGACTGCAACATCTGCCTGGAGTGTGCAAGATCCAGTGGTCACTCTTTGTGGTCATCTCTACTGCTGGCCCTGCATTTACAAATGGCTTAATTTCCGAAAGTAACTCTTACAAGAAAACGAAGAGCAGCAGAAGCCACAATGTCCAGTATGCAAATCAGAAGTTTCTGAATCCTCCTTAGTTCCACTATATTGCCGCAGCCAAACCACAAGAACTTCTGAAGGAAAGGCCCATCAAGTAGGGATTGTCATGCCGCGAAGACCCCTTCCTCCCAGATCTTATAATACTACAACTGTTTCCCATCCAACTTATCAAAGTTATCATCCCCGTCCTCCTTATCATCCTCAACAATTTGACTCCATTCCAAGCAGTTACACTTCACCAATGCTTAGCACAAGTGGTTTACAAGATACCTCATTTGGAATCTTTGGTGGTATCATACTTGCTACGGTCTTTGGTAACCAGGTGACAAACATATATACATATCCTAATTCGTATCATCATTCAGGGAACAGTAACCCAAGGATCAGAAGGCATTTAATGCAAGCTGATGAATCACTTAGTAGAATCTGTTTTTTCCTCCTTTGCTGCGTAGTTTTTTGTCTTCTCCTATTCTGAATGCTATTTCAATTTCATCTTGTCTAATACATCCTTGTATAGAATGCAATTTATTAGTTTGTTCGCAGATTCTGCCTGATGTATAATATGCTAGACAAGTTGCAAATTAATGCTGAGATATGTACAATTACCAGTCTCGCTTTGAGCAACAATAGATGTTTGTATAAGTTATTAACGTGGTGTTTTAGTTTTAGCCTTTCACAAGTTATATATCTCACTCGTTTTACTACAAAAGGAGGAGAGATAAGAGAAATCCTATAAAGTGAGCTTGTTTATAAAGTAAATAAGTAAGAATGTATTTACTTTTGAACCAA

>**[AT4G03510](http://www.arabidopsis.org/servlets/TairObject?type=locus&name=AT4G03510).1** | Symbols: RMA1, ATRMA1 | RING membrane-anchor 1 |

chr4:1557905-1558654 REVERSE LENGTH=249

Length = 249

Plus Strand HSPs:

Score = 350 (128.3 bits), Expect = 2.4e-38, Sum P(2) = 2.4e-38

Identities = 80/189 (42%), Positives = 113/189 (59%), Frame = +1

Query: 367 SVQDPVVTLCGHLYCWPCIYKWLNFRK*LLQENEEQQKPQCPVCKSEVSESSLVPLYCRS 546

SVQ+PVVTLCGHL+CWPCI+KWL+ + +E Q+ QCPVCKS+VS S+LVPLY R

Sbjct: 54 SVQEPVVTLCGHLFCWPCIHKWLDVQS-FSTSDEYQRHRQCPVCKSKVSHSTLVPLYGRG 112

Query: 547 QTTRTSEGKAHQVGIVMPRRPLPPRSYNTTTVSHPTYQSYHPR--PPYH---PQQ-FDSI 708

+ T EGK +P+RP+ P Y + P Y S R H PQ+ + +

Sbjct: 113 RCTTQEEGKNS-----VPKRPVGP-VYRLEMPNSP-YASTDLRLSQRVHFNSPQEGYYPV 165

Query: 709 PSSYTSPMLSTSGLQDTSFGIFGGIILATVFGNQVTNIYTYPNSYHHSGNSNPRIRRHLM 888

+S LS S + D + G ++ +FG +V + + YP++Y+ +G S PR+RR +M

Sbjct: 166 SGVMSSNSLSYSAVLDPVMVMVGEMVATRLFGTRVMDRFAYPDTYNLAGTSGPRMRRRIM 225

Query: 889 QADESLSRI 915

QAD+SL RI

Sbjct: 226 QADKSLGRI 234

Score = 74 (31.1 bits), Expect = 2.4e-38, Sum P(2) = 2.4e-38

Identities = 20/53 (37%), Positives = 24/53 (45%), Frame = +3

Query: 216 MALDQYFEEAVPQINSFEDKPSLEKWKSPSDAIAD--SDRNASGGFDCNICLE 368

MALDQ FE+A + + K K P SD FDCNICL+

Sbjct: 1 MALDQSFEDAALLGELYGEGAFCFKSKKPEPITVSVPSDDTDDSNFDCNICLD 53

OLIGO [start](http://bioinfo.ut.ee/primer3-0.4.0/primer3_www_results_help.html#PRIMER_START)  [len](http://bioinfo.ut.ee/primer3-0.4.0/primer3_www_results_help.html#PRIMER_LEN)  [tm](http://bioinfo.ut.ee/primer3-0.4.0/primer3_www_results_help.html#PRIMER_TM)  [gc%](http://bioinfo.ut.ee/primer3-0.4.0/primer3_www_results_help.html#PRIMER_GC)  [any](http://bioinfo.ut.ee/primer3-0.4.0/primer3_www_results_help.html#PRIMER_ANY)  [3'](http://bioinfo.ut.ee/primer3-0.4.0/primer3_www_results_help.html#PRIMER_REPEAT) [seq](http://bioinfo.ut.ee/primer3-0.4.0/primer3_www_results_help.html#PRIMER_OLIGO_SEQ)

LEFT PRIMER 14 20 60.15 55.00 3.00 0.00 GTTATCATCCCCGTCCTCCT

RIGHT PRIMER 232 21 59.95 52.38 4.00 3.00 GCCTTCTGATCCTTGGGTTAC

SEQUENCE SIZE: 268

INCLUDED REGION SIZE: 268

PRODUCT SIZE: 219, PAIR ANY COMPL: 4.00, PAIR 3' COMPL: 0.00

1 CCAACTTATCAAAGTTATCATCCCCGTCCTCCTTATCATCCTCAACAATTTGACTCCATT

>>>>>>>>>>>>>>>>>>>>

61 CCAAGCAGTTACACTTCACCAATGCTTAGCACAAGTGGTTTACAAGATACCTCATTTGGA

121 ATCTTTGGTGGTATCATACTTGCTACGGTCTTTGGTAACCAGGTGACAAACATATATACA

181 TATCCTAATTCGTATCATCATTCAGGGAACAGTAACCCAAGGATCAGAAGGCATTTAATG

<<<<<<<<<<<<<<<<<<<<<

241 CAAGCTGATGAATCACTTAGTAGAATCT

KEYS (in order of precedence):

>>>>>> left primer

<<<<<< right primer

ADDITIONAL OLIGOS

[start](http://bioinfo.ut.ee/primer3-0.4.0/primer3_www_results_help.html#PRIMER_START)  [len](http://bioinfo.ut.ee/primer3-0.4.0/primer3_www_results_help.html#PRIMER_LEN)  [tm](http://bioinfo.ut.ee/primer3-0.4.0/primer3_www_results_help.html#PRIMER_TM)  [gc%](http://bioinfo.ut.ee/primer3-0.4.0/primer3_www_results_help.html#PRIMER_GC)  [any](http://bioinfo.ut.ee/primer3-0.4.0/primer3_www_results_help.html#PRIMER_ANY)  [3'](http://bioinfo.ut.ee/primer3-0.4.0/primer3_www_results_help.html#PRIMER_REPEAT) [seq](http://bioinfo.ut.ee/primer3-0.4.0/primer3_www_results_help.html#PRIMER_OLIGO_SEQ)

1 LEFT PRIMER 14 20 60.15 55.00 3.00 0.00 GTTATCATCCCCGTCCTCCT

RIGHT PRIMER 219 21 59.84 47.62 3.00 0.00 TGGGTTACTGTTCCCTGAATG

PRODUCT SIZE: 206, PAIR ANY COMPL: 3.00, PAIR 3' COMPL: 1.00

2 LEFT PRIMER 23 20 60.29 55.00 3.00 0.00 CCCGTCCTCCTTATCATCCT

RIGHT PRIMER 232 21 59.95 52.38 4.00 3.00 GCCTTCTGATCCTTGGGTTAC

PRODUCT SIZE: 210, PAIR ANY COMPL: 4.00, PAIR 3' COMPL: 0.00

3 LEFT PRIMER 17 20 59.61 50.00 3.00 2.00 ATCATCCCCGTCCTCCTTAT

RIGHT PRIMER 232 21 59.95 52.38 4.00 3.00 GCCTTCTGATCCTTGGGTTAC

PRODUCT SIZE: 216, PAIR ANY COMPL: 4.00, PAIR 3' COMPL: 0.00

4 LEFT PRIMER 20 20 59.61 50.00 3.00 3.00 ATCCCCGTCCTCCTTATCAT

RIGHT PRIMER 232 21 59.95 52.38 4.00 3.00 GCCTTCTGATCCTTGGGTTAC

PRODUCT SIZE: 213, PAIR ANY COMPL: 4.00, PAIR 3' COMPL: 0.00

**9 Unigene1196_All**  expansin A4 , [plant-type cell wall loosening](http://www.arabidopsis.org/servlets/TairObject?type=keyword&id=10268), [plant-type cell wall modification involved in multidimensional cell growth](http://www.arabidopsis.org/servlets/TairObject?type=keyword&id=10253), [plant-type cell wall organization](http://www.arabidopsis.org/servlets/TairObject?type=keyword&id=5349), [syncytium formation](http://www.arabidopsis.org/servlets/TairObject?type=keyword&id=7391), [unidimensional cell growth](http://www.arabidopsis.org/servlets/TairObject?type=keyword&id=10252)

72

| Unigene1196_All | 1430 | 223 | 851 | 33.36952 | 128.2572 | 1.942437 | Up |
| --- | --- | --- | --- | --- | --- | --- | --- |

24

| Unigene1196_All | 1430 | 223 | 1019 | 33.36952 | 165.7168 | 2.312118 | Up |
| --- | --- | --- | --- | --- | --- | --- | --- |

1

| Unigene1196_All | 1430 | 223 | 422 | 33.36952 | 68.53994 | 1.038414 | Up |
| --- | --- | --- | --- | --- | --- | --- | --- |

>Unigene1196_All size 1430 gap 0 0%

GAAGAGAACAAATGGATGTATTCATTGGAAATAAATCACACACTTTTCGA

ATGTGAGCTTTATATAAATTAACCAAAACACACTTCTTCCTTAGATACAT

AGTGCAAAACATAATTATATCCTAACAGAATCATACTAATTAGCCGCGGG

CTACCATTTTGCTTAGCCGCTTCAGCACTCCCTACACTCTCTTTCACTTT

GCCCCCTTTCCGGTATTACTCTGTCTAGTCTAAGTCTACCATACAAATTC

AAAAGTAGAACCTCGAGGACACACCCCCCAATAATAATATAATAATATAT

ATCACACCTACCAATCAGAAACAAAAGTCAACAACTCGTCACAAGAAGCT

GTCACTCACTCACTCAGTCACAGTCTCCAAAACATCCAGTCAAAAAGACG

AGCATCCATCTACTACGAAAATAGGCGCCAAAAGGGGTAAAACTGGAAAT

AGATAATTGGTGGGAAACAACAAAAATAAAAGGAACTTGTTCTTTTTTTT

ATATTATTATTATATATTAAAAAATATATGAAGGGGTGTCGTGAGTGGTG

TGGTGTGGTGTGGTGTGGTGTGGGATCAAACACGAAAATTCTTTCCGGTG

AAAGTTTGACCGAATTGCCAGTTGGGGGGAACAATGTTCCAAGAGGTAGA

GGTGCGGCGGTCACTACTAGTGACCCGGAAGGAGACGGCCTGGCCCACCA

AGACCGCGTTGGACTGCCAGTTCTGACCCCAGTTTCGGCTCATTGGCATC

CACCCGGTTCGGGATCCCTTAACGTAGGCGCGCACGATGTCCCCTGCACC

CGCGACGTTGCTGATCAGCACCAGGTTGAAGTAACGGAAGCCGTTGATTG

TGAACCTCATCCCACCATGCTTTCTGCATGCCACCCGGCGATATGCGACA

GGAACGATGCCGGCACGGTACTCGGCAATCTTGAGGAACATGGGCATGGC

AAGATCGAAGTGTGGACGCGGAGGATTACACCAACCACCGTTGTCGTTTG

GGAGAGCATAGTTTGGGGGACAGAAGTTAGTGGCTGTAATAAAGATGGAT

GGGCTTCCGGAGTGGCACCACTCTCTGTCGTTTGCACACTTAATCTCAAA

ACACGCACCGCAGCTCAGCCCATTGTTGAAAAGTGCGGTGCTCAATGCTG

CTGTGTTCACTCCGTACCCTTGGCTGTACAGGTTCCCATACCCACATGCT

CCCCCCATGGTGCCGGAAGCATCAGATCCTCCATAGAAGGTAGCATGAGC

ACTTTGCCAAGAACCTCCAGTGTAAACCCCTGGAATTCTTGCTTCAACCA

TCCACATTAAGGACATAAGAGACGCGAGGTAGAACACACTGGCCACACCC

ATTCCTAGCTATTTTTCTTCTGACACTTCTAAAAGAATAAGAAAATTATG

AAAGAGGAGGGAGACTAATTTGGGAATGAG

CTCATTCCCAAATTAGTCTCCCTCCTCTTTCATAATTTTCTTATTCTTTTAGAAGTGTCAGAAGAAAAATAGCTAGGAATGGGTGTGGCCAGTGTGTTCTACCTCGCGTCTCTTATGTCCTTAATGTGGATGGTTGAAGCAAGAATTCCAGGGGTTTACACTGGAGGTTCTTGGCAAAGTGCTCATGCTACCTTCTATGGAGGATCTGATGCTTCCGGCACCATGGGGGGAGCATGTGGGTATGGGAACCTGTACAGCCAAGGGTACGGAGTGAACACAGCAGCATTGAGCACCGCACTTTTCAACAATGGGCTGAGCTGCGGTGCGTGTTTTGAGATTAAGTGTGCAAACGACAGAGAGTGGTGCCACTCCGGAAGCCCATCCATCTTTATTACAGCCACTAACTTCTGTCCCCCAAACTATGCTCTCCCAAACGACAACGGTGGTTGGTGTAATCCTCCGCGTCCACACTTCGATCTTGCCATGCCCATGTTCCTCAAGATTGCCGAGTACCGTGCCGGCATCGTTCCTGTCGCATATCGCCGGGTGGCATGCAGAAAGCATGGTGGGATGAGGTTCACAATCAACGGCTTCCGTTACTTCAACCTGGTGCTGATCAGCAACGTCGCGGGTGCAGGGGACATCGTGCGCGCCTACGTTAAGGGATCCCGAACCGGGTGGATGCCAATGAGCCGAAACTGGGGTCAGAACTGGCAGTCCAACGCGGTCTTGGTGGGCCAGGCCGTCTCCTTCCGGGTCACTAGTAGTGACCGCCGCACCTCTACCTCTTGGAACATTGTTCCCCCCAACTGGCAATTCGGTCAAACTTTCACCGGAAAGAATTTTCGTGTTTGATCCCACACCACACCACACCACACCACACCACTCACGACACCCCTTCATATATTTTTTAATATATAATAATAATATAAAAAAAAGAACAAGTTCCTTTTATTTTTGTTGTTTCCCACCAATTATCTATTTCCAGTTTTACCCCTTTTGGCGCCTATTTTCGTAGTAGATGGATGCTCGTCTTTTTGACTGGATGTTTTGGAGACTGTGACTGAGTGAGTGAGTGACAGCTTCTTGTGACGAGTTGTTGACTTTTGTTTCTGATTGGTAGGTGTGATATATATTATTATATTATTATTGGGGGGTGTGTCCTCGAGGTTCTACTTTTGAATTTGTATGGTAGACTTAGACTAGACAGAGTAATACCGGAAAGGGGGCAAAGTGAAAGAGAGTGTAGGGAGTGCTGAAGCGGCTAAGCAAAATGGTAGCCCGCGGCTAATTAGTATGATTCTGTTAGGATATAATTATGTTTTGCACTATGTATCTAAGGAAGAAGTGTGTTTTGGTTAATTTATATAAAGCTCACATTCGAAAAGTGTGTGATTTATTTCCAATGAATACATCCATTTGTTCTCTTC

>**[AT2G39700](http://www.arabidopsis.org/servlets/TairObject?type=locus&name=AT2G39700).1** | Symbols: ATEXPA4, ATEXP4, ATHEXP ALPHA 1.6, EXPA4 | expansin A4

| chr2:16544246-16545434 REVERSE LENGTH=257

Length = 257

Plus Strand HSPs:

Score = 1114 (397.2 bits), Expect = 3.4e-113, P = 3.4e-113

Identities = 196/244 (80%), Positives = 214/244 (87%), Frame = +1

Query: 121 LMWMVEARIPGVYTGGSWQSAHATFYGGSDASGTMGGACGYGNLYSQGYGVNTAALSTAL 300

L + +ARIPG+Y+GG+WQ+AHATFYGGSDASGTMGGACGYGNLYSQGYG NTAALSTAL

Sbjct: 14 LFSLADARIPGIYSGGAWQNAHATFYGGSDASGTMGGACGYGNLYSQGYGTNTAALSTAL 73

Query: 301 FNNGLSCGACFEIKCANDREWCHSGSPSIFITATNFCPPNYALPNDNGGWCNPPRPHFDL 480

FNNG+SCGACFE+KCAND +WCHSGSPSI ITATNFCPPN A P+DNGGWCNPPR HFDL

Sbjct: 74 FNNGMSCGACFELKCANDPQWCHSGSPSILITATNFCPPNLAQPSDNGGWCNPPREHFDL 133

Query: 481 AMPMFLKIAEYRAGIVPVAYRRVACRKHGGMRFTINGFRYFNLVLISNVAGAGDIVRAYV 660

AMP+FLKIA+YRAGIVPV+YRRV CRK GG+RFTING RYFNLVLI+NVAGAGDIVRA V

Sbjct: 134 AMPVFLKIAQYRAGIVPVSYRRVPCRKRGGIRFTINGHRYFNLVLITNVAGAGDIVRASV 193

Query: 661 KGSRTGWMPMSRNWGQNWQSNAVLVGQAVXXXXXXXXXXXXXXWNIVPPNWQFGQTFTGK 840

KGSRTGWM +SRNWGQNWQSNAVLVGQA+ WN+VP NWQFGQTF GK

Sbjct: 194 KGSRTGWMSLSRNWGQNWQSNAVLVGQALSFRVTGSDRRTSTSWNMVPSNWQFGQTFVGK 253

Query: 841 NFRV 852

NFRV

Sbjct: 254 NFRV 257

OLIGO [start](http://bioinfo.ut.ee/primer3-0.4.0/primer3_www_results_help.html#PRIMER_START)  [len](http://bioinfo.ut.ee/primer3-0.4.0/primer3_www_results_help.html#PRIMER_LEN)  [tm](http://bioinfo.ut.ee/primer3-0.4.0/primer3_www_results_help.html#PRIMER_TM)  [gc%](http://bioinfo.ut.ee/primer3-0.4.0/primer3_www_results_help.html#PRIMER_GC)  [any](http://bioinfo.ut.ee/primer3-0.4.0/primer3_www_results_help.html#PRIMER_ANY)  [3'](http://bioinfo.ut.ee/primer3-0.4.0/primer3_www_results_help.html#PRIMER_REPEAT) [seq](http://bioinfo.ut.ee/primer3-0.4.0/primer3_www_results_help.html#PRIMER_OLIGO_SEQ)

LEFT PRIMER 77 20 60.39 55.00 2.00 2.00 CGCACCTCTACCTCTTGGAA

RIGHT PRIMER 205 20 60.99 55.00 2.00 0.00 ATGAAGGGGTGTCGTGAGTG

SEQUENCE SIZE: 235

INCLUDED REGION SIZE: 235

PRODUCT SIZE: 129, PAIR ANY COMPL: 5.00, PAIR 3' COMPL: 2.00

1 CTGGGGTCAGAACTGGCAGTCCAACGCGGTCTTGGTGGGCCAGGCCGTCTCCTTCCGGGT

61 CACTAGTAGTGACCGCCGCACCTCTACCTCTTGGAACATTGTTCCCCCCAACTGGCAATT

>>>>>>>>>>>>>>>>>>>>

121 CGGTCAAACTTTCACCGGAAAGAATTTTCGTGTTTGATCCCACACCACACCACACCACAC

181 CACACCACTCACGACACCCCTTCATATATTTTTTAATATATAATAATAATATAAA

<<<<<<<<<<<<<<<<<<<<

KEYS (in order of precedence):

>>>>>> left primer

<<<<<< right primer

ADDITIONAL OLIGOS

[start](http://bioinfo.ut.ee/primer3-0.4.0/primer3_www_results_help.html#PRIMER_START)  [len](http://bioinfo.ut.ee/primer3-0.4.0/primer3_www_results_help.html#PRIMER_LEN)  [tm](http://bioinfo.ut.ee/primer3-0.4.0/primer3_www_results_help.html#PRIMER_TM)  [gc%](http://bioinfo.ut.ee/primer3-0.4.0/primer3_www_results_help.html#PRIMER_GC)  [any](http://bioinfo.ut.ee/primer3-0.4.0/primer3_www_results_help.html#PRIMER_ANY)  [3'](http://bioinfo.ut.ee/primer3-0.4.0/primer3_www_results_help.html#PRIMER_REPEAT) [seq](http://bioinfo.ut.ee/primer3-0.4.0/primer3_www_results_help.html#PRIMER_OLIGO_SEQ)

1 LEFT PRIMER 77 20 60.39 55.00 2.00 2.00 CGCACCTCTACCTCTTGGAA

RIGHT PRIMER 196 20 61.16 55.00 2.00 0.00 TGTCGTGAGTGGTGTGGTGT

PRODUCT SIZE: 120, PAIR ANY COMPL: 6.00, PAIR 3' COMPL: 2.00

2 LEFT PRIMER 5 20 61.26 55.00 7.00 3.00 GGTCAGAACTGGCAGTCCAA

RIGHT PRIMER 165 20 59.39 45.00 8.00 0.00 GTGTGGGATCAAACACGAAA

PRODUCT SIZE: 161, PAIR ANY COMPL: 3.00, PAIR 3' COMPL: 0.00

3 LEFT PRIMER 5 20 61.26 55.00 7.00 3.00 GGTCAGAACTGGCAGTCCAA

RIGHT PRIMER 205 20 60.99 55.00 2.00 0.00 ATGAAGGGGTGTCGTGAGTG

PRODUCT SIZE: 201, PAIR ANY COMPL: 3.00, PAIR 3' COMPL: 2.00

4 LEFT PRIMER 5 20 61.26 55.00 7.00 3.00 GGTCAGAACTGGCAGTCCAA

RIGHT PRIMER 191 20 61.00 55.00 1.00 0.00 TGAGTGGTGTGGTGTGGTGT

PRODUCT SIZE: 187, PAIR ANY COMPL: 3.00, PAIR 3' COMPL: 1.00

**10。 Unigene33132_All Transcription factor bZIP48**

|  | Encodes a member of the BZIP family of transcription factors. Forms heterodimers with the related protein AtbZIP34. Binds to G-boxes in vitro and is localized to the nucleus in onion epidermal cells. |
| --- | --- |

[endoplasmic reticulum unfolded protein response](http://www.arabidopsis.org/servlets/TairObject?type=keyword&id=19007), [positive regulation of transcription, DNA-dependent](http://www.arabidopsis.org/servlets/TairObject?type=keyword&id=12513), [regulation of transcription, DNA-dependent](http://www.arabidopsis.org/servlets/TairObject?type=keyword&id=7461), [response to xenobiotic stimulus](http://www.arabidopsis.org/servlets/TairObject?type=keyword&id=7612)

72

| Unigene33132_All | 1090 | 34 | 108 | 6.674727 | 21.35431 | 1.677747 | Up |
| --- | --- | --- | --- | --- | --- | --- | --- |

24

| Unigene33132_All | 1090 | 34 | 108 | 6.674727 | 23.0423 | 1.787504 | Up |
| --- | --- | --- | --- | --- | --- | --- | --- |

1

| Unigene33132_All | 1090 | 34 | 99 | 6.674727 | 21.09483 | 1.660109 | Up |
| --- | --- | --- | --- | --- | --- | --- | --- |

>Unigene33132_All size 1090 gap 0 0%

CTTCTTCTTCTTCTTCTTGTTCTTGTATTGTGAGTGTATTGTGTTTATTATCTCAATTATGGCACAATTACCACCAAAGATCCCAAACATGTCACCAAGTTGGCCAGACTTTTCTGCTTACCAGAAAATGGCATCACATGCAAGCTTCTCACCAAACATTGGCACCAACAACACAAACCATTATCATCATCAACAGAATAATAACCCTTCTTGGGTGGATGAGTTCCTTGACTTCTCCTCCGCCAGGCGCGGTGCTCACCGCCGCTCGGTGAGCGATTCCATCACCTTCCTTGAGGCACCAATGACAATGAGGTCGGGGAATAATATTGACAATGATTTCGACAAGTTTGATGATGATCAATTGATGTCCATGTTCAACGATGAAATTTATGGGGTCAACGTGCCACCAACCTTGTCCTCATCAAACCCTTCAAGCCCTTCGGATCAGAACTTCGCCAATGATGAGAAAGAAACGAACCAGCACGAGAAGAACGAGAAGAAGGTACTGGATAAGGAGCAGAAGAAGGAGGAGGAAGAGGAGGAACAACAGCAGCAATTGAAAAATGAAGCAGATGAAGTTGAAAGCCAATGCAAACAGGAAATTACACAAGATCCAAATAACACCAATACTACTTCTACTTCTTCCAATGACAGAATTACTGACCCCAAGAGAGTCAAAAGAATCTTAGCAAATAGACAATCAGCACAAAGATCGCGAGTGAGAAAGCTGCAATACATATCAGAGCTTGAGCGAAGTGTAACTTCATTACAGGCCGAAGTTTCAGTGCTGTCTCCACGGGTTGCGTTTTTAGATCATCAACGCTTACTTCTAAATGTTGACAACAGTGCTCTCAAGCAAAGAATCGCCGCTCTTGCCCAAGACAAGATCTTCAAAGATGCTCATCAAGAGGCACTGAAGAGAGAGATAGAGAGACTAAGGCAAGTGTATCACCAACAAAACCTCAAGAAGATGGAAAATGCTGCAGGGTCACAATCACCGTCCTCATCACCAAAGCCAAGATGTGATGCTCCTCAAACTGAAAAGGAACAGCTTCTTAACGTTTGAACCCTCCACAACACAAGAATCGCC

>**[AT3G58120](http://www.arabidopsis.org/servlets/TairObject?type=locus&name=AT3G58120).1** | Symbols: ATBZIP61, BZIP61 | Basic-leucine zipper (bZIP)

transcription factor family protein | chr3:21521289-21523078

REVERSE LENGTH=329

Length = 329

Plus Strand HSPs:

Score = 782 (280.3 bits), Expect = 5.2e-78, P = 5.2e-78

Identities = 186/348 (53%), Positives = 205/348 (58%), Frame = +2

Query: 59 MAQLPPKIPNMS-PSWPDFSAYQKMASHASFSPNIGXXXXXXXXXXXXXXPSWVDEFLDF 235

MAQLPPKIP M+ P+WPDFS+ QK+ S A+ PSW+DEFLDF

Sbjct: 1 MAQLPPKIPTMTTPNWPDFSS-QKLPSIAA------TAAAAATAGPQQQNPSWMDEFLDF 53

Query: 236 SSARRGAHRRSVSDSITFLEAPMTMRSGXXXXXXXXXXXXXQLMSMFNDEIYGVN----- 400

S+ RRG HRRS+SDSI FLE P S Q MSMFND+++ N

Sbjct: 54 SATRRGTHRRSISDSIAFLEPP----SSGVGNHHFDRFDDEQFMSMFNDDVHNNNHNHHH 109

Query: 401 -------VXXXXXXXXXXXXXDQNFANDEKETNQHEXXXXXXXXXXXXXXXXXXXXXXXX 559

V D N +D+ N E

Sbjct: 110 HHSINGNVGPTRSSSNTSTPSDHNSLSDDD--NNKEAPPSDHDHHMDNNVANQNNAAGNN 167

Query: 560 XXXADEVESQCKQEITQDPXXXXXXXXXXXDRITDPKRVKRILANRQSAQRSRVRKLQYI 739

+DEV+SQCK E P +RI DPKRVKRILANRQSAQRSRVRKLQYI

Sbjct: 168 YNESDEVQSQCKTEPQDGPSANQNSGGSSGNRIHDPKRVKRILANRQSAQRSRVRKLQYI 227

Query: 740 SELERSVTSLQAEVSVLSPRVAFLDHQRLLLNVDNSALKQRIAALAQDKIFKDAHQEALK 919

SELERSVTSLQ EVSVLSPRVAFLDHQRLLLNVDNSA+KQRIAALAQDKIFKDAHQEALK

Sbjct: 228 SELERSVTSLQTEVSVLSPRVAFLDHQRLLLNVDNSAIKQRIAALAQDKIFKDAHQEALK 287

Query: 920 REIERLRQVYHQQNLKKMENAAGSQSPSSSPKPRCDAPQTEKEQLLNV 1063

REIERLRQVYHQQ+LKKMEN QSP+ P EKEQLLNV

Sbjct: 288 REIERLRQVYHQQSLKKMENNVSDQSPADIK------PSVEKEQLLNV 329

OLIGO [start](http://bioinfo.ut.ee/primer3-0.4.0/primer3_www_results_help.html#PRIMER_START)  [len](http://bioinfo.ut.ee/primer3-0.4.0/primer3_www_results_help.html#PRIMER_LEN)  [tm](http://bioinfo.ut.ee/primer3-0.4.0/primer3_www_results_help.html#PRIMER_TM)  [gc%](http://bioinfo.ut.ee/primer3-0.4.0/primer3_www_results_help.html#PRIMER_GC)  [any](http://bioinfo.ut.ee/primer3-0.4.0/primer3_www_results_help.html#PRIMER_ANY)  [3'](http://bioinfo.ut.ee/primer3-0.4.0/primer3_www_results_help.html#PRIMER_REPEAT) [seq](http://bioinfo.ut.ee/primer3-0.4.0/primer3_www_results_help.html#PRIMER_OLIGO_SEQ)

LEFT PRIMER 21 20 59.30 45.00 5.00 2.00 TGCTCTCAAGCAAAGAATCG

RIGHT PRIMER 255 20 59.98 45.00 3.00 2.00 TGTGTTGTGGAGGGTTCAAA

SEQUENCE SIZE: 260

INCLUDED REGION SIZE: 260

PRODUCT SIZE: 235, PAIR ANY COMPL: 4.00, PAIR 3' COMPL: 0.00

1 CTTCTAAATGTTGACAACAGTGCTCTCAAGCAAAGAATCGCCGCTCTTGCCCAAGACAAG

>>>>>>>>>>>>>>>>>>>>

61 ATCTTCAAAGATGCTCATCAAGAGGCACTGAAGAGAGAGATAGAGAGACTAAGGCAAGTG

121 TATCACCAACAAAACCTCAAGAAGATGGAAAATGCTGCAGGGTCACAATCACCGTCCTCA

181 TCACCAAAGCCAAGATGTGATGCTCCTCAAACTGAAAAGGAACAGCTTCTTAACGTTTGA

<<<<<

241 ACCCTCCACAACACAAGAAT

<<<<<<<<<<<<<<<

KEYS (in order of precedence):

>>>>>> left primer

<<<<<< right primer

ADDITIONAL OLIGOS

[start](http://bioinfo.ut.ee/primer3-0.4.0/primer3_www_results_help.html#PRIMER_START)  [len](http://bioinfo.ut.ee/primer3-0.4.0/primer3_www_results_help.html#PRIMER_LEN)  [tm](http://bioinfo.ut.ee/primer3-0.4.0/primer3_www_results_help.html#PRIMER_TM)  [gc%](http://bioinfo.ut.ee/primer3-0.4.0/primer3_www_results_help.html#PRIMER_GC)  [any](http://bioinfo.ut.ee/primer3-0.4.0/primer3_www_results_help.html#PRIMER_ANY)  [3'](http://bioinfo.ut.ee/primer3-0.4.0/primer3_www_results_help.html#PRIMER_REPEAT) [seq](http://bioinfo.ut.ee/primer3-0.4.0/primer3_www_results_help.html#PRIMER_OLIGO_SEQ)

1 LEFT PRIMER 21 20 59.30 45.00 5.00 2.00 TGCTCTCAAGCAAAGAATCG

RIGHT PRIMER 256 20 59.98 45.00 3.00 3.00 TTGTGTTGTGGAGGGTTCAA

PRODUCT SIZE: 236, PAIR ANY COMPL: 4.00, PAIR 3' COMPL: 1.00

2 LEFT PRIMER 72 20 60.71 50.00 5.00 3.00 TGCTCATCAAGAGGCACTGA

RIGHT PRIMER 255 20 59.98 45.00 3.00 2.00 TGTGTTGTGGAGGGTTCAAA

PRODUCT SIZE: 184, PAIR ANY COMPL: 4.00, PAIR 3' COMPL: 1.00

3 LEFT PRIMER 72 20 60.71 50.00 5.00 3.00 TGCTCATCAAGAGGCACTGA

RIGHT PRIMER 256 20 59.98 45.00 3.00 3.00 TTGTGTTGTGGAGGGTTCAA

PRODUCT SIZE: 185, PAIR ANY COMPL: 4.00, PAIR 3' COMPL: 2.00

4 LEFT PRIMER 21 20 59.30 45.00 5.00 2.00 TGCTCTCAAGCAAAGAATCG

RIGHT PRIMER 209 20 59.79 50.00 4.00 0.00 TGAGGAGCATCACATCTTGG

PRODUCT SIZE: 189, PAIR ANY COMPL: 6.00, PAIR 3' COMPL: 2.00

**11. Unigene16704_All**， plant U-box 26 (PUB26); FUNCTIONS IN: ubiquitin-protein ligase activity, binding; INVOLVED IN: protein ubiquitination; LOCATED IN: ubiquitin ligase complex;

72

| Unigene16704_All | 1504 | 143 | 433 | 20.34554 | 62.04808 | 1.608674 | Up |
| --- | --- | --- | --- | --- | --- | --- | --- |

24

| Unigene16704_All | 1504 | 143 | 435 | 20.34554 | 67.26202 | 1.725079 | Up |
| --- | --- | --- | --- | --- | --- | --- | --- |

1

| Unigene16704_All | 1504 | 143 | 312 | 20.34554 | 48.18081 | 1.243746 | Up |
| --- | --- | --- | --- | --- | --- | --- | --- |

>Unigene16704_All size 1504 gap 0 0%

GCTGGCTGCACGCAAAATCGTCAGAATTTCCAATAGAATCTTGTGGCCAC

GAATCCCGAAGAAGCTTCAGCAACAACTGCGCCTTCCTCTTCGCTCTCTC

CGTGCAATCACTCTGCACAAGAAGCAGCAGCTGAGTCAGCACGCCCGCCG

CCACCGCGTCCCGCTGGCACATCTCCGATTCCGAGCACAGAGCCAGCAGC

GCTCCGGTGGCGTACTCCGTCGCACGATCCGAAATCTTCAATATTATCTT

CACCAACAGCGGCACCGTCAGCGCGTGTTCCGCGAACGCGGCGCGTCCAT

CAGGAATACGGCAAAGAAGCTCCACCGTCGCCAGCGCCCTCTCAGCATCG

CACTTCTCGAAATCAGCGAGCCGGTCGACGATCACCGCCGGAGCTCCGGC

AACCACCGCCTTGTGCCGCGTCTGTTTCACCAAACACAAAGCGAACAACG

CCTTGATCCCGATCTTAAGAGCGCGAGGAAACGAGATCGGGTTCTTCAGT

ATGTCAATCACGCCGTCAAAGATTCCGTCGACATTGCTGATCTGAGACCG

TAGCTCCTGCGAACGAGTCCCCGCCGCGACGATCTCAATCATCGCCGCGG

CGTTAACTCGAACATCGAACGAACCATGAAACAGCAACCGCGATAGATAA

CTAACCTTATCAGAATCAGTCGCGACGAAACCGCATTCCGTTTCGCTCAA

CGGGAATAACACGAGTAACGCGAGTGACTCGTGAGTCAACTCGTCGGTTA

TATTCGTGAAATTGAAAAGTATATTTAGGAGAACAAACCGTACGTTTTGT

GAAGCGATAAGAGAACGGTTTTTATCTGAATCGCGAGCGAGAGTTCTGAG

TCTACGTAGCGAAGAAACACGAATATGAGAGGGTGTGGTTTGGGAAGAGC

ATTGGTTAAGAAGGGAACGAACTAAGGCTGGGTCTGCGGGCTGTTTGGGT

GTTGGAATTCTTTCAACGCCGAAAGAGCGGTTAGCGACGCACCAATCTTG

GATCAGACGGCGGAGGGTGTGGTTAGGGATGAAGGTGAAATCGGTTAGAG

GGGCTCTGGTGACCGGGCACGTGGTGTTGCCGGTTGCAACCCATGACTCT

ATACTGTGACGGTCGTAGGTTTGACCGGTGCAGACTGTAACCGGGTCACG

CATGAGTTCAAGGGAGATTGGGCACCTGAAGTGGTATGGAATCTGGACCC

CTAAGTCCAAAGGTTCTAAACTGCCAGGCATTGTAATGAAATGAATGAAA

GAGATGTTTGGGAGAAAGAGGAAGAGTGTGTTTGTGTGTTTATATGAGTG

GCAGTGGTAGGAGTACGAGAAATAAAGATAATTTGGTTTCTCTTTATATT

ATGAGATGGAACAGAGGAACATCCCCCACAGCAACAGAGAAGGACTGATC

CTCACTCTCTGCAATATGCCCCTGCTCCTTATCTGACTTGTGCTGTATTT

CTCCACCCTCACCACACGTGTGGTGGCATTCTAACTTGGAATCTAACCCT

GGTT

AACCAGGGTTAGATTCCAAGTTAGAATGCCACCACACGTGTGGTGAGGGTGGAGAAATACAGCACAAGTCAGATAAGGAGCAGGGGCATATTGCAGAGAGTGAGGATCAGTCCTTCTCTGTTGCTGTGGGGGATGTTCCTCTGTTCCATCTCATAATATAAAGAGAAACCAAATTATCTTTATTTCTCGTACTCCTACCACTGCCACTCATATAAACACACAAACACACTCTTCCTCTTTCTCCCAAACATCTCTTTCATTCATTTCATTACAATGCCTGGCAGTTTAGAACCTTTGGACTTAGGGGTCCAGATTCCATACCACTTCAGGTGCCCAATCTCCCTTGAACTCATGCGTGACCCGGTTACAGTCTGCACCGGTCAAACCTACGACCGTCACAGTATAGAGTCATGGGTTGCAACCGGCAACACCACGTGCCCGGTCACCAGAGCCCCTCTAACCGATTTCACCTTCATCCCTAACCACACCCTCCGCCGTCTGATCCAAGATTGGTGCGTCGCTAACCGCTCTTTCGGCGTTGAAAGAATTCCAACACCCAAACAGCCCGCAGACCCAGCCTTAGTTCGTTCCCTTCTTAACCAATGCTCTTCCCAAACCACACCCTCTCATATTCGTGTTTCTTCGCTACGTAGACTCAGAACTCTCGCTCGCGATTCAGATAAAAACCGTTCTCTTATCGCTTCACAAAACGTACGGTTTGTTCTCCTAAATATACTTTTCAATTTCACGAATATAACCGACGAGTTGACTCACGAGTCACTCGCGTTACTCGTGTTATTCCCGTTGAGCGAAACGGAATGCGGTTTCGTCGCGACTGATTCTGATAAGGTTAGTTATCTATCGCGGTTGCTGTTTCATGGTTCGTTCGATGTTCGAGTTAACGCCGCGGCGATGATTGAGATCGTCGCGGCGGGGACTCGTTCGCAGGAGCTACGGTCTCAGATCAGCAATGTCGACGGAATCTTTGACGGCGTGATTGACATACTGAAGAACCCGATCTCGTTTCCTCGCGCTCTTAAGATCGGGATCAAGGCGTTGTTCGCTTTGTGTTTGGTGAAACAGACGCGGCACAAGGCGGTGGTTGCCGGAGCTCCGGCGGTGATCGTCGACCGGCTCGCTGATTTCGAGAAGTGCGATGCTGAGAGGGCGCTGGCGACGGTGGAGCTTCTTTGCCGTATTCCTGATGGACGCGCCGCGTTCGCGGAACACGCGCTGACGGTGCCGCTGTTGGTGAAGATAATATTGAAGATTTCGGATCGTGCGACGGAGTACGCCACCGGAGCGCTGCTGGCTCTGTGCTCGGAATCGGAGATGTGCCAGCGGGACGCGGTGGCGGCGGGCGTGCTGACTCAGCTGCTGCTTCTTGTGCAGAGTGATTGCACGGAGAGAGCGAAGAGGAAGGCGCAGTTGTTGCTGAAGCTTCTTCGGGATTCGTGGCCACAAGATTCTATTGGAAATTCTGACGATTTTGCGTGCAGCCAGC

>**[AT1G49780](http://www.arabidopsis.org/servlets/TairObject?type=locus&name=AT1G49780).1** | Symbols: PUB26 | plant U-box 26 | chr1:18429024-18430289 REVERSE

LENGTH=421

Length = 421

Plus Strand HSPs:

Score = 1381 (491.2 bits), Expect = 1.7e-141, P = 1.7e-141

Identities = 272/418 (65%), Positives = 329/418 (78%), Frame = +1

Query: 274 MPGSLEPLDLGVQIPYHFRCPISLELMRDPVTVCTGQTYDRHSIESWVATGNTTCPVTRA 453

MPG+LEPLDLG+QIPYHFRCPISL+LM DPVT+ TGQTYDR SI+SW+A GNTTCPVTR

Sbjct: 1 MPGNLEPLDLGIQIPYHFRCPISLDLMSDPVTISTGQTYDRTSIDSWIAMGNTTCPVTRV 60

Query: 454 PLTDFTFIPNHTLRRLIQDWCVANRSFGVERIPTPKQPADPALVRSLLNQCSSQTTPSHI 633

L+DFT IPNHTLRRLIQ+WCVANRS GVERIPTPKQPADP VRSLL+Q S+ T +H+

Sbjct: 61 ALSDFTLIPNHTLRRLIQEWCVANRSNGVERIPTPKQPADPISVRSLLSQASA-ITGTHV 119

Query: 634 ----RVSSLRRLRTLARDSDKNRSLIASQNVRFVLLNILF---NFTNITDELTHESLALL 792

R +++RRLR LARDS+KNR LIA N R +L+ ILF T+++ EL ESLALL

Sbjct: 120 SVRSRAAAIRRLRGLARDSEKNRVLIAGHNAREILVRILFADIETTSLSSELVSESLALL 179

Query: 793 VLFPLSETECGFVATDSDKVSYLSRLLFHGSFDVRVNAAAMIEIVAAGTRSQELRSQISN 972

VL ++ETEC VA+D +V +++RLLF S ++RVNAAA+IE+V G +S +L+ IS

Sbjct: 180 VLLHMTETECEAVASDPSRVGFMTRLLFDSSIEIRVNAAALIEMVLTGAKSMDLKLIISG 239

Query: 973 VDGIFDGVIDILKNPISFPRALKIGIKALFALCLVKQTRHKAVVAGAPAVIVDRLA-DFE 1149

D IF+GV+D+LKNPIS RALKIGIKA+FALCLVKQTRH A+ AGAP +++DRLA DF+

Sbjct: 240 SDSIFEGVLDLLKNPISSRRALKIGIKAIFALCLVKQTRHLAISAGAPGILIDRLAADFD 299

Query: 1150 KCDAERALATVELLCRIPDGRAAFAEHALTVPLLVKIILKISDRATEYATGALLALCSES 1329

+CD ER LATVELLCR+P+G AAF EHALTVPL+VK IL++SDRATEYA GALLALC+

Sbjct: 300 RCDTERGLATVELLCRLPEGCAAFGEHALTVPLMVKTILRVSDRATEYAAGALLALCTAE 359

Query: 1330 EMCQRDAVAAGVLTQLLLLVQSDCTEXXXXXXXXXXXXXXDSWPQDSIGNSDDFACSQ 1503

E C+ +A AAG++TQLLLLVQSDCTE DSWP DS +SDDF S+

Sbjct: 360 ERCRDEAAAAGLVTQLLLLVQSDCTERAKRKAQMLLKLLRDSWPDDSTVHSDDFNRSE 417

OLIGO [start](http://bioinfo.ut.ee/primer3-0.4.0/primer3_www_results_help.html#PRIMER_START)  [len](http://bioinfo.ut.ee/primer3-0.4.0/primer3_www_results_help.html#PRIMER_LEN)  [tm](http://bioinfo.ut.ee/primer3-0.4.0/primer3_www_results_help.html#PRIMER_TM)  [gc%](http://bioinfo.ut.ee/primer3-0.4.0/primer3_www_results_help.html#PRIMER_GC)  [any](http://bioinfo.ut.ee/primer3-0.4.0/primer3_www_results_help.html#PRIMER_ANY)  [3'](http://bioinfo.ut.ee/primer3-0.4.0/primer3_www_results_help.html#PRIMER_REPEAT) [seq](http://bioinfo.ut.ee/primer3-0.4.0/primer3_www_results_help.html#PRIMER_OLIGO_SEQ)

LEFT PRIMER 6 20 61.17 50.00 3.00 1.00 GCCGCTGTTGGTGAAGATAA

RIGHT PRIMER 159 20 59.93 50.00 4.00 1.00 ACTCTGCACAAGAAGCAGCA

SEQUENCE SIZE: 251

INCLUDED REGION SIZE: 251

PRODUCT SIZE: 154, PAIR ANY COMPL: 7.00, PAIR 3' COMPL: 3.00

1 ACGGTGCCGCTGTTGGTGAAGATAATATTGAAGATTTCGGATCGTGCGACGGAGTACGCC

>>>>>>>>>>>>>>>>>>>>

61 ACCGGAGCGCTGCTGGCTCTGTGCTCGGAATCGGAGATGTGCCAGCGGGACGCGGTGGCG

121 GCGGGCGTGCTGACTCAGCTGCTGCTTCTTGTGCAGAGTGATTGCACGGAGAGAGCGAAG

<<<<<<<<<<<<<<<<<<<<

181 AGGAAGGCGCAGTTGTTGCTGAAGCTTCTTCGGGATTCGTGGCCACAAGATTCTATTGGA

241 AATTCTGACGA

KEYS (in order of precedence):

>>>>>> left primer

<<<<<< right primer

ADDITIONAL OLIGOS

[start](http://bioinfo.ut.ee/primer3-0.4.0/primer3_www_results_help.html#PRIMER_START)  [len](http://bioinfo.ut.ee/primer3-0.4.0/primer3_www_results_help.html#PRIMER_LEN)  [tm](http://bioinfo.ut.ee/primer3-0.4.0/primer3_www_results_help.html#PRIMER_TM)  [gc%](http://bioinfo.ut.ee/primer3-0.4.0/primer3_www_results_help.html#PRIMER_GC)  [any](http://bioinfo.ut.ee/primer3-0.4.0/primer3_www_results_help.html#PRIMER_ANY)  [3'](http://bioinfo.ut.ee/primer3-0.4.0/primer3_www_results_help.html#PRIMER_REPEAT) [seq](http://bioinfo.ut.ee/primer3-0.4.0/primer3_www_results_help.html#PRIMER_OLIGO_SEQ)

1 LEFT PRIMER 6 20 61.17 50.00 3.00 1.00 GCCGCTGTTGGTGAAGATAA

RIGHT PRIMER 153 20 60.07 55.00 7.00 3.00 CACAAGAAGCAGCAGCTGAG

PRODUCT SIZE: 148, PAIR ANY COMPL: 5.00, PAIR 3' COMPL: 0.00

2 LEFT PRIMER 6 20 61.17 50.00 3.00 1.00 GCCGCTGTTGGTGAAGATAA

RIGHT PRIMER 158 20 60.07 55.00 4.00 2.00 CTCTGCACAAGAAGCAGCAG

PRODUCT SIZE: 153, PAIR ANY COMPL: 7.00, PAIR 3' COMPL: 3.00

3 LEFT PRIMER 6 20 61.17 50.00 3.00 1.00 GCCGCTGTTGGTGAAGATAA

RIGHT PRIMER 173 20 59.57 55.00 4.00 1.00 CTCTCCGTGCAATCACTCTG

PRODUCT SIZE: 168, PAIR ANY COMPL: 4.00, PAIR 3' COMPL: 1.00

4 LEFT PRIMER 6 20 61.17 50.00 3.00 1.00 GCCGCTGTTGGTGAAGATAA

RIGHT PRIMER 176 20 60.56 55.00 4.00 1.00 GCTCTCTCCGTGCAATCACT

PRODUCT SIZE: 171, PAIR ANY COMPL: 4.00, PAIR 3' COMPL: 1.00

**12 CL6565.Contig2_All CIPK25**

CBL-INTERACTING PROTEIN KINASE 25, CIPK25, SNF1-RELATED PROTEIN KINASE 3.25, SNRK3.25

[defense response to fungus](http://www.arabidopsis.org/servlets/TairObject?type=keyword&id=18013), [protein phosphorylation](http://www.arabidopsis.org/servlets/TairObject?type=keyword&id=6897), [signal transduction](http://www.arabidopsis.org/servlets/TairObject?type=keyword&id=7243)

72

| CL6565.Contig2_All | 2434 | 1571 | 4352 | 138.1137 | 385.3511 | 1.480317 | Up |
| --- | --- | --- | --- | --- | --- | --- | --- |

24

| CL6565.Contig2_All | 2434 | 1571 | 3219 | 138.1137 | 307.5593 | 1.155009 | Up |
| --- | --- | --- | --- | --- | --- | --- | --- |

1

| CL6565.Contig2_All | 2434 | 1571 | 2721 | 138.1137 | 259.6422 | 0.910669 | Up |
| --- | --- | --- | --- | --- | --- | --- | --- |

>CL6565.Contig2_All size 2434 gap 0 0%

GGTCATTTGAAGGGCACTATCATAAAACTGGCCAAAAAACAATTAGGGCTGTGTTTGTGTTTGCTTCTAGGTTCAAAACCACATAAAATTTAACTTTCACATTAAATGATAAAAGAATAACAATTATGTCACTCCATGACTCCAAACTTCCTTTTGACACCCCCACCTCCGCTCCCCTCCCCTCCCCTCCCCCAACCACCGGTGTCGATGTAAACCCAAAGAAAATCGTGTTTTAACACCAAAAAAAAAAAATATTGGGGAAATGGATCCAACAGAACTTAGTTGAAGACCCGAAATAGCATAATGAAGTATAGTCTGTTTCACATATATGGGCAGTGGCAGAGACGATTCTCCTTGTTATTATATCTCCAAACTCCAAATATGACAGGTAACCTAAATCTAAATCTAAATGAATGACAAGCATAGAAACTAATAAAGAGACACTCCTTAATTTTGCATACAACTGCTTTTTCATTTCAGAATCCATTGCATATATATGCTATTGCTGGCAAATATTGTCACCCTGCCATGCCCACACAATGTCTTTGAGTGATGGTCTCACTTGGTCCTCACAGAATTTAATATACTCCAACGTGTCACCGGCAGACTTAGTAAACTCCACCACCGCCACCTCCGGCGCCACCTCAAACACCTCCACCGTCATTGCCAGCTTCCCCTTCCTTCCTTCAGTGCTCCCTTGCATCCTCACCGCGAACTCCTTCTTCCCCGTTATCCTAAAATTAAGCTTCTTAGCCACCCCTTCCAGTTTCGCCAACACCGCCGAAGCAGAAAACTTGGAAATAAACATCGACGGCGACCTCTTCCTCGTCTCAAACAAGCTCCTAAGATCAAAACCATGCGAAAAAGACGAAATAATCTCAAATGCATTGTAAAAAGGACGAGCAGGCTTTCCCATTTTGCCCTCTTGAACAACGCTGTTGTTGTTGTTATCAGATTCATAATTGAAATCGATGTTGTCTTCCACAGCCGTTTCCTTCATTGAAAACGCAAAGGGTCTAACAAACCCAACTTGAAACCAAGGATCCTTGGTGATATCATCAATGGAGTACCTCTTCTCAGGATCAACAACAAGCAAATTGGAGATGAGTTTCTTAGCCTCAGGTGAGATCCAATCTGGAAGAGCATAATCAGCTTTGAATGATTTCCTATAAATCCTTATGACATTCTCACCTTGAAACGGAAGATACCCTGAAAGCAAAGCATAGAGGATAACCCCACAAGACCATATATCAGCTTTAGATCCATCGTACCCTTTTTTCTTCAACACCTCAGGTGCCACGTAAGCCGGCGTCCCACACGGCGTTTCCAGCATTCCGTCCGATCTACGCTGTTCCGGCAAAGCGGAGAGCCCGAAATCCGAAACCTTGAGGTCTTCGTTTTCGTCCAGAAGCAAATTCTCGGGTTTTAAGTCACGGTGGGTGACGCCGCGGCTGTGGCAGAAATCGACGGCGCTGATGAGTTGTTGGAAGTACTTTCTGGCTATGTCTTCTTTCATCTTTCCCTTTGTGACCTTGGCGAAGAGTTCGCCTCCTTTGACGTATTCGACGACCATGAAGATTTTAGCTTTTGTGGCCATGACTTCTTTGAGCTGCACGATGTGTGGGTGGCGAACGAGGTGCATCACCGACACCTCGCGTTTGATCTGTTTGACGAGACTCTCTTTCTTCAGCCTCTCTTTCTTGATGACCTTCATGGCTACGCTTTCGTTGGTGACGAGGTTTCTGCCGTGGTAGACCTTGGCGAAGTTTCCTTGGCCGAGTAGCTTCCCCATCTCGTACTTGTTGAAGAGGATGTTCCTGTTGGATTGTTGTTCGGTTTTAGGATCCATTGGAGAAAAAAGGGGGGGGAAGAGGCGGTGGTAGTGAGATTTTGATGTTGTGTGTGTGTGTATGCTGCTTATATATGCTGCTACAACAACAACAGGTTGTTCAATTGGAGGCGGTTGTGGATTGGAAGGGATGGGAGAAACCCAGATGGAAAAGGTGGATCCTTTTCAGGAAGATGGTGTCGAAGGGACACACGACATTTTGAAGGAAGCATTTAGAGGGATTGAAAATCTTCATGGTGGTGCTGCAGCAGCAGCAGCATACAGAGGAAGAATGAAAGGATGGATGTGAGATGAGTAGCGTAGGTTTGTTCAAGAGAGGGTTATAATATATACTATGATATGATGATGAGTAGAAGATGGATGGATGGATGGATGGGGTTGTGCCAATTTGGGGTGGTTATACCAAAAATAGGAGAGGAGAAGGGGAAAGGGATAAAGAAGGAGACGTCATGCTGAATAGGAATGGGGATAAGAGAGAGAGAAAAACAGAGTCTAGAGAAGGCGTTGGATTTGGAGACCACGGTTTTGAGTTTATACACGACACGAACAAATAATCGATCAATTAAACCATGCTGTTGGGCTGAC

GTCAGCCCAACAGCATGGTTTAATTGATCGATTATTTGTTCGTGTCGTGTATAAACTCAAAACCGTGGTCTCCAAATCCAACGCCTTCTCTAGACTCTGTTTTTCTCTCTCTCTTATCCCCATTCCTATTCAGCATGACGTCTCCTTCTTTATCCCTTTCCCCTTCTCCTCTCCTATTTTTGGTATAACCACCCCAAATTGGCACAACCCCATCCATCCATCCATCCATCTTCTACTCATCATCATATCATAGTATATATTATAACCCTCTCTTGAACAAACCTACGCTACTCATCTCACATCCATCCTTTCATTCTTCCTCTGTATGCTGCTGCTGCTGCAGCACCACCATGAAGATTTTCAATCCCTCTAAATGCTTCCTTCAAAATGTCGTGTGTCCCTTCGACACCATCTTCCTGAAAAGGATCCACCTTTTCCATCTGGGTTTCTCCCATCCCTTCCAATCCACAACCGCCTCCAATTGAACAACCTGTTGTTGTTGTAGCAGCATATATAAGCAGCATACACACACACACAACATCAAAATCTCACTACCACCGCCTCTTCCCCCCCCTTTTTTCTCCAATGGATCCTAAAACCGAACAACAATCCAACAGGAACATCCTCTTCAACAAGTACGAGATGGGGAAGCTACTCGGCCAAGGAAACTTCGCCAAGGTCTACCACGGCAGAAACCTCGTCACCAACGAAAGCGTAGCCATGAAGGTCATCAAGAAAGAGAGGCTGAAGAAAGAGAGTCTCGTCAAACAGATCAAACGCGAGGTGTCGGTGATGCACCTCGTTCGCCACCCACACATCGTGCAGCTCAAAGAAGTCATGGCCACAAAAGCTAAAATCTTCATGGTCGTCGAATACGTCAAAGGAGGCGAACTCTTCGCCAAGGTCACAAAGGGAAAGATGAAAGAAGACATAGCCAGAAAGTACTTCCAACAACTCATCAGCGCCGTCGATTTCTGCCACAGCCGCGGCGTCACCCACCGTGACTTAAAACCCGAGAATTTGCTTCTGGACGAAAACGAAGACCTCAAGGTTTCGGATTTCGGGCTCTCCGCTTTGCCGGAACAGCGTAGATCGGACGGAATGCTGGAAACGCCGTGTGGGACGCCGGCTTACGTGGCACCTGAGGTGTTGAAGAAAAAAGGGTACGATGGATCTAAAGCTGATATATGGTCTTGTGGGGTTATCCTCTATGCTTTGCTTTCAGGGTATCTTCCGTTTCAAGGTGAGAATGTCATAAGGATTTATAGGAAATCATTCAAAGCTGATTATGCTCTTCCAGATTGGATCTCACCTGAGGCTAAGAAACTCATCTCCAATTTGCTTGTTGTTGATCCTGAGAAGAGGTACTCCATTGATGATATCACCAAGGATCCTTGGTTTCAAGTTGGGTTTGTTAGACCCTTTGCGTTTTCAATGAAGGAAACGGCTGTGGAAGACAACATCGATTTCAATTATGAATCTGATAACAACAACAACAGCGTTGTTCAAGAGGGCAAAATGGGAAAGCCTGCTCGTCCTTTTTACAATGCATTTGAGATTATTTCGTCTTTTTCGCATGGTTTTGATCTTAGGAGCTTGTTTGAGACGAGGAAGAGGTCGCCGTCGATGTTTATTTCCAAGTTTTCTGCTTCGGCGGTGTTGGCGAAACTGGAAGGGGTGGCTAAGAAGCTTAATTTTAGGATAACGGGGAAGAAGGAGTTCGCGGTGAGGATGCAAGGGAGCACTGAAGGAAGGAAGGGGAAGCTGGCAATGACGGTGGAGGTGTTTGAGGTGGCGCCGGAGGTGGCGGTGGTGGAGTTTACTAAGTCTGCCGGTGACACGTTGGAGTATATTAAATTCTGTGAGGACCAAGTGAGACCATCACTCAAAGACATTGTGTGGGCATGGCAGGGTGACAATATTTGCCAGCAATAGCATATATATGCAATGGATTCTGAAATGAAAAAGCAGTTGTATGCAAAATTAAGGAGTGTCTCTTTATTAGTTTCTATGCTTGTCATTCATTTAGATTTAGATTTAGGTTACCTGTCATATTTGGAGTTTGGAGATATAATAACAAGGAGAATCGTCTCTGCCACTGCCCATATATGTGAAACAGACTATACTTCATTATGCTATTTCGGGTCTTCAACTAAGTTCTGTTGGATCCATTTCCCCAATATTTTTTTTTTTTGGTGTTAAAACACGATTTTCTTTGGGTTTACATCGACACCGGTGGTTGGGGGAGGGGAGGGGAGGGGAGCGGAGGTGGGGGTGTCAAAAGGAAGTTTGGAGTCATGGAGTGACATAATTGTTATTCTTTTATCATTTAATGTGAAAGTTAAATTTTATGTGGTTTTGAACCTAGAAGCAAACACAAACACAGCCCTAATTGTTTTTTGGCCAGTTTTATGATAGTGCCCTTCAAATGACC

>**[AT5G25110](http://www.arabidopsis.org/servlets/TairObject?type=locus&name=AT5G25110).1** | Symbols: CIPK25, SnRK3.25 | CBL-interacting protein kinase 25 |

chr5:8657740-8659206 REVERSE LENGTH=488

Length = 488

Plus Strand HSPs:

Score = 1287 (458.1 bits), Expect = 1.6e-131, P = 1.6e-131

Identities = 242/441 (54%), Positives = 316/441 (71%), Frame = +1

Query: 601 EQQSNRNILFNKYEMGKLLGQGNFAKVYHGRNLVTNESVAMKVIKKERLKKESLVKQIKR 780

E+Q +LF KYEMG+LLG+G F KVY+G+ + T ESVA+K+I K+++K+E +++QIKR

Sbjct: 31 EEQQQLRVLFAKYEMGRLLGKGTFGKVYYGKEITTGESVAIKIINKDQVKREGMMEQIKR 90

Query: 781 EVSVMHLVRHPHIVQLKEVMATKAKIFMVVEYVKGGELFAKVTKGKMKEDIARKYFQQLI 960

E+S+M LVRHP+IV+LKEVMATK KIF ++EYVKGGELF+K+ KGK+KED ARKYFQQLI

Sbjct: 91 EISIMRLVRHPNIVELKEVMATKTKIFFIMEYVKGGELFSKIVKGKLKEDSARKYFQQLI 150

Query: 961 SAVDFCHSRGVTHRXXXXXXXXXXXXXXXXVSDFGLSALPEQRRSDGMLETPCGTPAYVA 1140

SAVDFCHSRGV+HR VSDFGLSALPEQ DG+L T CGTPAYVA

Sbjct: 151 SAVDFCHSRGVSHRDLKPENLLVDENGDLKVSDFGLSALPEQILQDGLLHTQCGTPAYVA 210

Query: 1141 PEVLKKKGYDGSKADIWSCGVILYALLSGYLPFQGENVIRIYRKSFKADYALPDWISPEA 1320

PEVL+KKGYDG+K DIWSCG+ILY LL+G+LPFQ EN++++YRK FK+++ P W SPE+

Sbjct: 211 PEVLRKKGYDGAKGDIWSCGIILYVLLAGFLPFQDENLMKMYRKIFKSEFEYPPWFSPES 270

Query: 1321 KKLISNLLVVDPEKRYSIDDITKDPWFQVGFVRPFAFSMKETAVEDNIDFNYESDNNNNS 1500

K+LIS LLVVDP KR SI I + PWF+ P F + E ++ N E + +

Sbjct: 271 KRLISKLLVVDPNKRISIPAIMRTPWFRKNINSPIEFKIDELEIQ-----NVEDETPTTT 325

Query: 1501 VVQEGKMGKPARP-FYNAFEIISSFSHGFDLRSLFETRKRSPSMFISKFSASAVLAKLEG 1677

P P F+NAFE ISS S GFDL SLFE++++ SMF S++SAS ++ KLEG

Sbjct: 326 ATTATTTTTPVSPKFFNAFEFISSMSSGFDLSSLFESKRKLRSMFTSRWSASEIMGKLEG 385

Query: 1678 VAKKLNFRITGKKEFAVRMQGSTEGRKGKLAMTXXXXXXXXXXXXXXXTKSAGDTLEYIK 1857

+ K++N ++ K+F V++ G TEGRKG++A+T KSAGDTLEY +

Sbjct: 386 IGKEMNMKVKRTKDFKVKLFGKTEGRKGQIAVTAEVFEVAPEVAVVELCKSAGDTLEYNR 445

Query: 1858 FCEDQVRPSLKDIVWAWQGDN 1920

E+ VRP+L++IVW+W GDN

Sbjct: 446 LYEEHVRPALEEIVWSWHGDN 466

OLIGO [start](http://bioinfo.ut.ee/primer3-0.4.0/primer3_www_results_help.html#PRIMER_START)  [len](http://bioinfo.ut.ee/primer3-0.4.0/primer3_www_results_help.html#PRIMER_LEN)  [tm](http://bioinfo.ut.ee/primer3-0.4.0/primer3_www_results_help.html#PRIMER_TM)  [gc%](http://bioinfo.ut.ee/primer3-0.4.0/primer3_www_results_help.html#PRIMER_GC)  [any](http://bioinfo.ut.ee/primer3-0.4.0/primer3_www_results_help.html#PRIMER_ANY)  [3'](http://bioinfo.ut.ee/primer3-0.4.0/primer3_www_results_help.html#PRIMER_REPEAT) [seq](http://bioinfo.ut.ee/primer3-0.4.0/primer3_www_results_help.html#PRIMER_OLIGO_SEQ)

LEFT PRIMER 65 20 59.98 55.00 3.00 0.00 CAAGGGAGCACTGAAGGAAG

RIGHT PRIMER 237 20 59.42 45.00 5.00 3.00 CATGCCCACACAATGTCTTT

SEQUENCE SIZE: 241

INCLUDED REGION SIZE: 241

PRODUCT SIZE: 173, PAIR ANY COMPL: 3.00, PAIR 3' COMPL: 2.00

1 GGAAGGGGTGGCTAAGAAGCTTAATTTTAGGATAACGGGGAAGAAGGAGTTCGCGGTGAG

61 GATGCAAGGGAGCACTGAAGGAAGGAAGGGGAAGCTGGCAATGACGGTGGAGGTGTTTGA

>>>>>>>>>>>>>>>>>>>>

121 GGTGGCGCCGGAGGTGGCGGTGGTGGAGTTTACTAAGTCTGCCGGTGACACGTTGGAGTA

181 TATTAAATTCTGTGAGGACCAAGTGAGACCATCACTCAAAGACATTGTGTGGGCATGGCA

<<<<<<<<<<<<<<<<<<<<

241 G

KEYS (in order of precedence):

>>>>>> left primer

<<<<<< right primer

ADDITIONAL OLIGOS

[start](http://bioinfo.ut.ee/primer3-0.4.0/primer3_www_results_help.html#PRIMER_START)  [len](http://bioinfo.ut.ee/primer3-0.4.0/primer3_www_results_help.html#PRIMER_LEN)  [tm](http://bioinfo.ut.ee/primer3-0.4.0/primer3_www_results_help.html#PRIMER_TM)  [gc%](http://bioinfo.ut.ee/primer3-0.4.0/primer3_www_results_help.html#PRIMER_GC)  [any](http://bioinfo.ut.ee/primer3-0.4.0/primer3_www_results_help.html#PRIMER_ANY)  [3'](http://bioinfo.ut.ee/primer3-0.4.0/primer3_www_results_help.html#PRIMER_REPEAT) [seq](http://bioinfo.ut.ee/primer3-0.4.0/primer3_www_results_help.html#PRIMER_OLIGO_SEQ)

1 LEFT PRIMER 30 20 59.90 55.00 2.00 0.00 GGATAACGGGGAAGAAGGAG

RIGHT PRIMER 237 20 59.42 45.00 5.00 3.00 CATGCCCACACAATGTCTTT

PRODUCT SIZE: 208, PAIR ANY COMPL: 4.00, PAIR 3' COMPL: 2.00

2 LEFT PRIMER 29 20 59.90 50.00 2.00 0.00 AGGATAACGGGGAAGAAGGA

RIGHT PRIMER 237 20 59.42 45.00 5.00 3.00 CATGCCCACACAATGTCTTT

PRODUCT SIZE: 209, PAIR ANY COMPL: 4.00, PAIR 3' COMPL: 3.00

3 LEFT PRIMER 75 20 60.18 55.00 2.00 0.00 CTGAAGGAAGGAAGGGGAAG

RIGHT PRIMER 237 20 59.42 45.00 5.00 3.00 CATGCCCACACAATGTCTTT

PRODUCT SIZE: 163, PAIR ANY COMPL: 3.00, PAIR 3' COMPL: 2.00

4 LEFT PRIMER 65 20 59.98 55.00 3.00 0.00 CAAGGGAGCACTGAAGGAAG

RIGHT PRIMER 213 21 60.10 52.38 3.00 1.00 GATGGTCTCACTTGGTCCTCA

PRODUCT SIZE: 149, PAIR ANY COMPL: 6.00, PAIR 3' COMPL: 3.00

**13** Unigene36201_All lipid transfer protein 3

| Unigene36201_All | 465 | 142 | 440 | 65.34562 | 203.9332 | 1.641935 | Up |
| --- | --- | --- | --- | --- | --- | --- | --- |

24

| Unigene36201_All | 465 | 142 | 190 | 65.34562 | 95.02309 | 0.540188 | Up |
| --- | --- | --- | --- | --- | --- | --- | --- |

1

| Unigene36201_All | 465 | 142 | 375 | 65.34562 | 187.3034 | 1.519215 | Up |
| --- | --- | --- | --- | --- | --- | --- | --- |

>Unigene36201_All size 465 gap 0 0%

GGCACAGTTGGTGGAGGTGCTGATTTTGTAAGGGATGTTGAGATTGCATT

GGGCAGGGAGTGACTCTGCGAACTTGTTATTGATACCAGGAATGGAACCA

GCAGCAGCTTTCAAGCAGTTGCACACGGCCTGGCGGTCAGCAGTGGTGGT

GGCAGAACCCACGACGGTCTTCACTCCGGCGCAACACCCTTGTGAAGCAG

TTCCACCCTGTGTCAGGTAAGGTAGGCATGGTGCTACACTCTGTGCTATC

TCACCACATGTAATGGCCTGGGCCATTCTAATAGCCATGCACATGATCAG

AACCACGCATGCCAACTTAATCAGGCTAGCCATTATATATAATTCTTAAT

TCTTAATGGAACAAATTCAGAAAGAAAAATGCTAGCTGCTATGTAATGTA

ATGTAATGAATGTATATATATATATATATTGCTTTGAAATTGGGACTGCA

CTTGGTGAGTTGAAG

CTTCAACTCACCAAGTGCAGTCCCAATTTCAAAGCAATATATATATATATATACATTCATTACATTACATTACATAGCAGCTAGCATTTTTCTTTCTGAATTTGTTCCATTAAGAATTAAGAATTATATATAATGGCTAGCCTGATTAAGTTGGCATGCGTGGTTCTGATCATGTGCATGGCTATTAGAATGGCCCAGGCCATTACATGTGGTGAGATAGCACAGAGTGTAGCACCATGCCTACCTTACCTGACACAGGGTGGAACTGCTTCACAAGGGTGTTGCGCCGGAGTGAAGACCGTCGTGGGTTCTGCCACCACCACTGCTGACCGCCAGGCCGTGTGCAACTGCTTGAAAGCTGCTGCTGGTTCCATTCCTGGTATCAATAACAAGTTCGCAGAGTCACTCCCTGCCCAATGCAATCTCAACATCCCTTACAAAATCAGCACCTCCACCAACTGTGCC

>**[AT5G59320](http://www.arabidopsis.org/servlets/TairObject?type=locus&name=AT5G59320).1** | Symbols: LTP3 | lipid transfer protein 3 |

chr5:23929051-23929492 FORWARD LENGTH=115

Length = 115

Plus Strand HSPs:

Score = 282 (104.3 bits), Expect = 5.2e-25, P = 5.2e-25

Identities = 52/111 (46%), Positives = 71/111 (63%), Frame = +1

Query: 133 MASLIKL-ACVVLIMCMAIRMAQAITCGEIAQSVAPCLPYLTQGGTASQGCCAGVKTVVG 309

MA ++ C+VL +C+ + AI+CG +A S+APC YL++GG CCAGVKT+

Sbjct: 1 MAFALRFFTCLVLTVCIVASVDAAISCGTVAGSLAPCATYLSKGGLVPPSCCAGVKTLNS 60

Query: 310 SATTTADRQAVCNCLKAAAGSIPGINNKFAESLPAQCNLNIPYKISTSTNC 462

A TT DRQ C C+++ A SI G+N A LP +C ++IPY IS STNC

Sbjct: 61 MAKTTPDRQQACRCIQSTAKSISGLNPSLASGLPGKCGVSIPYPISMSTNC 111

OLIGO [start](http://bioinfo.ut.ee/primer3-0.4.0/primer3_www_results_help.html#PRIMER_START)  [len](http://bioinfo.ut.ee/primer3-0.4.0/primer3_www_results_help.html#PRIMER_LEN)  [tm](http://bioinfo.ut.ee/primer3-0.4.0/primer3_www_results_help.html#PRIMER_TM)  [gc%](http://bioinfo.ut.ee/primer3-0.4.0/primer3_www_results_help.html#PRIMER_GC)  [any](http://bioinfo.ut.ee/primer3-0.4.0/primer3_www_results_help.html#PRIMER_ANY)  [3'](http://bioinfo.ut.ee/primer3-0.4.0/primer3_www_results_help.html#PRIMER_REPEAT) [seq](http://bioinfo.ut.ee/primer3-0.4.0/primer3_www_results_help.html#PRIMER_OLIGO_SEQ)

LEFT PRIMER 36 20 60.16 55.00 3.00 1.00 GACACAGGGTGGAACTGCTT

RIGHT PRIMER 240 20 59.97 50.00 2.00 0.00 GGTGGAGGTGCTGATTTTGT

SEQUENCE SIZE: 248

INCLUDED REGION SIZE: 248

PRODUCT SIZE: 205, PAIR ANY COMPL: 3.00, PAIR 3' COMPL: 0.00

1 ATAGCACAGAGTGTAGCACCATGCCTACCTTACCTGACACAGGGTGGAACTGCTTCACAA

>>>>>>>>>>>>>>>>>>>>

61 GGGTGTTGCGCCGGAGTGAAGACCGTCGTGGGTTCTGCCACCACCACTGCTGACCGCCAG

121 GCCGTGTGCAACTGCTTGAAAGCTGCTGCTGGTTCCATTCCTGGTATCAATAACAAGTTC

181 GCAGAGTCACTCCCTGCCCAATGCAATCTCAACATCCCTTACAAAATCAGCACCTCCACC

<<<<<<<<<<<<<<<<<<<<

241 AACTGTGC

KEYS (in order of precedence):

>>>>>> left primer

<<<<<< right primer

ADDITIONAL OLIGOS

[start](http://bioinfo.ut.ee/primer3-0.4.0/primer3_www_results_help.html#PRIMER_START)  [len](http://bioinfo.ut.ee/primer3-0.4.0/primer3_www_results_help.html#PRIMER_LEN)  [tm](http://bioinfo.ut.ee/primer3-0.4.0/primer3_www_results_help.html#PRIMER_TM)  [gc%](http://bioinfo.ut.ee/primer3-0.4.0/primer3_www_results_help.html#PRIMER_GC)  [any](http://bioinfo.ut.ee/primer3-0.4.0/primer3_www_results_help.html#PRIMER_ANY)  [3'](http://bioinfo.ut.ee/primer3-0.4.0/primer3_www_results_help.html#PRIMER_REPEAT) [seq](http://bioinfo.ut.ee/primer3-0.4.0/primer3_www_results_help.html#PRIMER_OLIGO_SEQ)

1 LEFT PRIMER 36 20 60.16 55.00 3.00 1.00 GACACAGGGTGGAACTGCTT

RIGHT PRIMER 243 20 59.97 50.00 2.00 0.00 GTTGGTGGAGGTGCTGATTT

PRODUCT SIZE: 208, PAIR ANY COMPL: 3.00, PAIR 3' COMPL: 0.00

2 LEFT PRIMER 37 20 60.16 55.00 3.00 3.00 ACACAGGGTGGAACTGCTTC

RIGHT PRIMER 240 20 59.97 50.00 2.00 0.00 GGTGGAGGTGCTGATTTTGT

PRODUCT SIZE: 204, PAIR ANY COMPL: 3.00, PAIR 3' COMPL: 0.00

3 LEFT PRIMER 37 20 60.16 55.00 3.00 3.00 ACACAGGGTGGAACTGCTTC

RIGHT PRIMER 243 20 59.97 50.00 2.00 0.00 GTTGGTGGAGGTGCTGATTT

PRODUCT SIZE: 207, PAIR ANY COMPL: 3.00, PAIR 3' COMPL: 0.00

4 LEFT PRIMER 32 20 59.45 55.00 5.00 1.00 ACCTGACACAGGGTGGAACT

RIGHT PRIMER 240 20 59.97 50.00 2.00 0.00 GGTGGAGGTGCTGATTTTGT

PRODUCT SIZE: 209, PAIR ANY COMPL: 4.00, PAIR 3' COMPL: 1.00

**14 CL1914.Contig1_All**

**cryptochrome-interacting basic-helix-loop-helix 1**

72

| CL1914.Contig1_All | 1773 | 146 | 441 | 17.62078 | 53.60659 | 1.605132 | Up |
| --- | --- | --- | --- | --- | --- | --- | --- |

24

| CL1914.Contig1_All | 1773 | 146 | 405 | 17.62078 | 53.12205 | 1.592033 | Up |
| --- | --- | --- | --- | --- | --- | --- | --- |

1

| CL1914.Contig1_All | 1773 | 146 | 344 | 17.62078 | 45.06268 | 1.354655 | Up |
| --- | --- | --- | --- | --- | --- | --- | --- |

>CL1914.Contig1_All size 1773 gap 0 0%

AAGTGGCTGTATGAGTGAATGATTCATAGATCCATTTTAAAAAGAAATCT

TCATTTTGATCAATAAGGTCTCAGCATTCCTATGCACTCAATATCTGTGG

GGCTCACATGTCTCTATCATCTATGAAAATCCACTCTGCTTTAAATCTAC

TTTCTACTTTAAGGATATCAGACCAAGAAAAAAAAAAAGGTGATTTAGAC

AAGTATGAATTTGTAGCTACCTGTAAATGGCTGAGAATGAAAGGATGCTG

TTAGCGCTTGATCAAAAGTCACACTGTAAAGGTTTTGGAAATCACCTTCC

CAAGTTGAGGAAGGTAAAATTTGAGTGAAACAGGATGATGAGTCAAGAAA

TGTTTCAGGCAAAGATACAGGTGCACTGGTGGTCCTTCGAAGCCCCATAT

CTGAAGGGTTTATCCCTATGTTGTTTATTAATCCACCACAACACGAAACA

AGTTGTGCTGAATTGAACTGAAGATGATAGGCAGGGTTAGTAGTCATGTC

TGATGACATCCCTATGGTTGGAAAACTTGAAGCACAAGCAGGAAACACCT

CTTTGGCAAACAGATCATCAATGTTGAAGTCAAGCCTTGGGTTTACAGCA

GCTAATTTCATAGACAAGAACTCAACTTGTCGTTGAAGAGACTGAACATA

GTTAATGATTTCATCAAGCATTCCAGCTTTTCCTGTAATTTTGTTGCAAC

CCGGTACTAAATCTTGCAAATACTTCATTCTCTCACTAATTTTTTCCCTT

CTAACTCTTTCAGCTAAGCTATGACTATCAGTGGCTTGACCACGACGTGC

TCGGACATGAATGTAGTCAGACTTTGGATTTTGAACCTCAGACCCTTTGG

AATTGTCCTTGGAACTATCACCACAAGTTTCCTTGTTGTTCCTATAATTT

GTGTTTGCCTTTGAATTCTTGTTACCGGTTTGCTCCGTGATCTTGGATTC

CCCTTCATCACCACCACTTACTTTGATCTTCTTGTCTTTGTTATCACTTT

CTGTTGCAACCTTGGAATTCTGAGCTTTCCTCTTCTTGAAGCTTTCTTTC

TCACATGTCTGCCTCATCTTCTCCGCTGCTGAAACCACGTCTCTGCCAGC

TGGACTGCTACATGTCCTTGAAATCGAGGAATTCACATCAAACCCAGAAG

CAGGTGGCAGTGTAGAAGACCCGAATCCCATGTCAAACTTCCCCAATTCG

GGCCACCCATTTTTCAAACTCGGGTCAGGCTTCATTGAGTGAGCTACCAC

CTCACCAAGCGTTGAATCATCACCACCCATCATCATCACCAACCCCAGAG

GATCTTGAACTTGAAGAGAAGAAGAGAACACGCCGTTAAACCCAGTTCCA

GAAGAGTACCCTTGGTGCTCCTGGTTGTGGTGGTGGTGTTCTTGTTGCCA

CTTCCTCCTTCCTCGTTGTCTTTCCAACACTGTCATGTCCGAACAGCTTC

CTCCTATATTCCCCGAAGTGTTGAGACAGTACTCCAACATGTTGTTCTTC

TTCTTATTCTTATTGTTATTGTTAGGCAGTGATGCTCTGTTCTGAAAAAA

AGAGTAAGTACTAGTATTGGCTCTGTTTTGCTGCTCAAGTAAAGGCAAAT

TCTTTAGGCGGAAACAAAGTAGGCCCTGTAACCCTTCATTCCCCCAACTC

CAGTGAGATAAATGTACAATGTTGCTAAGGTGCGCTCGTGCATAGTAGAA

AGTAGAAACCGAGAGGAAGAAAGAGAGGAGAAACTATGAAATGCTGTTAA

GGATGGAGAAAAAGAGAAGTTGG

CCAACTTCTCTTTTTCTCCATCCTTAACAGCATTTCATAGTTTCTCCTCTCTTTCTTCCTCTCGGTTTCTACTTTCTACTATGCACGAGCGCACCTTAGCAACATTGTACATTTATCTCACTGGAGTTGGGGGAATGAAGGGTTACAGGGCCTACTTTGTTTCCGCCTAAAGAATTTGCCTTTACTTGAGCAGCAAAACAGAGCCAATACTAGTACTTACTCTTTTTTTCAGAACAGAGCATCACTGCCTAACAATAACAATAAGAATAAGAAGAAGAACAACATGTTGGAGTACTGTCTCAACACTTCGGGGAATATAGGAGGAAGCTGTTCGGACATGACAGTGTTGGAAAGACAACGAGGAAGGAGGAAGTGGCAACAAGAACACCACCACCACAACCAGGAGCACCAAGGGTACTCTTCTGGAACTGGGTTTAACGGCGTGTTCTCTTCTTCTCTTCAAGTTCAAGATCCTCTGGGGTTGGTGATGATGATGGGTGGTGATGATTCAACGCTTGGTGAGGTGGTAGCTCACTCAATGAAGCCTGACCCGAGTTTGAAAAATGGGTGGCCCGAATTGGGGAAGTTTGACATGGGATTCGGGTCTTCTACACTGCCACCTGCTTCTGGGTTTGATGTGAATTCCTCGATTTCAAGGACATGTAGCAGTCCAGCTGGCAGAGACGTGGTTTCAGCAGCGGAGAAGATGAGGCAGACATGTGAGAAAGAAAGCTTCAAGAAGAGGAAAGCTCAGAATTCCAAGGTTGCAACAGAAAGTGATAACAAAGACAAGAAGATCAAAGTAAGTGGTGGTGATGAAGGGGAATCCAAGATCACGGAGCAAACCGGTAACAAGAATTCAAAGGCAAACACAAATTATAGGAACAACAAGGAAACTTGTGGTGATAGTTCCAAGGACAATTCCAAAGGGTCTGAGGTTCAAAATCCAAAGTCTGACTACATTCATGTCCGAGCACGTCGTGGTCAAGCCACTGATAGTCATAGCTTAGCTGAAAGAGTTAGAAGGGAAAAAATTAGTGAGAGAATGAAGTATTTGCAAGATTTAGTACCGGGTTGCAACAAAATTACAGGAAAAGCTGGAATGCTTGATGAAATCATTAACTATGTTCAGTCTCTTCAACGACAAGTTGAGTTCTTGTCTATGAAATTAGCTGCTGTAAACCCAAGGCTTGACTTCAACATTGATGATCTGTTTGCCAAAGAGGTGTTTCCTGCTTGTGCTTCAAGTTTTCCAACCATAGGGATGTCATCAGACATGACTACTAACCCTGCCTATCATCTTCAGTTCAATTCAGCACAACTTGTTTCGTGTTGTGGTGGATTAATAAACAACATAGGGATAAACCCTTCAGATATGGGGCTTCGAAGGACCACCAGTGCACCTGTATCTTTGCCTGAAACATTTCTTGACTCATCATCCTGTTTCACTCAAATTTTACCTTCCTCAACTTGGGAAGGTGATTTCCAAAACCTTTACAGTGTGACTTTTGATCAAGCGCTAACAGCATCCTTTCATTCTCAGCCATTTACAGGTAGCTACAAATTCATACTTGTCTAAATCACCTTTTTTTTTTTCTTGGTCTGATATCCTTAAAGTAGAAAGTAGATTTAAAGCAGAGTGGATTTTCATAGATGATAGAGACATGTGAGCCCCACAGATATTGAGTGCATAGGAATGCTGAGACCTTATTGATCAAAATGAAGATTTCTTTTTAAAATGGATCTATGAATCATTCACTCATACAGCCACTT

>**[AT4G34530](http://www.arabidopsis.org/servlets/TairObject?type=locus&name=AT4G34530).1** | Symbols: CIB1 | cryptochrome-interacting basic-helix-loop-helix

1 | chr4:16498466-16499946 FORWARD LENGTH=335

Length = 335

Plus Strand HSPs:

Score = 499 (180.7 bits), Expect = 2.7e-57, Sum P(3) = 2.7e-57

Identities = 121/232 (52%), Positives = 156/232 (67%), Frame = +2

Query: 731 SFKKRKAQN-SKVATESDNK---DKKIKVSGGDEGESKITEQT-GNKNSKANTNYRNNKE 895

+FKKRK +K E K ++ V G+E +SKITEQ G+ S ++ KE

Sbjct: 90 NFKKRKFDTETKDCNEKKKKMTMNRDDLVEEGEEEKSKITEQNNGSTKSIKKMKHKAKKE 149

Query: 896 TCGDSSKDNSKGSEVQNPKSDYIHVRARRGQATDSHSLAERVRREKISERMKYLQDLVPG 1075

+ S D+SK ++ + K+DYIHVRARRGQATDSHS+AERVRREKISERMK+LQDLVPG

Sbjct: 150 E-NNFSNDSSKVTK-ELEKTDYIHVRARRGQATDSHSIAERVRREKISERMKFLQDLVPG 207

Query: 1076 CNKITGKAGMLDEIINYVQSLQRQVEFLSMKLAAVNPRLDFNIDDLFAKEVFPACASSFP 1255

C+KITGKAGMLDEIINYVQSLQRQ+EFLSMKLA VNPR DF++DD+FAKEV AS+

Sbjct: 208 CDKITGKAGMLDEIINYVQSLQRQIEFLSMKLAIVNPRPDFDMDDIFAKEV----ASTPM 263

Query: 1256 TIGMSSDMTTNPAYHLQFNSAQLVSCCGGLINNIGINPSDMGLRRTTSAPVS 1411

T+ S +M + H +S ++N+ ++ + M T+S P+S

Sbjct: 264 TVVPSPEMVLSGYSHEMVHSGY----SSEMVNSGYLHVNPMQQVNTSSDPLS 311

Score = 63 (27.2 bits), Expect = 2.7e-57, Sum P(3) = 2.7e-57

Identities = 30/99 (30%), Positives = 40/99 (40%), Frame = +2

Query: 308 SGNIGGSC----SDMTVLERQRGRRKWXXXXXXXXXXXXGYSSGTGFNGVFSSSLQVQDP 475

+G IGG DM+VLERQR K+ S G S L

Sbjct: 2 NGAIGGDLLLNFPDMSVLERQRAHLKYLNPTFDSPLAGFFADSSMITGGEMDSYLSTAG- 60

Query: 476 LGLVMMMGGDDSTLGEVVAHSMKPDPSLKNGWPELGKFD 592

L L MM G ++T+ S+ P+ +L G + KFD

Sbjct: 61 LNLPMMYG--ETTVEGDSRLSISPETTLGTGNFKKRKFD 97

Score = 54 (24.1 bits), Expect = 2.7e-57, Sum P(3) = 2.7e-57

Identities = 12/32 (37%), Positives = 14/32 (43%), Frame = +2

Query: 1403 PVSLPETFLDSSSCFTQILPSSTWEGDFQNLY 1498

P+ T D SCF S W+ QNLY

Sbjct: 299 PMQQVNTSSDPLSCFNNGEAPSMWDSHVQNLY 330

OLIGO [start](http://bioinfo.ut.ee/primer3-0.4.0/primer3_www_results_help.html#PRIMER_START)  [len](http://bioinfo.ut.ee/primer3-0.4.0/primer3_www_results_help.html#PRIMER_LEN)  [tm](http://bioinfo.ut.ee/primer3-0.4.0/primer3_www_results_help.html#PRIMER_TM)  [gc%](http://bioinfo.ut.ee/primer3-0.4.0/primer3_www_results_help.html#PRIMER_GC)  [any](http://bioinfo.ut.ee/primer3-0.4.0/primer3_www_results_help.html#PRIMER_ANY)  [3'](http://bioinfo.ut.ee/primer3-0.4.0/primer3_www_results_help.html#PRIMER_REPEAT) [seq](http://bioinfo.ut.ee/primer3-0.4.0/primer3_www_results_help.html#PRIMER_OLIGO_SEQ)

LEFT PRIMER 75 20 60.09 45.00 7.00 0.00 TTTGCCAAAGAGGTGTTTCC

RIGHT PRIMER 202 20 60.04 50.00 2.00 0.00 CCACCACAACACGAAACAAG

SEQUENCE SIZE: 265

INCLUDED REGION SIZE: 265

PRODUCT SIZE: 128, PAIR ANY COMPL: 6.00, PAIR 3' COMPL: 3.00

1 AACGACAAGTTGAGTTCTTGTCTATGAAATTAGCTGCTGTAAACCCAAGGCTTGACTTCA

61 ACATTGATGATCTGTTTGCCAAAGAGGTGTTTCCTGCTTGTGCTTCAAGTTTTCCAACCA

>>>>>>>>>>>>>>>>>>>>

121 TAGGGATGTCATCAGACATGACTACTAACCCTGCCTATCATCTTCAGTTCAATTCAGCAC

181 AACTTGTTTCGTGTTGTGGTGGATTAATAAACAACATAGGGATAAACCCTTCAGATATGG

<<<<<<<<<<<<<<<<<<<<

241 GGCTTCGAAGGACCACCAGTGCACC

KEYS (in order of precedence):

>>>>>> left primer

<<<<<< right primer

ADDITIONAL OLIGOS

[start](http://bioinfo.ut.ee/primer3-0.4.0/primer3_www_results_help.html#PRIMER_START)  [len](http://bioinfo.ut.ee/primer3-0.4.0/primer3_www_results_help.html#PRIMER_LEN)  [tm](http://bioinfo.ut.ee/primer3-0.4.0/primer3_www_results_help.html#PRIMER_TM)  [gc%](http://bioinfo.ut.ee/primer3-0.4.0/primer3_www_results_help.html#PRIMER_GC)  [any](http://bioinfo.ut.ee/primer3-0.4.0/primer3_www_results_help.html#PRIMER_ANY)  [3'](http://bioinfo.ut.ee/primer3-0.4.0/primer3_www_results_help.html#PRIMER_REPEAT) [seq](http://bioinfo.ut.ee/primer3-0.4.0/primer3_www_results_help.html#PRIMER_OLIGO_SEQ)

1 LEFT PRIMER 85 20 59.91 50.00 3.00 0.00 AGGTGTTTCCTGCTTGTGCT

RIGHT PRIMER 243 20 60.15 50.00 4.00 0.00 GCCCCATATCTGAAGGGTTT

PRODUCT SIZE: 159, PAIR ANY COMPL: 3.00, PAIR 3' COMPL: 0.00

2 LEFT PRIMER 75 20 60.09 45.00 7.00 0.00 TTTGCCAAAGAGGTGTTTCC

RIGHT PRIMER 243 20 60.15 50.00 4.00 0.00 GCCCCATATCTGAAGGGTTT

PRODUCT SIZE: 169, PAIR ANY COMPL: 3.00, PAIR 3' COMPL: 0.00

3 LEFT PRIMER 85 20 59.91 50.00 3.00 0.00 AGGTGTTTCCTGCTTGTGCT

RIGHT PRIMER 248 20 60.17 50.00 4.00 2.00 TCGAAGCCCCATATCTGAAG

PRODUCT SIZE: 164, PAIR ANY COMPL: 4.00, PAIR 3' COMPL: 2.00

4 LEFT PRIMER 85 20 59.91 50.00 3.00 0.00 AGGTGTTTCCTGCTTGTGCT

RIGHT PRIMER 250 20 60.17 50.00 8.00 3.00 CTTCGAAGCCCCATATCTGA

PRODUCT SIZE: 166, PAIR ANY COMPL: 4.00, PAIR 3' COMPL: 1.00

**15 Unigene32219_All** HXXXD-type acyl-transferase family protein, shikimate O-hydroxycinnamoyltransferase

**72**

| Unigene32219_All | 1666 | 1365 | 387 | 175.3229 | 50.06385 | -1.80817 | Down |
| --- | --- | --- | --- | --- | --- | --- | --- |

**24**

| Unigene32219_All | 1666 | 1365 | 339 | 175.3229 | 47.32092 | -1.88946 | Down |
| --- | --- | --- | --- | --- | --- | --- | --- |

**1**

| Unigene32219_All | 1666 | 1365 | 1880 | 175.3229 | 262.0898 | 0.580047 | Up |
| --- | --- | --- | --- | --- | --- | --- | --- |

**>Unigene32219_All size 1666 gap 0 0%**

GGAAACACACCCCACTTTCTCACCTCCACACCTATAAAATGCCCTCTCCCCCAATTCTCTTGGGAACCAAGCCTTCTTCTCGACACATTCCCTTACCAACACCATAATATTAACCAATAATAACAAAATGCCTTCTGCTTCCACAACCTTCATCTCCAAATGTAAAATCTACCCAGACCAAAAATCCACTATGAAACCCCTCAAGCTTTCTGTCTCTGACCTTCCAATGCTTTCTTGTCACTACATCCAAAAGGGTGTCCTCTTAACTTCCCCACCTTACTCCTTTGATGACCTTATCATCTCACTCAAGCATTCCCTCTCCGCCGCCCTCTCTCACTTCCCCGCCCTTGCCGGCCGCTTCTCCACCGACGACGAAGGCTACGTTTACATCCTCTGTAACGACGCCGGCGCCGATTTCATCCACGCCAAGGCCAAGCACCTCACAATCAACGCCATCCTCAATCCAACGCTTACCGATGTTCCCAGTTGCTTCAAGGAGTTCTTCGCCTACGACATGACGATTTCCTACTCCGGCCACCACAACCCCCTCGCCGCCGTGAAGGTCACCGAGCTCGCCGACGGAGTATTCATCGGAATCACTATTAACCACGCCGTCACCGACGGGACATCTTTCTGGCACTTCTTCAACACGTTCGCCGCCGTATGTAAGGCCGGAACCGGCGTCAAGAAGATTTTTCTCAGGTCACCGGACTTCACGCGGGATACTGTGTTCAACTCCGCTGCCGTCCTCGCCATACCCGGAGGCGGACCCACCGTCACCTTCGCCGCCGACGAGCCTCTCCGGGAGCGAGTCTTTCACTTCACCCGCGAAGCTATTCAGAAACTCAAACTGAAAGCTAACTGTAACGGCTCCGGCAATGGTAACGGTTTCCCAGATTCGGTTGAGGTGATGGGGAAACAAGTGAATGACAGTTGGAAAACCGTTAACGGTAACAGAAAGATGAGGGAGATCTCGTCCTTTCAATCATTATCTGCTCAGCTTTGGCGTTCGGTGACACGTGCCAGGAAATTAGTGCCATCGAAAACGTCGACGTTTCGCATGGCGGTTAACTGTCGCCACAGGTTGGAACCGAAGATGGACCCGTTTTACTTCGGTAACGCGATTCAGAGCATCCCGACCGTTGCTTCTGTAGGTGACATCCTATCACGTGATCTTCGCTTTTGCGCTGAGCTGCTTCATCGAAATGTCGTCGCACATGACGATGCTACGGTGCGCCACGGCGTAGAGGATTGGGAAAAGGCTCCTAGGTTGTTCCCTCTTGGGAATTTCGACGGTGCTATGATCACTATGGGTAGCTCTCCGCGTTTTCCCATGTACGACAATGACTTCGGGTGGGGCCGACCCTTGGCTGTTAGGAGCGGAAAGGCGAATAAGTTTGATGGTAAGATCTCTGCGTTTCCGGGTAGAGAAGGAAACGGCAGCGTTGATCTTGAAGTTGTTTTGGCACCCGAAACGATGGCTGGGCTCGAAAACGACATGGAGTTTATGCAATATGTAACATCATTCGTGTGTGTGGTTTAAGATTATACAGTAGTAAAAGCTCACGACGTACGTCTCTCGATGATTAAACGACGAAAATGGATGGTCATGCAACAATAGTACGATGGGTTTGGGCGTCGTTTTGGTTTGGTCAATGTTCGCGGG

>**[AT5G01210](http://www.arabidopsis.org/servlets/TairObject?type=locus&name=AT5G01210).1** | Symbols: | HXXXD-type acyl-transferase family protein |

chr5:84554-85981 FORWARD LENGTH=475

Length = 475

Plus Strand HSPs:

Score = 1613 (572.9 bits), Expect = 4.3e-166, P = 4.3e-166

Identities = 320/476 (67%), Positives = 369/476 (77%), Frame = +2

Query: 128 MPSASTTFISKCKIYPDQKSTMKPLKLSVSDLPMLSCHYIQKGVLLTSPP--YSFDDXXX 301

MPS S T ISKC +YP++KST+ L+LSVSDLPMLSCHYIQKGVLLTSPP +SFDD

Sbjct: 1 MPSCSVTEISKCIVYPEKKSTVSDLRLSVSDLPMLSCHYIQKGVLLTSPPPSFSFDDLVS 60

Query: 302 XXXXXXXXXXXXFPALAGRFSTDDEGYVYILCNDAGADFIHAKAKHLTINAILNPTLTDV 481

FPALAGRFST G++ I+CNDAG DF+ A AKH+ ++ +L P DV

Sbjct: 61 SLRRSLSSTLSLFPALAGRFSTTPAGHISIVCNDAGVDFVAASAKHVKLSDVLLPG-EDV 119

Query: 482 PSCFKEFFAYDMTISYSGHHNPLAAVKVTELADGVFIGITINHAVTDGTSFWHFFNTFAA 661

P F+EFF ++ +SY+GHH PLAAV+VTEL DGVFIG T+NH+VTDGTSFWHFFNTFA

Sbjct: 120 PLLFREFFVFERLVSYNGHHKPLAAVQVTELHDGVFIGCTVNHSVTDGTSFWHFFNTFAD 179

Query: 662 VCKAGTGVKKIFLRSPDFTRDTVFNSAAVLAIPGGGPTVTFAADEPLRERVFHFTREAIQ 841

V +K + PDF+R TVF+S VL +P GGP VTF AD+PLRER+FHF+REAI

Sbjct: 180 VTSGACKIKHL----PDFSRHTVFDSPVVLPVPPGGPRVTFDADQPLRERIFHFSREAIT 235

Query: 842 KLKLKAN--CNG--SGNGNGFPDSVEVMGK--QVNDSWKTVNGNR---KMREISSFQSLS 994

KLK + N NG + +G + E+ GK V DS+ +N + + EISSFQSLS

Sbjct: 236 KLKQRTNNRVNGIETAVNDGRKCNGEINGKITTVLDSF--LNNKKSYDRTAEISSFQSLS 293

Query: 995 AQLWRSVTRARKLVPSKTSTFRMAVNCRHRLEPKMDPFYFGNAIQSIPTVASVGDILSRD 1174

AQLWRSVTRAR L PSKT+TFRMAVNCRHRLEPKMDP+YFGNAIQSIPT+AS GD+LS+D

Sbjct: 294 AQLWRSVTRARNLDPSKTTTFRMAVNCRHRLEPKMDPYYFGNAIQSIPTLASAGDLLSKD 353

Query: 1175 LRFCAELLHRNVVAHDDATVRHGVEDWEKAPRLFPLGNFDGAMITMGSSPRFPMYDNDFG 1354

LR+ AE LHRNVVAHDDATVR G+ WE PRLFPLGN DGA ITMGSSPRFPMYDNDFG

Sbjct: 354 LRWSAEQLHRNVVAHDDATVRRGIAAWESDPRLFPLGNPDGASITMGSSPRFPMYDNDFG 413

Query: 1355 WGRPLAVRSGKANKFDGKISAFPGREGNGSVDLEVVLAPETMAGLENDMEFMQYVT 1522

WG+PLAVRSG ANKFDGKISAFPGREGNGSVDLEVVLAPETM G+END EFMQYV+

Sbjct: 414 WGKPLAVRSGGANKFDGKISAFPGREGNGSVDLEVVLAPETMTGIENDAEFMQYVS 469

OLIGO [start](http://bioinfo.ut.ee/primer3-0.4.0/primer3_www_results_help.html#PRIMER_START)  [len](http://bioinfo.ut.ee/primer3-0.4.0/primer3_www_results_help.html#PRIMER_LEN)  [tm](http://bioinfo.ut.ee/primer3-0.4.0/primer3_www_results_help.html#PRIMER_TM)  [gc%](http://bioinfo.ut.ee/primer3-0.4.0/primer3_www_results_help.html#PRIMER_GC)  [any](http://bioinfo.ut.ee/primer3-0.4.0/primer3_www_results_help.html#PRIMER_ANY)  [3'](http://bioinfo.ut.ee/primer3-0.4.0/primer3_www_results_help.html#PRIMER_REPEAT) [seq](http://bioinfo.ut.ee/primer3-0.4.0/primer3_www_results_help.html#PRIMER_OLIGO_SEQ)

LEFT PRIMER 6 20 60.07 55.00 6.00 2.00 GGGAAAAGGCTCCTAGGTTG

RIGHT PRIMER 176 20 60.34 50.00 6.00 2.00 CCGGAAACGCAGAGATCTTA

SEQUENCE SIZE: 288

INCLUDED REGION SIZE: 288

PRODUCT SIZE: 171, PAIR ANY COMPL: 3.00, PAIR 3' COMPL: 1.00

1 GGATTGGGAAAAGGCTCCTAGGTTGTTCCCTCTTGGGAATTTCGACGGTGCTATGATCAC

>>>>>>>>>>>>>>>>>>>>

61 TATGGGTAGCTCTCCGCGTTTTCCCATGTACGACAATGACTTCGGGTGGGGCCGACCCTT

121 GGCTGTTAGGAGCGGAAAGGCGAATAAGTTTGATGGTAAGATCTCTGCGTTTCCGGGTAG

<<<<<<<<<<<<<<<<<<<<

181 AGAAGGAAACGGCAGCGTTGATCTTGAAGTTGTTTTGGCACCCGAAACGATGGCTGGGCT

241 CGAAAACGACATGGAGTTTATGCAATATGTAACATCATTCGTGTGTGT

KEYS (in order of precedence):

>>>>>> left primer

<<<<<< right primer

ADDITIONAL OLIGOS

[start](http://bioinfo.ut.ee/primer3-0.4.0/primer3_www_results_help.html#PRIMER_START)  [len](http://bioinfo.ut.ee/primer3-0.4.0/primer3_www_results_help.html#PRIMER_LEN)  [tm](http://bioinfo.ut.ee/primer3-0.4.0/primer3_www_results_help.html#PRIMER_TM)  [gc%](http://bioinfo.ut.ee/primer3-0.4.0/primer3_www_results_help.html#PRIMER_GC)  [any](http://bioinfo.ut.ee/primer3-0.4.0/primer3_www_results_help.html#PRIMER_ANY)  [3'](http://bioinfo.ut.ee/primer3-0.4.0/primer3_www_results_help.html#PRIMER_REPEAT) [seq](http://bioinfo.ut.ee/primer3-0.4.0/primer3_www_results_help.html#PRIMER_OLIGO_SEQ)

1 LEFT PRIMER 5 20 60.07 50.00 6.00 2.00 TGGGAAAAGGCTCCTAGGTT

RIGHT PRIMER 176 20 60.34 50.00 6.00 2.00 CCGGAAACGCAGAGATCTTA

PRODUCT SIZE: 172, PAIR ANY COMPL: 3.00, PAIR 3' COMPL: 2.00

2 LEFT PRIMER 1 20 60.39 50.00 4.00 2.00 GGATTGGGAAAAGGCTCCTA

RIGHT PRIMER 176 20 60.34 50.00 6.00 2.00 CCGGAAACGCAGAGATCTTA

PRODUCT SIZE: 176, PAIR ANY COMPL: 3.00, PAIR 3' COMPL: 2.00

3 LEFT PRIMER 36 20 60.47 50.00 5.00 0.00 GGAATTTCGACGGTGCTATG

RIGHT PRIMER 176 20 60.34 50.00 6.00 2.00 CCGGAAACGCAGAGATCTTA

PRODUCT SIZE: 141, PAIR ANY COMPL: 4.00, PAIR 3' COMPL: 2.00

4 LEFT PRIMER 5 20 60.07 50.00 6.00 2.00 TGGGAAAAGGCTCCTAGGTT

RIGHT PRIMER 223 20 60.90 45.00 3.00 3.00 GGGTGCCAAAACAACTTCAA

PRODUCT SIZE: 219, PAIR ANY COMPL: 4.00, PAIR 3' COMPL: 2.00

**16. Unigene3444_All**

**· and stilbene synthase表达模式为1h，升，然后24h，72h下降**

72

| Unigene3444_All | 1248 | 143 | 42 | 24.51899 | 7.253089 | -1.75723 | Down |
| --- | --- | --- | --- | --- | --- | --- | --- |

24

| Unigene3444_All | 1248 | 143 | 94 | 24.51899 | 17.51628 | -0.4852 | Down |
| --- | --- | --- | --- | --- | --- | --- | --- |

1

| Unigene3444_All | 1248 | 143 | 307 | 24.51899 | 57.13354 | 1.220438 | Up |
| --- | --- | --- | --- | --- | --- | --- | --- |

>Unigene3444_All size 1248 gap 0 0%

ACACATGCACTGGACATGTTACCATATTCACTAAGAACATGTCTGGTGGC

CCTCATCTTTTCTTCCTTCAAGCCCAACTTTGCCTCAACCTGGTCTAAAA

TTGCAGGCCCACCCGGGTGTGCAATCCAAAAGATAGAGTTGTAATCAGAT

ATGTTTAATGGGTTGAAGGCTTCAACCAATGCCTTGTCAATGTTCTTTGA

GACGATCCCTGGAACATCTTTAAGGAGGTGAAATGTTAGCCCAACTTCAC

GAAGGTGCCCATCAATTGCTCCTTCACTATCTGGAGCAATTGTTTGAGCA

GTCCAAACTAACTCAAATATAGGTTTCTCAATCTCTGGCACTGGATCAGA

ACCAACAATAAGTGCAGCTGCTCCATCTCCAAACAATGCTTGACCCACAA

GACTATCTAGGTGAGTGTCACTGGGGCCACGGAATGTAACTGCAGTAATC

TCAGAGCAAACAACTAGCACACGTGCACCTTTGTTATTCTCAGCCAAATC

TTTGGCCAAACGAAGCACGGTACCACCTGCAAAGCACCCTTGTTGGTACA

TCATGTACCTCTTCACATATGGGCGAAGACCTAAGAGTTTGGTGAGTTGG

TAATCAGCACCAGGCATGTCTACACCACTAGTGGTGCAAAAGACTAAGTG

AGTAATCTTGGACTTTGGCTGACCCCATTCTTTTATGGCCTTGGTTGCAG

CCTCTTTCCCTAGTCTTGGTACCTCTACCACCACCATGTCTTGCCTAGCA

TCCAAGGAAGGTGCCATATAAGCACAAATGTTGGGGTTCTCTTTCAAGAT

CTCTTCCGTTAAGTGCATATATCGCTTCCTGATCATGGACTTGTCACACA

TGCGCTGAAATTTCTCTTTAAGCTCAGTTTTGTGCTCGCTGTGTGTGATC

CTGAAGTAGTAATCGGGATAGGTGCTTTGTTCAACACAGTTTGGTGGAGT

TGCAGTGCCAATGGCCATGATGGTAGCAGGACCTTCTGCCCTTTGAGCCT

TGCGGATCTCTTCTACGCTCACCATCTTTCCTTTCTATAAAGCTTCTCTC

AAATCAAGCTCCTACGTACACAAATATATTGAGATTGTGAAAAGTAATAA

TGGAGCTATAGTTTTATTGCAGTACTTTAGTAGAATAGAAGAGGGGTGAT

GGGTGTGCTACAAACTTGTTAGGGAGGAGGGGATTTTATATGTATGTCAT

GTAATAATGGGATGGGAGAGTGGGGAAGGCATTATGGGAGTTTAGACT

AGTCTAAACTCCCATAATGCCTTCCCCACTCTCCCATCCCATTATTACATGACATACATATAAAATCCCCTCCTCCCTAACAAGTTTGTAGCACACCCATCACCCCTCTTCTATTCTACTAAAGTACTGCAATAAAACTATAGCTCCATTATTACTTTTCACAATCTCAATATATTTGTGTACGTAGGAGCTTGATTTGAGAGAAGCTTTATAGAAAGGAAAGATGGTGAGCGTAGAAGAGATCCGCAAGGCTCAAAGGGCAGAAGGTCCTGCTACCATCATGGCCATTGGCACTGCAACTCCACCAAACTGTGTTGAACAAAGCACCTATCCCGATTACTACTTCAGGATCACACACAGCGAGCACAAAACTGAGCTTAAAGAGAAATTTCAGCGCATGTGTGACAAGTCCATGATCAGGAAGCGATATATGCACTTAACGGAAGAGATCTTGAAAGAGAACCCCAACATTTGTGCTTATATGGCACCTTCCTTGGATGCTAGGCAAGACATGGTGGTGGTAGAGGTACCAAGACTAGGGAAAGAGGCTGCAACCAAGGCCATAAAAGAATGGGGTCAGCCAAAGTCCAAGATTACTCACTTAGTCTTTTGCACCACTAGTGGTGTAGACATGCCTGGTGCTGATTACCAACTCACCAAACTCTTAGGTCTTCGCCCATATGTGAAGAGGTACATGATGTACCAACAAGGGTGCTTTGCAGGTGGTACCGTGCTTCGTTTGGCCAAAGATTTGGCTGAGAATAACAAAGGTGCACGTGTGCTAGTTGTTTGCTCTGAGATTACTGCAGTTACATTCCGTGGCCCCAGTGACACTCACCTAGATAGTCTTGTGGGTCAAGCATTGTTTGGAGATGGAGCAGCTGCACTTATTGTTGGTTCTGATCCAGTGCCAGAGATTGAGAAACCTATATTTGAGTTAGTTTGGACTGCTCAAACAATTGCTCCAGATAGTGAAGGAGCAATTGATGGGCACCTTCGTGAAGTTGGGCTAACATTTCACCTCCTTAAAGATGTTCCAGGGATCGTCTCAAAGAACATTGACAAGGCATTGGTTGAAGCCTTCAACCCATTAAACATATCTGATTACAACTCTATCTTTTGGATTGCACACCCGGGTGGGCCTGCAATTTTAGACCAGGTTGAGGCAAAGTTGGGCTTGAAGGAAGAAAAGATGAGGGCCACCAGACATGTTCTTAGTGAATATGGTAACATGTCCAGTGCATGTGT

>**[AT5G13930](http://www.arabidopsis.org/servlets/TairObject?type=locus&name=AT5G13930).1** | Symbols: CHS, TT4, ATCHS | Chalcone and stilbene synthase family

protein | chr5:4488762-4490035 FORWARD LENGTH=395

Length = 395

Plus Strand HSPs:

Score = 1506 (535.2 bits), Expect = 1.0e-154, P = 1.0e-154

Identities = 289/340 (85%), Positives = 314/340 (92%), Frame = +2

Query: 230 SVEEIRKAQRAEGPATIMAIGTATPPNCVEQSTYPDYYFRITHSEHKTELKEKFQRMCDK 409

S++EIR+AQRA+GPA I+AIGTA P N V Q+ YPDYYFRIT+SEH T+LKEKF+RMCDK

Sbjct: 8 SLDEIRQAQRADGPAGILAIGTANPENHVLQAEYPDYYFRITNSEHMTDLKEKFKRMCDK 67

Query: 410 SMIRKRYMHLTEEILKENPNICAYMAPSLDARQDMVVVEVPRLGKEAATKAIKEWGQPKS 589

S IRKR+MHLTEE LKENP++CAYMAPSLD RQD+VVVEVP+LGKEAA KAIKEWGQPKS

Sbjct: 68 STIRKRHMHLTEEFLKENPHMCAYMAPSLDTRQDIVVVEVPKLGKEAAVKAIKEWGQPKS 127

Query: 590 KITHLVFCTTSGVDMPGADYQLTKLLGLRPYVKRYMMYQQGCFAGGTVLRLAKDLAENNK 769

KITH+VFCTTSGVDMPGADYQLTKLLGLRP VKR MMYQQGCFAGGTVLR+AKDLAENN+

Sbjct: 128 KITHVVFCTTSGVDMPGADYQLTKLLGLRPSVKRLMMYQQGCFAGGTVLRIAKDLAENNR 187

Query: 770 GARVLVVCSEITAVTFRGPSDTHLDSLVGQALFGDGAAALIVGSDPVPEI-EKPIFELVW 946

GARVLVVCSEITAVTFRGPSDTHLDSLVGQALF DGAAALIVGSDP + EKPIFE+V

Sbjct: 188 GARVLVVCSEITAVTFRGPSDTHLDSLVGQALFSDGAAALIVGSDPDTSVGEKPIFEMVS 247

Query: 947 TAQTIAPDSEGAIDGHLREVGLTFHLLKDVPGIVSKNIDKALVEAFNPLNISDYNSIFWI 1126

AQTI PDS+GAIDGHLREVGLTFHLLKDVPG++SKNI K+L EAF PL ISD+NS+FWI

Sbjct: 248 AAQTILPDSDGAIDGHLREVGLTFHLLKDVPGLISKNIVKSLDEAFKPLGISDWNSLFWI 307

Query: 1127 AHPGGPAILDQVEAKLGLKEEKMRATRHVLSEYGNMSSAC 1246

AHPGGPAILDQVE KLGLKEEKMRATRHVLSEYGNMSSAC

Sbjct: 308 AHPGGPAILDQVEIKLGLKEEKMRATRHVLSEYGNMSSAC 347

OLIGO [start](http://bioinfo.ut.ee/primer3-0.4.0/primer3_www_results_help.html#PRIMER_START)  [len](http://bioinfo.ut.ee/primer3-0.4.0/primer3_www_results_help.html#PRIMER_LEN)  [tm](http://bioinfo.ut.ee/primer3-0.4.0/primer3_www_results_help.html#PRIMER_TM)  [gc%](http://bioinfo.ut.ee/primer3-0.4.0/primer3_www_results_help.html#PRIMER_GC)  [any](http://bioinfo.ut.ee/primer3-0.4.0/primer3_www_results_help.html#PRIMER_ANY)  [3'](http://bioinfo.ut.ee/primer3-0.4.0/primer3_www_results_help.html#PRIMER_REPEAT) [seq](http://bioinfo.ut.ee/primer3-0.4.0/primer3_www_results_help.html#PRIMER_OLIGO_SEQ)

LEFT PRIMER 48 20 60.16 50.00 8.00 3.00 TGGAGCAGCTGCACTTATTG

RIGHT PRIMER 228 20 60.19 50.00 4.00 3.00 CTTTGAGACGATCCCTGGAA

SEQUENCE SIZE: 291

INCLUDED REGION SIZE: 291

PRODUCT SIZE: 181, PAIR ANY COMPL: 3.00, PAIR 3' COMPL: 1.00

1 AGTGACACTCACCTAGATAGTCTTGTGGGTCAAGCATTGTTTGGAGATGGAGCAGCTGCA

>>>>>>>>>>>>>

61 CTTATTGTTGGTTCTGATCCAGTGCCAGAGATTGAGAAACCTATATTTGAGTTAGTTTGG

>>>>>>>

121 ACTGCTCAAACAATTGCTCCAGATAGTGAAGGAGCAATTGATGGGCACCTTCGTGAAGTT

181 GGGCTAACATTTCACCTCCTTAAAGATGTTCCAGGGATCGTCTCAAAGAACATTGACAAG

<<<<<<<<<<<<<<<<<<<<

241 GCATTGGTTGAAGCCTTCAACCCATTAAACATATCTGATTACAACTCTATC

KEYS (in order of precedence):

>>>>>> left primer

<<<<<< right primer

ADDITIONAL OLIGOS

[start](http://bioinfo.ut.ee/primer3-0.4.0/primer3_www_results_help.html#PRIMER_START)  [len](http://bioinfo.ut.ee/primer3-0.4.0/primer3_www_results_help.html#PRIMER_LEN)  [tm](http://bioinfo.ut.ee/primer3-0.4.0/primer3_www_results_help.html#PRIMER_TM)  [gc%](http://bioinfo.ut.ee/primer3-0.4.0/primer3_www_results_help.html#PRIMER_GC)  [any](http://bioinfo.ut.ee/primer3-0.4.0/primer3_www_results_help.html#PRIMER_ANY)  [3'](http://bioinfo.ut.ee/primer3-0.4.0/primer3_www_results_help.html#PRIMER_REPEAT) [seq](http://bioinfo.ut.ee/primer3-0.4.0/primer3_www_results_help.html#PRIMER_OLIGO_SEQ)

1 LEFT PRIMER 48 20 60.16 50.00 8.00 3.00 TGGAGCAGCTGCACTTATTG

RIGHT PRIMER 253 20 59.69 45.00 6.00 3.00 CTTCAACCAATGCCTTGTCA

PRODUCT SIZE: 206, PAIR ANY COMPL: 4.00, PAIR 3' COMPL: 2.00

2 LEFT PRIMER 66 20 59.68 50.00 7.00 2.00 TGTTGGTTCTGATCCAGTGC

RIGHT PRIMER 228 20 60.19 50.00 4.00 3.00 CTTTGAGACGATCCCTGGAA

PRODUCT SIZE: 163, PAIR ANY COMPL: 5.00, PAIR 3' COMPL: 3.00

3 LEFT PRIMER 35 20 59.65 45.00 3.00 1.00 CATTGTTTGGAGATGGAGCA

RIGHT PRIMER 228 20 60.19 50.00 4.00 3.00 CTTTGAGACGATCCCTGGAA

PRODUCT SIZE: 194, PAIR ANY COMPL: 3.00, PAIR 3' COMPL: 0.00

4 LEFT PRIMER 66 20 59.68 50.00 7.00 2.00 TGTTGGTTCTGATCCAGTGC

RIGHT PRIMER 253 20 59.69 45.00 6.00 3.00 CTTCAACCAATGCCTTGTCA

PRODUCT SIZE: 188, PAIR ANY COMPL: 6.00, PAIR 3' COMPL: 3.00

**17 CL5773.Contig2_All**

PAL1，PHE ammonia lyase 1

**表达模式。1h不变，然后降低**

72

| CL5773.Contig2_All | 1561 | 139 | 63 | 19.0543 | 8.698131 | -1.13134 | Down |
| --- | --- | --- | --- | --- | --- | --- | --- |

24

| CL5773.Contig2_All | 1561 | 139 | 49 | 19.0543 | 7.29998 | -1.38415 | Down |
| --- | --- | --- | --- | --- | --- | --- | --- |

1

| CL5773.Contig2_All | 1561 | 139 | 128 | 19.0543 | 19.04471 | -0.00073 | Down |
| --- | --- | --- | --- | --- | --- | --- | --- |

>CL5773.Contig2_All size 1561 gap 0 0%

ATTCCCTCAAAGATTTCCTATTTCTCCTCTCTTTTCCCTCTAGTCTCTACCCCTTCTTCCTTTAAACTTTAGTCTTGTTAGTTCTATATAGTTTTAATTGTAACAAGTTTAAAACTTTATAGCAATAATTCATTATTATTACAATAACATGGAAGCAATCAACCACTCCAATGGTCACCTTGCAACCCAAGATGCTACTTTTTGCTTGAGTAGTACTGCTAATGCTACTAATGGGGTAGTTAATGACCCACTCAACTGGGGTGCAGCTGCTGAGTCACTTAAAGGGAGTCACTTGGAGGAGGTGAAGCGCATGGTGGCGGAGTACCGGAAACCGGTGGTCCGGCTAGGCGGCGAGACACTCACGATCTCTCAGGTGGCTGCCATCGCCAGCCATGACCAAGGGGTTAAGGTGGAGCTGTCAGAGTCAGCCAGGGCTGGCGTTAAGGCTAGCAGTGACTGGGTCATGGATAGCATGAACAATGGCACTGATAGCTACGGTGTCACCACCGGTTTCGGTGCTACCTCCCACAGGAGAACAAAACAGGGCGGTGCCTTGCAGAAAGAGCTCATTAGGTTTTTGAATGCTGGAATATTTGGCAATGGTACGGAGTCCAATTGTACCCTACCACACACAGCAACCAGAGCAGCTATGCTAGTGAGAATTAACACTCTTCTCCAAGGGTACTCAGGCATTAGATTTGAAATCTTAGAAGCAATTACCAAGTTCCTTAACAACAACATTACCCCATGCTTGCCACTTAGGGGTACAATTACAGCTTCTGGTGACCTTGTCCCTCTTTCTTACATTGCTGGTTTGTTAACTGGAAGACATAACTCTAAGGCTGTGGGGCCCTCAGGAAAGATCCTTAATGCCAAGGAAGCTTTTGAATTGGCAGGCATTGGTCATGAGTTCTTTGAATTGCAACCAAAAGAAGGTCTTGCACTTGTGAATGGCACTGCTGTTGGTTCTGGCTTAGCTTCTATTGTTCTTTTTGAGGCAAACATACTGGCTGTGTTGTCTGAAATTATATCAGCAATTTTTGCTGAAGTGATGCAAGGGAAGCCTGAATTTACTGACCATTTGACACATAAGTTGAAGCATCACCCTGGACAAATTGAAGCTGCTGCTATTATGGAACACATTTTGGATGGAAGCTCCTATATGAAAGCAGCTAAGAAGTTGCATGACACAGATCCTCTTCAAAAGCCAAAACAAGATCGATATGCCCTTAGAACTTCACCTCAATGGTTAGGTCCTCTAATTGAAGTGATCAGGTTCTCAACCAAGTCAATAGAGAGAGAGATAAACTCTGTGAATGACAACCCTTTGATTGATGTGTCTAGAAACAAGGCCTTGCATGGTGGTAATTTTCAAGGTACCCCAATTGGAGTCTCTATGGATAATACACGTTTGGCTATTGCATCAATTGGAAAACTCATGTTTGCTCAATTCTCTGAGCTTGTCAATGATTTTTACAATAATGGGTTGCCTTCGAATCTCTCTGCTGCTAGAAACCCCAGTTTGGATTATGGTTTCAAAGGAGCTGAAATTGCCATGGCA

>**[AT2G37040](http://www.arabidopsis.org/servlets/TairObject?type=locus&name=AT2G37040).1** | Symbols: PAL1, ATPAL1 | PHE ammonia lyase 1 |

chr2:15557602-15560237 REVERSE LENGTH=725

Length = 725

Plus Strand HSPs:

Score = 1960 (695.0 bits), Expect = 7.6e-203, P = 7.6e-203

Identities = 387/467 (82%), Positives = 421/467 (90%), Frame = +2

Query: 167 SNGHLATQDATFCLSSTANATNGVVN--DPLNWGAAAESLKGSHLEEVKRMVAEYRKPVV 340

SNG DA C N V+N DPLNWGAAAE +KGSHL+EVKRMVAE+RKPVV

Sbjct: 9 SNG--GGVDAMLC-GGDIKTKNMVINAEDPLNWGAAAEQMKGSHLDEVKRMVAEFRKPVV 65

Query: 341 RLGGETLTISQVAAIASHDQGVKVELSESARAGVKASSDWVMDSMNNGTDSYGVTTGFGA 520

LGGETLTI QVAAI++ VKVELSE+ARAGV ASSDWVM+SMN GTDSYGVTTGFGA

Sbjct: 66 NLGGETLTIGQVAAISTIGNSVKVELSETARAGVNASSDWVMESMNKGTDSYGVTTGFGA 125

Query: 521 TSHRRTKQGGALQKELIRFLNAGIFGNGTESNCTLPHTATRAAMLVRINTLLQGYSGIRF 700

TSHRRTK G ALQKELIRFLNAGIFG+ E++ TLPH+ATRAAMLVRINTLLQG+SGIRF

Sbjct: 126 TSHRRTKNGVALQKELIRFLNAGIFGSTKETSHTLPHSATRAAMLVRINTLLQGFSGIRF 185

Query: 701 EILEAITKFLNNNITPCLPLRGTITASGDLVPLSYIAGLLTGRHNSKAVGPSGKILNAKE 880

EILEAIT FLNNNITP LPLRGTITASGDLVPLSYIAGLLTGR NSKA GP+G+ L A+E

Sbjct: 186 EILEAITSFLNNNITPSLPLRGTITASGDLVPLSYIAGLLTGRPNSKATGPNGEALTAEE 245

Query: 881 AFELAGIGHEFFELQPKEGLALVNGTAVGSGLASIVLFEANILAVLSEIISAIFAEVMQG 1060

AF+LAGI FF+LQPKEGLALVNGTAVGSG+AS+VLFE N+L+VL+EI+SA+FAEVM G

Sbjct: 246 AFKLAGISSGFFDLQPKEGLALVNGTAVGSGMASMVLFETNVLSVLAEILSAVFAEVMSG 305

Query: 1061 KPEFTDHLTHKLKHHPGQIEAAAIMEHILDGSSYMKAAKKLHDTDPLQKPKQDRYALRTS 1240

KPEFTDHLTH+LKHHPGQIEAAAIMEHILDGSSYMK A+KLH+ DPLQKPKQDRYALRTS

Sbjct: 306 KPEFTDHLTHRLKHHPGQIEAAAIMEHILDGSSYMKLAQKLHEMDPLQKPKQDRYALRTS 365

Query: 1241 PQWLGPLIEVIRFSTKSIEREINSVNDNPLIDVSRNKALHGGNFQGTPIGVSMDNTRLAI 1420

PQWLGP IEVIR++TKSIEREINSVNDNPLIDVSRNKA+HGGNFQGTPIGVSMDNTRLAI

Sbjct: 366 PQWLGPQIEVIRYATKSIEREINSVNDNPLIDVSRNKAIHGGNFQGTPIGVSMDNTRLAI 425

Query: 1421 ASIGKLMFAQFSELVNDFYNNGLPSNLSAARNPSLDYGFKGAEIAMA 1561

A+IGKLMFAQFSELVNDFYNNGLPSNL+A+RNPSLDYGFKGAEIAMA

Sbjct: 426 AAIGKLMFAQFSELVNDFYNNGLPSNLTASRNPSLDYGFKGAEIAMA 472

OLIGO [start](http://bioinfo.ut.ee/primer3-0.4.0/primer3_www_results_help.html#PRIMER_START)  [len](http://bioinfo.ut.ee/primer3-0.4.0/primer3_www_results_help.html#PRIMER_LEN)  [tm](http://bioinfo.ut.ee/primer3-0.4.0/primer3_www_results_help.html#PRIMER_TM)  [gc%](http://bioinfo.ut.ee/primer3-0.4.0/primer3_www_results_help.html#PRIMER_GC)  [any](http://bioinfo.ut.ee/primer3-0.4.0/primer3_www_results_help.html#PRIMER_ANY)  [3'](http://bioinfo.ut.ee/primer3-0.4.0/primer3_www_results_help.html#PRIMER_REPEAT) [seq](http://bioinfo.ut.ee/primer3-0.4.0/primer3_www_results_help.html#PRIMER_OLIGO_SEQ)

LEFT PRIMER 56 20 59.83 45.00 6.00 3.00 GCCTTGCATGGTGGTAATTT

RIGHT PRIMER 203 20 59.90 45.00 6.00 2.00 GATTCGAAGGCAACCCATTA

SEQUENCE SIZE: 265

INCLUDED REGION SIZE: 265

PRODUCT SIZE: 148, PAIR ANY COMPL: 5.00, PAIR 3' COMPL: 2.00

1 GAGAGAGATAAACTCTGTGAATGACAACCCTTTGATTGATGTGTCTAGAAACAAGGCCTT

>>>>>

61 GCATGGTGGTAATTTTCAAGGTACCCCAATTGGAGTCTCTATGGATAATACACGTTTGGC

>>>>>>>>>>>>>>>

121 TATTGCATCAATTGGAAAACTCATGTTTGCTCAATTCTCTGAGCTTGTCAATGATTTTTA

181 CAATAATGGGTTGCCTTCGAATCTCTCTGCTGCTAGAAACCCCAGTTTGGATTATGGTTT

<<<<<<<<<<<<<<<<<<<<

241 CAAAGGAGCTGAAATTGCCATGGCA

KEYS (in order of precedence):

>>>>>> left primer

<<<<<< right primer

ADDITIONAL OLIGOS

[start](http://bioinfo.ut.ee/primer3-0.4.0/primer3_www_results_help.html#PRIMER_START)  [len](http://bioinfo.ut.ee/primer3-0.4.0/primer3_www_results_help.html#PRIMER_LEN)  [tm](http://bioinfo.ut.ee/primer3-0.4.0/primer3_www_results_help.html#PRIMER_TM)  [gc%](http://bioinfo.ut.ee/primer3-0.4.0/primer3_www_results_help.html#PRIMER_GC)  [any](http://bioinfo.ut.ee/primer3-0.4.0/primer3_www_results_help.html#PRIMER_ANY)  [3'](http://bioinfo.ut.ee/primer3-0.4.0/primer3_www_results_help.html#PRIMER_REPEAT) [seq](http://bioinfo.ut.ee/primer3-0.4.0/primer3_www_results_help.html#PRIMER_OLIGO_SEQ)

1 LEFT PRIMER 56 20 59.83 45.00 6.00 3.00 GCCTTGCATGGTGGTAATTT

RIGHT PRIMER 227 20 59.88 50.00 4.00 2.00 AACTGGGGTTTCTAGCAGCA

PRODUCT SIZE: 172, PAIR ANY COMPL: 4.00, PAIR 3' COMPL: 1.00

2 LEFT PRIMER 56 20 59.83 45.00 6.00 3.00 GCCTTGCATGGTGGTAATTT

RIGHT PRIMER 199 20 59.82 45.00 4.00 2.00 CGAAGGCAACCCATTATTGT

PRODUCT SIZE: 144, PAIR ANY COMPL: 5.00, PAIR 3' COMPL: 0.00

3 LEFT PRIMER 56 20 59.83 45.00 6.00 3.00 GCCTTGCATGGTGGTAATTT

RIGHT PRIMER 262 20 60.21 45.00 4.00 0.00 CATGGCAATTTCAGCTCCTT

PRODUCT SIZE: 207, PAIR ANY COMPL: 5.00, PAIR 3' COMPL: 3.00

4 LEFT PRIMER 56 20 59.83 45.00 6.00 3.00 GCCTTGCATGGTGGTAATTT

RIGHT PRIMER 213 20 60.24 55.00 6.00 2.00 GCAGCAGAGAGATTCGAAGG

PRODUCT SIZE: 158, PAIR ANY COMPL: 4.00, PAIR 3' COMPL: 0.00

**18 Unigene35035_All**

**abscisic acid receptor PYL4-like**

|  | \| PYL4, PYR1-LIKE 4, RCAR10, REGULATORY COMPONENTS OF ABA RECEPTOR 10 \| \| --- \| |
| --- | --- | --- |

Encodes a member of the PYR (pyrabactin resistance )/PYL(PYR1-like)/RCAR (regulatory components of ABA receptor) family proteins with 14 members. PYR/PYL/RCAR family proteins function as abscisic acid sensors. Mediate ABA-dependent regulation of protein phosphatase 2Cs ABI1 and ABI2

72 Unigene35035_All abscisic acid receptor PYL4-like

| Unigene35035_All | 833 | 34 | 59 | 8.734036 | 15.26495 | 0.805502 | Up |
| --- | --- | --- | --- | --- | --- | --- | --- |

24

| Unigene35035_All | 833 | 34 | 46 | 8.734036 | 12.84226 | 0.556178 | Up |
| --- | --- | --- | --- | --- | --- | --- | --- |

1

| Unigene35035_All | 833 | 34 | 154 | 8.734036 | 42.93812 | 2.297539 | Up |
| --- | --- | --- | --- | --- | --- | --- | --- |

>Unigene35035_All size 833 gap 0 0%

CTCTCTCTCTCTCTCTCTCTCATAAAACCAAGCCATTCCAATTCACATTCAAAACACTCAAAAAAGACATCATCATCATCAAACACAAAATGCCACCAATTCCTTCAAACTCTTCTCTCTTATTCCACAAGATGAACCACAACAACCACCCTCCTCCAGTCCCCGACACCGTGGCTCGCCACCACACCCACGCCGTCTCCCCCAACCAGTGCTGCTCCGCTGTCATCCAAGAGATCACCGCCCCCGTCTCCACCGTATGGTCCGTCGTCCGCCGCTTCGACAACCCTCAGGCATACAAACACTTCGTCAAGAGCTGCCACGTCATCCTCGGCGACGGCAACGTCGGCACCCTCCGTGAAGTACACGTCATCTCCGGCCTCCCCGCCGCCGTCAGCACGGAGCGCCTCGACCTCCTCGACGACGAGAGCCACGTCATCGGCTTCAGCATGGTCGGCGGCGATCACCGCCTTGCCAATTACCGCTCCGTCACAACCCTCCACCCGTCATGCTCCGGCACGGTGGTCGTGGAGTCCTACGTGGTGGACGTGCCGCCGGGAAACACCACCGAGGACACGTGCGTGTTCGTCGACACTATTCTTCGGTGCAATCTTCAATCGCTCGCGAGATTCGCCGAGAATTTGGCGAGAACGATTTCTAGAGCCTTTTGAATTTGAACTCCCAGAAGTTAAGGCGAATGTGACGATGGCTGTGATTGTGATGCAACTTGCGGTATGGGTTGTCTACTTGTCTTGTGTTGGTACCTTCACTTTTTGTCTAGTTTGCGAACGTATAATTATGTATGGATCGATTTGTCGAATGTAGCAGTTTTTTCG

>**[AT2G38310](http://www.arabidopsis.org/servlets/TairObject?type=locus&name=AT2G38310).1** | Symbols: PYL4, RCAR10 | PYR1-like 4 | chr2:16050251-16050874

FORWARD LENGTH=207

Length = 207

Plus Strand HSPs:

Score = 632 (227.5 bits), Expect = 3.3e-62, P = 3.3e-62

Identities = 122/161 (75%), Positives = 134/161 (83%), Frame = +3

Query: 168 TVARHHTHAVSPNQCCSAVIQEITAPVSTVWSVVRRFDNPQAYKHFVKSCHVILGDG-NV 344

T AR HTH V PNQCCSAVIQEI+AP+STVWSVVRRFDNPQAYKHF+KSC VI GDG NV

Sbjct: 38 TAARFHTHEVGPNQCCSAVIQEISAPISTVWSVVRRFDNPQAYKHFLKSCSVIGGDGDNV 97

Query: 345 GTLREVHVISGLPAAVSTERLDLLDDESHVIGFSMVGGDHRLANYRSVTTLHPS-CSGTX 521

G+LR+VHV+SGLPAA STERLD+LDDE HVI FS+VGGDHRL+NYRSVTTLHPS SGT

Sbjct: 98 GSLRQVHVVSGLPAASSTERLDILDDERHVISFSVVGGDHRLSNYRSVTTLHPSPISGTV 157

Query: 522 XXXXXXXXXPPGNTTEDTCVFVDTILRCNLQSLARFAENLA 644

PPGNT E+TC FVD I+RCNLQSLA+ AEN A

Sbjct: 158 VVESYVVDVPPGNTKEETCDFVDVIVRCNLQSLAKIAENTA 198

OLIGO [start](http://bioinfo.ut.ee/primer3-0.4.0/primer3_www_results_help.html#PRIMER_START)  [len](http://bioinfo.ut.ee/primer3-0.4.0/primer3_www_results_help.html#PRIMER_LEN)  [tm](http://bioinfo.ut.ee/primer3-0.4.0/primer3_www_results_help.html#PRIMER_TM)  [gc%](http://bioinfo.ut.ee/primer3-0.4.0/primer3_www_results_help.html#PRIMER_GC)  [any](http://bioinfo.ut.ee/primer3-0.4.0/primer3_www_results_help.html#PRIMER_ANY)  [3'](http://bioinfo.ut.ee/primer3-0.4.0/primer3_www_results_help.html#PRIMER_REPEAT) [seq](http://bioinfo.ut.ee/primer3-0.4.0/primer3_www_results_help.html#PRIMER_OLIGO_SEQ)

LEFT PRIMER 104 20 60.00 50.00 4.00 0.00 AATTACCGCTCCGTCACAAC

RIGHT PRIMER 236 20 59.86 50.00 4.00 2.00 TGCACCGAAGAATAGTGTCG

SEQUENCE SIZE: 274

INCLUDED REGION SIZE: 274

PRODUCT SIZE: 133, PAIR ANY COMPL: 4.00, PAIR 3' COMPL: 3.00

1 CTCCGGCCTCCCCGCCGCCGTCAGCACGGAGCGCCTCGACCTCCTCGACGACGAGAGCCA

61 CGTCATCGGCTTCAGCATGGTCGGCGGCGATCACCGCCTTGCCAATTACCGCTCCGTCAC

>>>>>>>>>>>>>>>>>

121 AACCCTCCACCCGTCATGCTCCGGCACGGTGGTCGTGGAGTCCTACGTGGTGGACGTGCC

>>>

181 GCCGGGAAACACCACCGAGGACACGTGCGTGTTCGTCGACACTATTCTTCGGTGCAATCT

<<<<<<<<<<<<<<<<<<<<

241 TCAATCGCTCGCGAGATTCGCCGAGAATTTGGCG

KEYS (in order of precedence):

>>>>>> left primer

<<<<<< right primer

ADDITIONAL OLIGOS

[start](http://bioinfo.ut.ee/primer3-0.4.0/primer3_www_results_help.html#PRIMER_START)  [len](http://bioinfo.ut.ee/primer3-0.4.0/primer3_www_results_help.html#PRIMER_LEN)  [tm](http://bioinfo.ut.ee/primer3-0.4.0/primer3_www_results_help.html#PRIMER_TM)  [gc%](http://bioinfo.ut.ee/primer3-0.4.0/primer3_www_results_help.html#PRIMER_GC)  [any](http://bioinfo.ut.ee/primer3-0.4.0/primer3_www_results_help.html#PRIMER_ANY)  [3'](http://bioinfo.ut.ee/primer3-0.4.0/primer3_www_results_help.html#PRIMER_REPEAT) [seq](http://bioinfo.ut.ee/primer3-0.4.0/primer3_www_results_help.html#PRIMER_OLIGO_SEQ)

1 LEFT PRIMER 104 20 60.00 50.00 4.00 0.00 AATTACCGCTCCGTCACAAC

RIGHT PRIMER 251 20 61.86 50.00 4.00 2.00 CGAGCGATTGAAGATTGCAC

PRODUCT SIZE: 148, PAIR ANY COMPL: 5.00, PAIR 3' COMPL: 1.00

2 LEFT PRIMER 104 20 60.00 50.00 4.00 0.00 AATTACCGCTCCGTCACAAC

RIGHT PRIMER 237 21 61.20 47.62 4.00 2.00 TTGCACCGAAGAATAGTGTCG

PRODUCT SIZE: 134, PAIR ANY COMPL: 4.00, PAIR 3' COMPL: 3.00

3 LEFT PRIMER 90 20 62.13 50.00 5.00 0.00 ATCACCGCCTTGCCAATTAC

RIGHT PRIMER 236 20 59.86 50.00 4.00 2.00 TGCACCGAAGAATAGTGTCG

PRODUCT SIZE: 147, PAIR ANY COMPL: 3.00, PAIR 3' COMPL: 1.00

4 LEFT PRIMER 105 20 62.22 55.00 3.00 0.00 ATTACCGCTCCGTCACAACC

RIGHT PRIMER 236 20 59.86 50.00 4.00 2.00 TGCACCGAAGAATAGTGTCG

PRODUCT SIZE: 132, PAIR ANY COMPL: 4.00, PAIR 3' COMPL: 2.00

**19 CL5462.Contig1_All isoflavone synthase**

72h

| CL5462.Contig1_All | 2070 | 2661 | 3293 | 275.0778 | 369.8214 | 0.426989 | Up |
| --- | --- | --- | --- | --- | --- | --- | --- |

24h

| CL5462.Contig1_All | 2070 | 2661 | 938 | 275.0778 | 105.3807 | -1.38423 | Down |
| --- | --- | --- | --- | --- | --- | --- | --- |

1h

| CL5462.Contig1_All | 2070 | 2661 | 1877 | 275.0778 | 210.6014 | -0.38532 | Down |
| --- | --- | --- | --- | --- | --- | --- | --- |

>CL5462.Contig1_All size 2070 gap 0 0%

TTGAGAACCAATCAAACAAATTAAAGTAGCACGCCACGAGTTCTTAGACTAGAAGTGGTAGGTGATATTAGATCCATTCGATTTCATTCAACTACTTACTGAAGTAGTATTGCCCATTCTGAAGTAGAATGATGAGTTGCTTATAAAGGGGTAGCGTAGGAAACCTTTCCCTTCAGGCAAGCAAAAAAGCTCTGGTACATAGCCTCAAACTCAGCAGGACTCTTACTTACACCCGTTATTGGAAAAACCATGTTGCTCGAACTTGCAATCACTTTATTGGTGATAGCCCTTTTCATCCACTTGCGTCCCTCACCAACAGCAAAATCGAAGGCCCTTCGCCACCTCCCTAACCCTCCTTCTCCTAAGCCACGACTCCCATTCATAGGGCACCTGCACCTTTTGAACCAACCTCTTATCCACGAATCTCTCATCAAACTCTCCGAGCGCTATGGCCCTTTGTACTCTCTCTACTTTGGTTCCATGCCTACCATCGTTGCCTCCACCCCTGACCTTTTCAAACTCTTCCTCCAAACCCACGAGGTTTCTTCCTTCAACACAAGGTTCCAAACCTCAGCCATCAGGCGCCTCACATATGACAACTCTGTGGCCATGGTTCCCTTTGGACCTTACTGGAAGTTCATCAGGAAGCTCATCATGAATGATCTCCTCAACGCCACCACTGTTAACAAGTTGAGACCCTTGAGGAGCCAAGAAATCCGTAAGGTTCTTAAGGTGCTAGCCAGTAACGCAGATGTTCAACAACCCCTTAATGTCACTGAGGAACTCCTCAAGTGGACTAACAGCACCATCTCTAGGATGATGTTGGGTGAAGCTGAAGAGATTAGAGATATTGCTCGTGAGGTGCTTAAGATCTTTGGGGAATATAGTCTTACTGACTTTATTTGGCCATTGAAGAAGCTCAAGGTTGGAGAATATGAGAAGAGAATTGATAATATCTTTAACAAGTTTGATCCCGTCATTGAAAGGGTTATTAAGAAACGCCAAGAGATAAGGAAGAGGAGAAAGGAGAGAAATGGAGAAATTGAAGAGGGTGAGCAGAGTGTAGTTTTTCTCGATACTTTGCTTGAATTTGCTGAGGACCCCACCATGGAGATCAAGATTACCAAGGAACAAATCAAGGGTCTTGTTGTGGATTTCTTCTCAGCAGGGACAGATTCCACAGCCGTGGCAACAGACTGGGCTTTGTCAGAGCTCATCAACAACCCTGGGGTGCTGCAAAAAGCCAGAGAGGAAATTGATAGTGTTGTGGGAAAAGATAGGCTTGTTGATGAAGCAGATATTCAGAATCTTCCATACATTAGAGCTATAGTGAAGGAAACATTCCGTATGCACCCACCACTACCCGTGGTCAAGAGAAAATGTGTACAAGAGTGTGAGGTTGATGGCTTTGTGATCCCAGAGGGAGCATTGATACTTTTCAATGTTTGGGCTGTAGGAAGAGATCCAAAATACTGGGAAACCCCATCGGAATTTCGTCCTGAGAGGTTCTTGGATAATGCTGGTGACCCCATTGATCTTAGGGGACAACATTTCCAACTCTTGCCATTTGGGTCTGGAAGGAGGATGTGCCCTGGAGTGAATTTGGCCACTGCTGGAATGGCAACATTGCTTTCATCTCTTATCCAGTGCTTTGACTTGCAGCCTGTGGGCCCACAAGGCCAAATATTGAAAGGTAAAGATGCCAAAGTTAGCATGGAAGGGAGGGCTGGTCTCACTGTTCCAAGGGCCCACAATCTCATCTGTGTTCCAGTTGCTAGAGCAAGCATTGCAGCTAAACTCCTTTCCGCTTAAAATGCAACAGCATCGCTGTGCCTTGGCTTATGCTATTGTAGGGTAATAACCATTTCAATAAAGTATCACTCTTTGTCATCGAATCGAAGTCTCAGCACATGGGCTCAAACATGCACACAACAAATGGGCAAATCTGCTGTACTCCCCCATTTTTATATCATAATGTTATTCCATACCTTTTACAGTTTTTTGTAACTTTGTTATTGTACGTGTTCTCAAATTTTAAAAGTAGTATTAATTTGATTTTTATTTACGAAA

isoflavone synthase [Caragana arborescens]

Sequence ID: [gb|AEQ39026.1|](http://www.ncbi.nlm.nih.gov/protein/351001348?report=genbank&log$=protalign&blast_rank=1&RID=ZVYF7UCZ01R)Length: 524Number of Matches: 1

Related Information

Range 1: 1 to 524[GenPept](http://www.ncbi.nlm.nih.gov/protein/351001348?report=genbank&log$=protalign&blast_rank=1&RID=ZVYF7UCZ01R&from=1&to=524)[Graphics](http://www.ncbi.nlm.nih.gov/protein/351001348?report=graph&rid=ZVYF7UCZ01R%5b351001348%5d&tracks=%5bkey:sequence_track,name:Sequence,display_name:Sequence,id:STD1,category:Sequence,annots:Sequence,ShowLabel:true%5d%5bkey:gene_model_track,CDSProductFeats:false%5d%5bkey:alignment_track,name:other%20alignments,annots:NG%20Alignments%7CRefseq%20Alignments%7CGnomon%20Alignments%7CUnnamed,shown:false%5d&v=0:550&appname=ncbiblast&link_loc=fromHSP) Next Match Previous Match

| Alignment statistics for match #1 | | | | | | |
| --- | --- | --- | --- | --- | --- | --- |
| **Score** | **Expect** | **Method** | **Identities** | **Positives** | **Gaps** | **Frame** |
| 907 bits(2343) | 0.0 | Compositional matrix adjust. | 467/524(89%) | 505/524(96%) | 4/524(0%) | +1 |

Query 250 MLLELAITLLVIALFIHLRPSPTAKSKALRHLpnppspkprlpFIGHLHLLNQPLIHESL 429

ML+ELAITLLVIALF+HLRP+P+AKSKALRHLPNPPSPKPRLPFIGHLHLL++PL+H+SL

Sbjct 1 MLVELAITLLVIALFLHLRPTPSAKSKALRHLPNPPSPKPRLPFIGHLHLLDKPLLHQSL 60

Query 430 IKLSERYGPLYSLYFGSMPTIVASTPDLFKLFLQTHEVSSFNTRFQTSAIRRLTYDNSVA 609

I+LSERYGPLYSLYFGSMPT+VASTP+LFKLFLQTHE SSFNTRFQTSAIRRLTYDNSVA

Sbjct 61 IRLSERYGPLYSLYFGSMPTVVASTPELFKLFLQTHEASSFNTRFQTSAIRRLTYDNSVA 120

Query 610 MVPFGPYWKFIRKLIMNDLLNATTVNKLRPLRSQEIRKVLKVLASNADVQQPLNVTEELL 789

MVPFGPYWKFIRKLIMNDLLNATTVNKLRPLRSQEIRKVLKV+A +A+ QQPLNVTEELL

Sbjct 121 MVPFGPYWKFIRKLIMNDLLNATTVNKLRPLRSQEIRKVLKVMAQSAETQQPLNVTEELL 180

Query 790 KWTNSTISRMMLGEAEEIRDIAREVLKIFGEYSLTDFIWPLKKLKVGEYEKRIDNIFNKF 969

KWTNSTISRMMLGEAEEIRDIAR+VLKIFGEYSLTDFIWPLKKLKVG+YEKRID+IFN+F

Sbjct 181 KWTNSTISRMMLGEAEEIRDIARDVLKIFGEYSLTDFIWPLKKLKVGQYEKRIDDIFNRF 240

Query 970 DPViervikkrqeirkrrkerNGEIEEGEQSVVFLDTLLEFAEDPTMEIKITKEQIKGLV 1149

DPVIE+VIKKRQEIRKRRKERNGE+EEGEQSVVFLDTLL+FAED TMEIKITKEQIKGL+

Sbjct 241 DPVIEKVIKKRQEIRKRRKERNGELEEGEQSVVFLDTLLDFAEDETMEIKITKEQIKGLI 300

Query 1150 VDFFSAGTDSTAVATDWALSELINNPGVLQKAREEIDSVVGKDRLVDEADIQNLPYIRAI 1329

VDFFSAGTDSTAVATD+ALSELINNP VLQKAREE+DSVVGKDRLVDE+D+QNLP+IRAI

Sbjct 301 VDFFSAGTDSTAVATDYALSELINNPRVLQKAREEVDSVVGKDRLVDESDVQNLPFIRAI 360

Query 1330 VKETFRMHPPLPVVKRKCVQECEVDGFVIPEGALILFNVWAVGRDPKYWETPSEFRPERF 1509

VKETFRMHPPLPVVKRKC QECE+DGFVIPEGALILFNVWAVGRDPKYWE PSEFRPERF

Sbjct 361 VKETFRMHPPLPVVKRKCTQECEIDGFVIPEGALILFNVWAVGRDPKYWERPSEFRPERF 420

Query 1510 LDNAGD----PIDLRGQHFQLLPFGSGRRMCPGVNLATAGMATLLSSLIQCFDLQPVGPQ 1677

L NAG+ IDLRGQHFQLLPFGSGRRMCPGVNLATAGMATLL+S+IQCFDLQ GPQ

Sbjct 421 LQNAGEGEVGSIDLRGQHFQLLPFGSGRRMCPGVNLATAGMATLLASVIQCFDLQVPGPQ 480

Query 1678 GQILKGKDAKVSMEGRAGLTVPRAHNLICVPVARASIAAKLLSA 1809

G++LKG DAKVSME R GLTVPRA+NL+CVP+ARA +AAKLLS+

Sbjct 481 GELLKGDDAKVSMEERPGLTVPRANNLMCVPLARAGVAAKLLSS 524

OLIGO [start](http://bioinfo.ut.ee/primer3-0.4.0/primer3_www_results_help.html#PRIMER_START)  [len](http://bioinfo.ut.ee/primer3-0.4.0/primer3_www_results_help.html#PRIMER_LEN)  [tm](http://bioinfo.ut.ee/primer3-0.4.0/primer3_www_results_help.html#PRIMER_TM)  [gc%](http://bioinfo.ut.ee/primer3-0.4.0/primer3_www_results_help.html#PRIMER_GC)  [any](http://bioinfo.ut.ee/primer3-0.4.0/primer3_www_results_help.html#PRIMER_ANY)  [3'](http://bioinfo.ut.ee/primer3-0.4.0/primer3_www_results_help.html#PRIMER_REPEAT) [seq](http://bioinfo.ut.ee/primer3-0.4.0/primer3_www_results_help.html#PRIMER_OLIGO_SEQ)

LEFT PRIMER 30 20 59.71 50.00 4.00 0.00 GCCAAAGTTAGCATGGAAGG

RIGHT PRIMER 176 21 59.40 47.62 4.00 1.00 ATAGCATAAGCCAAGGCACAG

SEQUENCE SIZE: 234

INCLUDED REGION SIZE: 234

PRODUCT SIZE: 147, PAIR ANY COMPL: 3.00, PAIR 3' COMPL: 0.00

1 CACAAGGCCAAATATTGAAAGGTAAAGATGCCAAAGTTAGCATGGAAGGGAGGGCTGGTC

>>>>>>>>>>>>>>>>>>>>

61 TCACTGTTCCAAGGGCCCACAATCTCATCTGTGTTCCAGTTGCTAGAGCAAGCATTGCAG

121 CTAAACTCCTTTCCGCTTAAAATGCAACAGCATCGCTGTGCCTTGGCTTATGCTATTGTA

<<<<<<<<<<<<<<<<<<<<<

181 GGGTAATAACCATTTCAATAAAGTATCACTCTTTGTCATCGAATCGAAGTCTCA

KEYS (in order of precedence):

>>>>>> left primer

<<<<<< right primer

ADDITIONAL OLIGOS

[start](http://bioinfo.ut.ee/primer3-0.4.0/primer3_www_results_help.html#PRIMER_START)  [len](http://bioinfo.ut.ee/primer3-0.4.0/primer3_www_results_help.html#PRIMER_LEN)  [tm](http://bioinfo.ut.ee/primer3-0.4.0/primer3_www_results_help.html#PRIMER_TM)  [gc%](http://bioinfo.ut.ee/primer3-0.4.0/primer3_www_results_help.html#PRIMER_GC)  [any](http://bioinfo.ut.ee/primer3-0.4.0/primer3_www_results_help.html#PRIMER_ANY)  [3'](http://bioinfo.ut.ee/primer3-0.4.0/primer3_www_results_help.html#PRIMER_REPEAT) [seq](http://bioinfo.ut.ee/primer3-0.4.0/primer3_www_results_help.html#PRIMER_OLIGO_SEQ)

1 LEFT PRIMER 30 20 59.71 50.00 4.00 0.00 GCCAAAGTTAGCATGGAAGG

RIGHT PRIMER 159 20 61.78 45.00 8.00 0.00 ACAGCGATGCTGTTGCATTT

PRODUCT SIZE: 130, PAIR ANY COMPL: 5.00, PAIR 3' COMPL: 1.00

2 LEFT PRIMER 30 20 59.71 50.00 4.00 0.00 GCCAAAGTTAGCATGGAAGG

RIGHT PRIMER 158 20 62.27 45.00 6.00 0.00 CAGCGATGCTGTTGCATTTT

PRODUCT SIZE: 129, PAIR ANY COMPL: 5.00, PAIR 3' COMPL: 1.00

3 LEFT PRIMER 53 20 61.26 55.00 3.00 2.00 GGCTGGTCTCACTGTTCCAA

RIGHT PRIMER 176 21 59.40 47.62 4.00 1.00 ATAGCATAAGCCAAGGCACAG

PRODUCT SIZE: 124, PAIR ANY COMPL: 5.00, PAIR 3' COMPL: 3.00

4 LEFT PRIMER 30 20 59.71 50.00 4.00 0.00 GCCAAAGTTAGCATGGAAGG

RIGHT PRIMER 177 22 60.63 45.45 4.00 1.00 AATAGCATAAGCCAAGGCACAG

PRODUCT SIZE: 148, PAIR ANY COMPL: 3.00, PAIR 3' COMPL: 0.00

**20 Unigene33150_All**

**cinnamyl alcohol dehydrogenase 9**

[lignin biosynthetic process](http://www.arabidopsis.org/servlets/TairObject?type=keyword&id=6181), [response to cytokinin stimulus](http://www.arabidopsis.org/servlets/TairObject?type=keyword&id=11401)

72

| Unigene33150_All | 1561 | 897 | 600 | 122.9619 | 82.83934 | -0.56982 | Down |
| --- | --- | --- | --- | --- | --- | --- | --- |

24

| Unigene33150_All | 1561 | 897 | 383 | 122.9619 | 57.05903 | -1.10768 | Down |
| --- | --- | --- | --- | --- | --- | --- | --- |

1

| Unigene33150_All | 1561 | 897 | 663 | 122.9619 | 98.64565 | -0.31788 | Down |
| --- | --- | --- | --- | --- | --- | --- | --- |

>Unigene33150_All size 1561 gap 0 0%

TGGGGTACAAAATCTCATAACGCAAAATGTATTAGCCTAATATGCATTTGGAAGCACCGAATCCAATAAAGGGGATAAAGCCCAAAAACGATGCCCAGTAAGTTTGAGATGTATGCCAGGATGCCACAGCCCACATAGCATGATACAGCAAAAAAGGGAAGAAAAAAGTAAATAAATAGCTGACAAAATCACAAAATAAATCGCTTAGTTCTAGTACTTTATTATTAAGATAAAGCAACATAATATATATTCAAGAGTCCAAACTATTGTACCGAAGGAAAGAGAAAACTTTCTCTTCTCTACTAGTCTGGCATCTTACACCAATCTTTATCTATTCCCAATATGCTGAGACATCTAAGGCATCCATCACATGCAAAGAGTTTAATCATGGTGACCTCTATGAAGTTGATAAGGAGTTCGCCACATCAATCACAAAGCGATATTTCACGTCAGATTTGAGAAGCCTTTCCATGGCCGTATTGATTTCATCAATCTTAATCAGCTCAATATCAGCAGTTATGTTGTGCTTCCCGCAAAAATCAAGCATCTCCTGAGTCTCCTTTATCCCTCCGAAGTTGCTCCCCCCTATAAGCTTCCGTCCCATAACTAAGGGAAAGATAGGCAGCTCGAGGGGCTTGTTAGGCAACCCTACAGTGACCAGCTTCCCGTTCAGCTTCAACAAACCAATCAATGCATTCAGAGAATGAACAGCAGAAATTGTGTCTATGATATAGTCCATGGTCCCCATAGCTGCTTTCATTTTTGCAGGGTCTGTGGAAACAAGGAAAGCATCAGCACCAAGTTTATCAATGGCCTCAGATTCCTTGTTTGGAGAGCCACTAATAACAGTAACTTTCAGCCCAAATGCTTTACCAAATTTGATTGCAACATGGCCTAACCCACCAAGCCCTGCCACACCCAAATGTTTACCTGGCTCTGTCATCCCATAATAAATCATTGGGCTATACACAGTAATCCCAGCACACAGTAGCGGAGCACCAGCATCAAGGGGTAAGTTCTCAGGAAATTGGAGTACAAACCGCTGATGAACAACCACAAAATCAGAATAGCCACCATAGGTTCGTGTCCCATTATAAGGAGAGTTATAGGTAAAAACAGGACGAGGACAGTAACTCTCCAAATCCTGCTGGCAACTCTCACATTCCTTACAAGAGTCCACTATCACACCAACTCCAACTTTATCACCCACTTTGAATTTTTTCACATTTTCTCCAGTTTTTGTCACAATACCAACAATTTCATGCCCAGGAACGACCGGGTAAGTTGTGAAACCCCAATCGTTCTTGACTGTATGAAGATCCGAATGGCAAACCCCACAAAATAGGATTTTGAGAGCGACATCATCAGGGCCATTTTCCCTTCTGGAGAAATGGAAGGGAGAAAGAAGGCCAGAAGTGTCTTTAGCAGCCCAGCCAAAAGACTTCACTGGGAGTTCGTTTTCAGGTGATTTGGCCATTCTATTCTATTCTATCTATCTATTCTTCTCTTCTCTTCTTCTTTCGTTCTAATGTACTAATATATGTGATATCAAAGCAGTGGT

ACCACTGCTTTGATATCACATATATTAGTACATTAGAACGAAAGAAGAAGAGAAGAGAAGAATAGATAGATAGAATAGAATAGAATGGCCAAATCACCTGAAAACGAACTCCCAGTGAAGTCTTTTGGCTGGGCTGCTAAAGACACTTCTGGCCTTCTTTCTCCCTTCCATTTCTCCAGAAGGGAAAATGGCCCTGATGATGTCGCTCTCAAAATCCTATTTTGTGGGGTTTGCCATTCGGATCTTCATACAGTCAAGAACGATTGGGGTTTCACAACTTACCCGGTCGTTCCTGGGCATGAAATTGTTGGTATTGTGACAAAAACTGGAGAAAATGTGAAAAAATTCAAAGTGGGTGATAAAGTTGGAGTTGGTGTGATAGTGGACTCTTGTAAGGAATGTGAGAGTTGCCAGCAGGATTTGGAGAGTTACTGTCCTCGTCCTGTTTTTACCTATAACTCTCCTTATAATGGGACACGAACCTATGGTGGCTATTCTGATTTTGTGGTTGTTCATCAGCGGTTTGTACTCCAATTTCCTGAGAACTTACCCCTTGATGCTGGTGCTCCGCTACTGTGTGCTGGGATTACTGTGTATAGCCCAATGATTTATTATGGGATGACAGAGCCAGGTAAACATTTGGGTGTGGCAGGGCTTGGTGGGTTAGGCCATGTTGCAATCAAATTTGGTAAAGCATTTGGGCTGAAAGTTACTGTTATTAGTGGCTCTCCAAACAAGGAATCTGAGGCCATTGATAAACTTGGTGCTGATGCTTTCCTTGTTTCCACAGACCCTGCAAAAATGAAAGCAGCTATGGGGACCATGGACTATATCATAGACACAATTTCTGCTGTTCATTCTCTGAATGCATTGATTGGTTTGTTGAAGCTGAACGGGAAGCTGGTCACTGTAGGGTTGCCTAACAAGCCCCTCGAGCTGCCTATCTTTCCCTTAGTTATGGGACGGAAGCTTATAGGGGGGAGCAACTTCGGAGGGATAAAGGAGACTCAGGAGATGCTTGATTTTTGCGGGAAGCACAACATAACTGCTGATATTGAGCTGATTAAGATTGATGAAATCAATACGGCCATGGAAAGGCTTCTCAAATCTGACGTGAAATATCGCTTTGTGATTGATGTGGCGAACTCCTTATCAACTTCATAGAGGTCACCATGATTAAACTCTTTGCATGTGATGGATGCCTTAGATGTCTCAGCATATTGGGAATAGATAAAGATTGGTGTAAGATGCCAGACTAGTAGAGAAGAGAAAGTTTTCTCTTTCCTTCGGTACAATAGTTTGGACTCTTGAATATATATTATGTTGCTTTATCTTAATAATAAAGTACTAGAACTAAGCGATTTATTTTGTGATTTTGTCAGCTATTTATTTACTTTTTTCTTCCCTTTTTTGCTGTATCATGCTATGTGGGCTGTGGCATCCTGGCATACATCTCAAACTTACTGGGCATCGTTTTTGGGCTTTATCCCCTTTATTGGATTCGGTGCTTCCAAATGCATATTAGGCTAATACATTTTGCGTTATGAGATTTTGTACCCCA

>**[AT4G39330](http://www.arabidopsis.org/servlets/TairObject?type=locus&name=AT4G39330).1** | Symbols: ATCAD9, CAD9 | cinnamyl alcohol dehydrogenase 9 |

chr4:18291268-18292772 FORWARD LENGTH=360

Length = 360

Plus Strand HSPs:

Score = 1325 (471.5 bits), Expect = 1.6e-135, P = 1.6e-135

Identities = 250/358 (69%), Positives = 288/358 (80%), Frame = +1

Query: 85 MAKSPENELPVKSFGWAAKDTSGLLSPFHFSRRENGPDDVALKILFCGVCHSDLHTVKND 264

MAKSPE E P K FGW A+D SG+LSPFHFSRR+NG +DV +KILFCGVCH+DLHT+KND

Sbjct: 1 MAKSPETEHPNKVFGWGARDKSGVLSPFHFSRRDNGENDVTVKILFCGVCHTDLHTIKND 60

Query: 265 WGFTTYPVVPGHEIVGIVTKTGENXXXXXXXXXXXXXXXXDSCKECESCQQDLESYCPRP 444

WG++ YPVVPGHEIVGI TK G+N SC+ CESC QDLE+YCP+

Sbjct: 61 WGYSYYPVVPGHEIVGIATKVGKNVTKFKEGDRVGVGVISGSCQSCESCDQDLENYCPQM 120

Query: 445 VFTYNS-PYNGTRTYGGYSDFVVVHQRFVLQFPENLPLDAGAPLLCAGITVYSPMIYYGM 621

FTYN+ +GT+ YGGYS+ +VV QRFVL+FPENLP D+GAPLLCAGITVYSPM YYGM

Sbjct: 121 SFTYNAIGSDGTKNYGGYSENIVVDQRFVLRFPENLPSDSGAPLLCAGITVYSPMKYYGM 180

Query: 622 TEPGKXXXXXXXXXXXXXXIKFGKAFGLKVTVISGSPNKESEAIDKLGADAFLVSTDPAK 801

TE GK +K GKAFGLKVTVIS S K EAI+ LGAD+FLV+TDP K

Sbjct: 181 TEAGKHLGVAGLGGLGHVAVKIGKAFGLKVTVISSSSTKAEEAINHLGADSFLVTTDPQK 240

Query: 802 MKAAMGTMDYIIDTISAVHSLNALIGLLKLNGKLVTVGLPNKPLELPIFPLVMGRKLIGG 981

MKAA+GTMDYIIDTISAVH+L L+GLLK+NGKL+ +GLP KPLELP+FPLV+GRK++GG

Sbjct: 241 MKAAIGTMDYIIDTISAVHALYPLLGLLKVNGKLIALGLPEKPLELPMFPLVLGRKMVGG 300

Query: 982 SNFGGIKETQEMLDFCGKHNITADIELIKIDEINTAMERLLKSDVKYRFVIDVANSLS 1155

S+ GG+KETQEMLDFC KHNITADIELIK+DEINTAMERL KSDV+YRFVIDVANSLS

Sbjct: 301 SDVGGMKETQEMLDFCAKHNITADIELIKMDEINTAMERLAKSDVRYRFVIDVANSLS 358

OLIGO [start](http://bioinfo.ut.ee/primer3-0.4.0/primer3_www_results_help.html#PRIMER_START)  [len](http://bioinfo.ut.ee/primer3-0.4.0/primer3_www_results_help.html#PRIMER_LEN)  [tm](http://bioinfo.ut.ee/primer3-0.4.0/primer3_www_results_help.html#PRIMER_TM)  [gc%](http://bioinfo.ut.ee/primer3-0.4.0/primer3_www_results_help.html#PRIMER_GC)  [any](http://bioinfo.ut.ee/primer3-0.4.0/primer3_www_results_help.html#PRIMER_ANY)  [3'](http://bioinfo.ut.ee/primer3-0.4.0/primer3_www_results_help.html#PRIMER_REPEAT) [seq](http://bioinfo.ut.ee/primer3-0.4.0/primer3_www_results_help.html#PRIMER_OLIGO_SEQ)

LEFT PRIMER 11 20 60.14 50.00 2.00 1.00 GCGGGAAGCACAACATAACT

RIGHT PRIMER 133 20 60.08 50.00 2.00 0.00 AGGAGTTCGCCACATCAATC

SEQUENCE SIZE: 279

INCLUDED REGION SIZE: 279

PRODUCT SIZE: 123, PAIR ANY COMPL: 4.00, PAIR 3' COMPL: 0.00

1 CTTGATTTTTGCGGGAAGCACAACATAACTGCTGATATTGAGCTGATTAAGATTGATGAA

>>>>>>>>>>>>>>>>>>>>

61 ATCAATACGGCCATGGAAAGGCTTCTCAAATCTGACGTGAAATATCGCTTTGTGATTGAT

<<<<<<<

121 GTGGCGAACTCCTTATCAACTTCATAGAGGTCACCATGATTAAACTCTTTGCATGTGATG

<<<<<<<<<<<<<

181 GATGCCTTAGATGTCTCAGCATATTGGGAATAGATAAAGATTGGTGTAAGATGCCAGACT

241 AGTAGAGAAGAGAAAGTTTTCTCTTTCCTTCGGTACAAT

KEYS (in order of precedence):

>>>>>> left primer

<<<<<< right primer

ADDITIONAL OLIGOS

[start](http://bioinfo.ut.ee/primer3-0.4.0/primer3_www_results_help.html#PRIMER_START)  [len](http://bioinfo.ut.ee/primer3-0.4.0/primer3_www_results_help.html#PRIMER_LEN)  [tm](http://bioinfo.ut.ee/primer3-0.4.0/primer3_www_results_help.html#PRIMER_TM)  [gc%](http://bioinfo.ut.ee/primer3-0.4.0/primer3_www_results_help.html#PRIMER_GC)  [any](http://bioinfo.ut.ee/primer3-0.4.0/primer3_www_results_help.html#PRIMER_ANY)  [3'](http://bioinfo.ut.ee/primer3-0.4.0/primer3_www_results_help.html#PRIMER_REPEAT) [seq](http://bioinfo.ut.ee/primer3-0.4.0/primer3_www_results_help.html#PRIMER_OLIGO_SEQ)

1 LEFT PRIMER 11 20 60.14 50.00 2.00 1.00 GCGGGAAGCACAACATAACT

RIGHT PRIMER 130 20 60.12 45.00 2.00 0.00 AGTTCGCCACATCAATCACA

PRODUCT SIZE: 120, PAIR ANY COMPL: 4.00, PAIR 3' COMPL: 1.00

2 LEFT PRIMER 11 20 60.14 50.00 2.00 1.00 GCGGGAAGCACAACATAACT

RIGHT PRIMER 134 20 59.56 45.00 2.00 2.00 AAGGAGTTCGCCACATCAAT

PRODUCT SIZE: 124, PAIR ANY COMPL: 4.00, PAIR 3' COMPL: 1.00

3 LEFT PRIMER 11 20 60.14 50.00 2.00 1.00 GCGGGAAGCACAACATAACT

RIGHT PRIMER 185 20 60.64 45.00 4.00 0.00 GCATCCATCACATGCAAAGA

PRODUCT SIZE: 175, PAIR ANY COMPL: 4.00, PAIR 3' COMPL: 1.00

4 LEFT PRIMER 61 20 59.78 45.00 6.00 0.00 ATCAATACGGCCATGGAAAG

RIGHT PRIMER 185 20 60.64 45.00 4.00 0.00 GCATCCATCACATGCAAAGA

PRODUCT SIZE: 125, PAIR ANY COMPL: 5.00, PAIR 3' COMPL: 0.00

**21** **CL2423.Contig1_All**

**cinnamyl alcohol dehydrogenase 5**

72h

| CL2423.Contig1_All | 1493 | 424 | 176 | 60.7697 | 27.4046 | -1.14893 | Down |
| --- | --- | --- | --- | --- | --- | --- | --- |

24h

| CL2423.Contig1_All | 1493 | 424 | 183 | 60.7697 | 28.50492 | -1.09214 | Down |
| --- | --- | --- | --- | --- | --- | --- | --- |

1h

| CL2423.Contig1_All | 1493 | 424 | 274 | 60.7697 | 42.62438 | -0.51167 | Down |
| --- | --- | --- | --- | --- | --- | --- | --- |

>CL2423.Contig1_All size 1493 gap 0 0%

CGTTGAGATGACTTTATCGTTCTCGGAATGAATCATTTAATGTTTCGGTTTGGCACCTTTATATATACGCATCCAATCCACTGAGGCACTATAGTATCCAACCAACCAACCCCACTCACTCGTCATTCAACAACACTTTCCCTTCCCATTTGCTGCTTCTGACAAAAACACAACATGGGCAACCTCGAGGCCGAAAGAACCACAGTAGGATGGGCAGCAAGAGACTCTTCTGGGATTCTCTCTCCATACACCTTTACTCTCAGAAACACAGGCCCGGATGATGTGTACATCAAAGTTCACTACTGTGGAATTTGCCATTCTGATCTCCATCAGATTAAAAATGATCTGGGGATGTCCAACTATCCCATGGTTCCTGGGCATGAAGTGGTTGGCGAGGTACTGGAGGTGGGTTCAAATGTTACCAGGTTCAAAGTGGGAGAGACGGTAGGAGCTGGACTCCTCGTTGGCTGCTGCAAAAGCTGCCACGCATGCCAATCAGATATTGAGCAATACTGCAACAAGAAAATCTGGTCTTACAATGATGTTTATACAGATGGAAAACCCACTCAGGGTGGCTTTGCTGAAACCACCGTCGTGGAGCAGAAGTTTGTTGTGAAAATACCGGAGGGTTTGGCGCCAGAGGAAGTTGCACCGTTGTTGTGTGCTGGTGTGACTGTGTACAGTCCACTGTCACACTTTGGGCTGAAAGAGAGTGGGCTGAGGGGTGCAATACTGGGGCTTGGAGGAGTGGGACACATGGGTGTGAAGATAGCAAAGGCACTGGGTCACCATGTCACTGTGATAAGCTCTTCTGATAAGAAGAAGCAGGAGGCTCTTGAGCACCTTGGAGCTGATGAATATCTGGTTAGCTCAGACGCCACACGCATGCAAGAAACTGCTGATTCACTTGATTATATCATTGACACTGTGCCTGTTGGTCACCCTCTTGAGCCTTATCTTTCCTTGCTCAAGGTTGATGGCAAGCTGATCTTGATGGGTGTCATAAACACTCCTCTTCAATTTGTTAGCCCCATGGTCATGCTAGGGAGGAAGTCAATTACAGGAAGCTTTGTTGGGAGCATGAAGGAGACTGAGGAGATGCTAGAGTTCTGGAAAGAGAAGGGTCTGAATTCCATGATTGAAGTGGTAAAGATGGATTACATTAACAAAGCCTTCGAAAGGTTGGAGAAGAACGATGTGAGATATAGGTTCGTTGTGGATGTTAAAGGCAGCAAACTTGACCAGTGAAAGTATCCAAACAGGACACACACCACACCAATATTATGAAAAGTCTGGACAAATTAATTCCCAACTAAGTTGTTTTGTTTTATTGAGGAGTGTTCTGTTTTCTATGCTTTGATTTCGCTCACTTTGTTGATCAAATTTGTGATACCAAATTTGCACTGAAAGCAAGAATATTTATATACCAAACTTCATACTGTATTAGATTGCCATTTCAGTGTTTAAAGTGTGGATTTTCTTACCCCAAAAAG

>**[AT4G34230](http://www.arabidopsis.org/servlets/TairObject?type=locus&name=AT4G34230).1** | Symbols: CAD5, ATCAD5, CAD-5 | cinnamyl alcohol dehydrogenase 5

| chr4:16386898-16388666 REVERSE LENGTH=357

Length = 357

Plus Strand HSPs:

Score = 1405 (499.6 bits), Expect = 5.1e-144, P = 5.1e-144

Identities = 263/356 (73%), Positives = 300/356 (84%), Frame = +1

Query: 175 MGNLEAERTTVGWAARDSSGILSPYTFTLRNTGPDDVYIKVHYCGICHSDLHQIKNDLGM 354

MG +EAER T GWAARD SGILSPYT+TLR TGP+DV I++ CGICH+DLHQ KNDLGM

Sbjct: 1 MGIMEAERKTTGWAARDPSGILSPYTYTLRETGPEDVNIRIICCGICHTDLHQTKNDLGM 60

Query: 355 SNYPMXXXXXXXXXXXXXXXXXTRFKVGETVGAGLLVGCCKSCHACQSDIEQYCNKKIWS 534

SNYPM ++F VG+ VG G LVGCC C C+ D+EQYC KKIWS

Sbjct: 61 SNYPMVPGHEVVGEVVEVGSDVSKFTVGDIVGVGCLVGCCGGCSPCERDLEQYCPKKIWS 120

Query: 535 YNDVYTDGKPTQGGFAETTVVEQKFVVKIPEGLAPEEVAPLLCAGVTVYSPLSHFGLKES 714

YNDVY +G+PTQGGFA+ TVV QKFVVKIPEG+A E+ APLLCAGVTVYSPLSHFGLK+

Sbjct: 121 YNDVYINGQPTQGGFAKATVVHQKFVVKIPEGMAVEQAAPLLCAGVTVYSPLSHFGLKQP 180

Query: 715 GLRGAILGLGGVGHMGVKIAKALGHHVTVISSSDKKKQEALEHLGADEYLVSSDATRMQE 894

GLRG ILGLGGVGHMGVKIAKA+GHHVTVISSS+KK++EAL+ LGAD+Y++ SD +M E

Sbjct: 181 GLRGGILGLGGVGHMGVKIAKAMGHHVTVISSSNKKREEALQDLGADDYVIGSDQAKMSE 240

Query: 895 TADSLDYIIDTVPVGHPLEPYLSLLKVDGKLILMGVINTPLQFVSPMVMLGRKSITGSFV 1074

ADSLDY+IDTVPV H LEPYLSLLK+DGKLILMGVIN PLQF++P++MLGRK ITGSF+

Sbjct: 241 LADSLDYVIDTVPVHHALEPYLSLLKLDGKLILMGVINNPLQFLTPLLMLGRKVITGSFI 300

Query: 1075 GSMKETEEMLEFWKEKGLNSMIEVVKMDYINKAFERLEKNDVRYRFVVDVKGSKLD 1242

GSMKETEEMLEF KEKGL+S+IEVVKMDY+N AFERLEKNDVRYRFVVDV+GS LD

Sbjct: 301 GSMKETEEMLEFCKEKGLSSIIEVVKMDYVNTAFERLEKNDVRYRFVVDVEGSNLD 356

OLIGO [start](http://bioinfo.ut.ee/primer3-0.4.0/primer3_www_results_help.html#PRIMER_START)  [len](http://bioinfo.ut.ee/primer3-0.4.0/primer3_www_results_help.html#PRIMER_LEN)  [tm](http://bioinfo.ut.ee/primer3-0.4.0/primer3_www_results_help.html#PRIMER_TM)  [gc%](http://bioinfo.ut.ee/primer3-0.4.0/primer3_www_results_help.html#PRIMER_GC)  [any](http://bioinfo.ut.ee/primer3-0.4.0/primer3_www_results_help.html#PRIMER_ANY)  [3'](http://bioinfo.ut.ee/primer3-0.4.0/primer3_www_results_help.html#PRIMER_REPEAT) [seq](http://bioinfo.ut.ee/primer3-0.4.0/primer3_www_results_help.html#PRIMER_OLIGO_SEQ)

LEFT PRIMER 2 21 59.55 52.38 3.00 2.00 TGAAGGAGACTGAGGAGATGC

RIGHT PRIMER 194 20 59.89 55.00 2.00 0.00 GTGGTGTGTGTCCTGTTTGG

SEQUENCE SIZE: 324

INCLUDED REGION SIZE: 324

PRODUCT SIZE: 193, PAIR ANY COMPL: 5.00, PAIR 3' COMPL: 1.00

1 ATGAAGGAGACTGAGGAGATGCTAGAGTTCTGGAAAGAGAAGGGTCTGAATTCCATGATT

>>>>>>>>>>>>>>>>>>>>>

61 GAAGTGGTAAAGATGGATTACATTAACAAAGCCTTCGAAAGGTTGGAGAAGAACGATGTG

121 AGATATAGGTTCGTTGTGGATGTTAAAGGCAGCAAACTTGACCAGTGAAAGTATCCAAAC

<<<<<<

181 AGGACACACACCACACCAATATTATGAAAAGTCTGGACAAATTAATTCCCAACTAAGTTG

<<<<<<<<<<<<<<

241 TTTTGTTTTATTGAGGAGTGTTCTGTTTTCTATGCTTTGATTTCGCTCACTTTGTTGATC

301 AAATTTGTGATACCAAATTTGCAC

KEYS (in order of precedence):

>>>>>> left primer

<<<<<< right primer

ADDITIONAL OLIGOS

[start](http://bioinfo.ut.ee/primer3-0.4.0/primer3_www_results_help.html#PRIMER_START)  [len](http://bioinfo.ut.ee/primer3-0.4.0/primer3_www_results_help.html#PRIMER_LEN)  [tm](http://bioinfo.ut.ee/primer3-0.4.0/primer3_www_results_help.html#PRIMER_TM)  [gc%](http://bioinfo.ut.ee/primer3-0.4.0/primer3_www_results_help.html#PRIMER_GC)  [any](http://bioinfo.ut.ee/primer3-0.4.0/primer3_www_results_help.html#PRIMER_ANY)  [3'](http://bioinfo.ut.ee/primer3-0.4.0/primer3_www_results_help.html#PRIMER_REPEAT) [seq](http://bioinfo.ut.ee/primer3-0.4.0/primer3_www_results_help.html#PRIMER_OLIGO_SEQ)

1 LEFT PRIMER 2 21 59.55 52.38 3.00 2.00 TGAAGGAGACTGAGGAGATGC

RIGHT PRIMER 154 20 60.26 45.00 4.00 2.00 TGCTGCCTTTAACATCCACA

PRODUCT SIZE: 153, PAIR ANY COMPL: 4.00, PAIR 3' COMPL: 1.00

2 LEFT PRIMER 2 21 59.55 52.38 3.00 2.00 TGAAGGAGACTGAGGAGATGC

RIGHT PRIMER 151 20 59.58 45.00 4.00 2.00 TGCCTTTAACATCCACAACG

PRODUCT SIZE: 150, PAIR ANY COMPL: 4.00, PAIR 3' COMPL: 1.00

3 LEFT PRIMER 2 21 59.55 52.38 3.00 2.00 TGAAGGAGACTGAGGAGATGC

RIGHT PRIMER 197 20 59.31 55.00 2.00 0.00 GGTGTGGTGTGTGTCCTGTT

PRODUCT SIZE: 196, PAIR ANY COMPL: 5.00, PAIR 3' COMPL: 0.00

4 LEFT PRIMER 2 21 59.55 52.38 3.00 2.00 TGAAGGAGACTGAGGAGATGC

RIGHT PRIMER 164 20 60.81 45.00 4.00 0.00 TGGTCAAGTTTGCTGCCTTT

PRODUCT SIZE: 163, PAIR ANY COMPL: 4.00, PAIR 3' COMPL: 2.00

**CAD**

_All

Unigene31532_All

| Unigene31282_All | 825 | 1-173 | CAD8, CADB2 |  |
| --- | --- | --- | --- | --- |
| CL2423.Contig1_All | 1493 | 全序 | CAD5 |  |
| CL2423.Contig2_All | 327 | 113-196 | CAD4 |  |
| Unigene17299_All | 614 | 缺Nd端163-362 | CAD7 |  |
| Unigene2580_All | 1897 | 全序 | CAD1 |  |
| Unigene33150_All | 1561 | 全序 | CAD9 | T4G39330 |

Unigene31282 1-173缺少3’端

>Unigene31282_All size 825 gap 0 0%

GGGCTGTACACTGTGATCCCAGCACAAAGGAGAGGAGCAGCAGCATCAAG

AGGTAAGCCATCAGGAATGTTGATCACAAAGTGCTCATCCGCAACCATTG

AGTCAGAGTAGCCTCCATATGTGGTGGTGCCATCAGAATACTTGGCACCA

TATGTGAGAATCAATTTTGGACAATAATTCTCAAGATTATCAGCACAGCT

TTGGCATGAGCGGCACGAACCAACCAAGCATCCCACGCCCACCTTGTCCC

CAATTTTGAACTTTTCTACCTTGCTTCCCACCTCTGTCACTACACCAACT

ATCTCATGCCCAGGTACGAGTGGATAGGTGGAAAAGCCCCATTCGTTCTT

CACCATGTGGAGGTCCGAGTGACATATCCCACAGTACAACACTTTGAATG

CCACATCCTTCTCACCCGTTTCCCTTCTGGAGAACTTGAAAGGGGAGAGA

ACACCAGAAGGATCCCTCGCTGCCCATCCGAAAGCCTTCCTAGGATGCTC

AAGTTCAGGCTGTGTTGCCATTTGGATCCGTTGTATTGTATTGAAGAGAA

TAAGTGTACAACAACTCAGAAAGGATTTGTGAGAGTATGGAACATATGAG

GATGGTGGAGCTTAAAGAGCTATTTATAATAAGTGGCAGTGAGGGTTACA

CGTGACGTCCTTGCAAACGTTGTTGACGTCTTTCGTGACGAAAGTGCCTT

CACTTCAGTCTTCAGTTCACACATGGCTTGCCTTTTGGTCCCGAAATCCA

ACACCCACCCATAAGGGTGAAATTGAAGGGGTATGGTATGGTATGGTATG

GTTTGGTATGGGTGGTCGGTCAGTA

Mannitol dehydrogenase [Medicago truncatula]

Sequence ID: [ref|XP_003612979.1|](http://www.ncbi.nlm.nih.gov/protein/357485383?report=genbank&log$=protalign&blast_rank=1&RID=ZXTY94XH014)Length: 262Number of Matches: 1

Range 1: 1 to 173[GenPept](http://www.ncbi.nlm.nih.gov/protein/357485383?report=genbank&log$=protalign&blast_rank=1&RID=ZXTY94XH014&from=1&to=173)[Graphics](http://www.ncbi.nlm.nih.gov/protein/357485383?report=graph&rid=ZXTY94XH014%5b357485383%5d&tracks=%5bkey:sequence_track,name:Sequence,display_name:Sequence,id:STD1,category:Sequence,annots:Sequence,ShowLabel:true%5d%5bkey:gene_model_track,CDSProductFeats:false%5d%5bkey:alignment_track,name:other%20alignments,annots:NG%20Alignments%7CRefseq%20Alignments%7CGnomon%20Alignments%7CUnnamed,shown:false%5d&v=0:181&appname=ncbiblast&link_loc=fromHSP) Next Match Previous Match

| Alignment statistics for match #1 | | | | | | |
| --- | --- | --- | --- | --- | --- | --- |
| **Score** | **Expect** | **Method** | **Identities** | **Positives** | **Gaps** | **Frame** |
| 287 bits(734) | 5e-93 | Compositional matrix adjust. | 149/173(86%) | 160/173(92%) | 0/173(0%) | -2 |

Query 521 MATQPELEHPRKAFGWAARDPSGVLSPFKFSRRETGEKDVAFKVLYCGICHSDLHMVKNE 342

MATQPE EHP KAFGWAARD SGVLSPF FSRRETGEKDVAFKVLYCGICHSDLHM+KNE

Sbjct 1 MATQPEFEHPNKAFGWAARDTSGVLSPFNFSRRETGEKDVAFKVLYCGICHSDLHMIKNE 60

Query 341 WGFSTYPLVPGHEIVGVVTEVGSKVEKFKIGDKVGVGCLVGSCRSCQSCADNLENYCPKL 162

WG STYPLVPGHEI G+VTEVGSKVEKFKIGDKVGVGCLV SCR+CQ+C +NLENYCPK

Sbjct 61 WGMSTYPLVPGHEIAGIVTEVGSKVEKFKIGDKVGVGCLVDSCRACQNCEENLENYCPKQ 120

Query 161 ILTYGAKYSDGTTTYGGYSDSMVADEHFVINIpdglpldaaapllcaGITVYS 3

TY AKYSDG+ TYGGYSDSMVADEHF+++IPDGLPL++AAPLLCAGITVYS

Sbjct 121 TNTYSAKYSDGSITYGGYSDSMVADEHFIVHIPDGLPLESAAPLLCAGITVYS 173

Unigene2580_All: CAD1

| 72 | Unigene2580_All | 1897 | 279 | 260 | 31.47154 | 29.5389 | -0.09143 | Down |
| --- | --- | --- | --- | --- | --- | --- | --- | --- |
| 24 | Unigene2580_All | 1897 | 279 | 228 | 31.47154 | 27.95092 | -0.17115 | Down |
| 1 | Unigene2580_All | 1897 | 279 | 187 | 31.47154 | 22.89505 | -0.45901 | Down |

>**[AT1G72680](http://www.arabidopsis.org/servlets/TairObject?type=locus&name=AT1G72680).1** | Symbols: ATCAD1, CAD1 | cinnamyl-alcohol dehydrogenase |

chr1:27359346-27360876 REVERSE LENGTH=355

Length = 355

Minus Strand HSPs:

Score = 1204 (428.9 bits), Expect = 9.2e-142, Sum P(2) = 9.2e-142

Identities = 222/287 (77%), Positives = 258/287 (89%), Frame = -2

Query: 1287 HEIAGIVTKVGSNVHRFNVGDHVGVGTYVNSCRDCEYCNDRLEVNCIKGSVFTFNGVDVD 1108

HEIAGIVTKVG NV RF VGDHVGVGTYVNSCR+CEYCN+ EVNC KG VFTFNG+D D

Sbjct: 70 HEIAGIVTKVGPNVQRFKVGDHVGVGTYVNSCRECEYCNEGQEVNCAKG-VFTFNGIDHD 128

Query: 1107 GTITKGGYSSYIVVHERYCFMIPKSYPLASAAPLLCAGITVYSPMIRHKMNQPGKSLGVI 928

G++TKGGYSS+IVVHERYC+ IP YPL SAAPLLCAGITVY+PM+RH MNQPGKSLGVI

Sbjct: 129 GSVTKGGYSSHIVVHERYCYKIPVDYPLESAAPLLCAGITVYAPMMRHNMNQPGKSLGVI 188

Query: 927 GLGGLGHMAVKFGKAFGLNVTIFSSSISKKEEALSVLGADKFVVSSDQEQMTALAKSLDF 748

GLGGLGHMAVKFGKAFGL+VT+FS+SISKKEEAL++LGA+ FV+SSD +QM AL KSLDF

Sbjct: 189 GLGGLGHMAVKFGKAFGLSVTVFSTSISKKEEALNLLGAENFVISSDHDQMKALEKSLDF 248

Query: 747 IIDTASGDHPFDPYMSLLKTCGVLVLVGFPSEVKFSPASLNIGMKTVSGSLTGGTKDTQE 568

++DTASGDH FDPYMSLLK G VLVGFPSE+K SPA+LN+GM+ ++GS+TGGTK TQ+

Sbjct: 249 LVDTASGDHAFDPYMSLLKIAGTYVLVGFPSEIKISPANLNLGMRMLAGSVTGGTKITQQ 308

Query: 567 MIDFCAANGIYPNIEVIPMEYANEALERVINRDVKYRFVIDIENSLR 427

M+DFCAA+ IYPNIEVIP++ NEALERV+ +D+KYRFVIDI+NSL+

Sbjct: 309 MLDFCAAHKIYPNIEVIPIQKINEALERVVKKDIKYRFVIDIKNSLK 355

Score = 201 (75.8 bits), Expect = 9.2e-142, Sum P(2) = 9.2e-142

Identities = 36/67 (53%), Positives = 46/67 (68%), Frame = -2

Query: 1563 SSEGVTEDCLGWAAGDASGILSPYKFSRRTLGNGDVYIKITHCGVCYADIIWTRNQHATQ 1384

SSE V +C+ WAA D SG+LSP+ +RR++ DV + ITHCGVCYAD+IW+RNQH

Sbjct: 3 SSESVENECMCWAARDPSGLLSPHTITRRSVTTDDVSLTITHCGVCYADVIWSRNQHGDS 62

Query: 1383 SILLCLG 1363

L G

Sbjct: 63 KYPLVPG 69

PREDICTED: probable cinnamyl alcohol dehydrogenase 1-like isoform X1 [Glycine max]

Sequence ID: [ref|XP_003545737.1|](http://www.ncbi.nlm.nih.gov/protein/356554814?report=genbank&log$=protalign&blast_rank=1&RID=ZXTGEA66015)Length: 357Number of Matches: 1

| **Score** | **Expect** | **Method** | **Identities** | **Positives** | **Gaps** | **Frame** |
| --- | --- | --- | --- | --- | --- | --- |
| 606 bits(1562) | 0.0 | Compositional matrix adjust. | 308/380(81%) | 331/380(87%) | 25/380(6%) | -2 |

Query 1566 MSSEGVTEDCLGWAAGDASGILSPYKFSRRTLGNGDVYIKITHCGVCYADIIWTRNQHAT 1387

MSS+GV EDCLGWAA DASG+LSPYKFSRRT GN DV IKITHCGVC+AD++WTRN+H

Sbjct 1 MSSKGVGEDCLGWAARDASGVLSPYKFSRRTPGNEDVLIKITHCGVCFADVVWTRNKHGD 60

Query 1386 QSILLCLGSGFILLYGWESIYLS*YFCGSLHIRHEIAGIVTKVGSNVHRFNVGDHVGVGT 1207

+ G HEIAGIVTKVGSNVHRF VGDHVGVGT

Sbjct 61 SKYPVVPG-------------------------HEIAGIVTKVGSNVHRFKVGDHVGVGT 95

Query 1206 YVNSCRDCEYCNDRLEVNCIKGSVFTFNGVDVDGTITKGGYSSYIVVHERYCFMIPKSYP 1027

YVNSCRDCE+CNDR EV+C KGSVFTFNGVD DGTITKGGYSSYIVVHERYCF IPKSY

Sbjct 96 YVNSCRDCEHCNDREEVHCTKGSVFTFNGVDFDGTITKGGYSSYIVVHERYCFTIPKSYA 155

Query 1026 LASAAPLLCAGITVYSPMIRHKMNQPGKSLGVIGLGGLGHMAVKFGKAFGLNVTIFSSSI 847

LASAAPLLCAGITVYSPM+RHKMNQPGKSLGVIGLGGLGHMAVKFGKAFGL+VT+FS+SI

Sbjct 156 LASAAPLLCAGITVYSPMVRHKMNQPGKSLGVIGLGGLGHMAVKFGKAFGLSVTVFSTSI 215

Query 846 SKKEEALSVLGADKFVVSSDQEQMTALAKSLDFIIDTASGDHPFDPYMSLLKTCGVLVLV 667

SKKEEALS+LGADKFVVSS+QE+MTALAKSLDFIIDTASGDHPFDPYMSLLKT GV VLV

Sbjct 216 SKKEEALSLLGADKFVVSSNQEEMTALAKSLDFIIDTASGDHPFDPYMSLLKTYGVFVLV 275

Query 666 GFPSEVKFSPASLNIGMKTVSGSLTGGTKDTQEMIDFCAANGIYPNIEVIPMEYANEALE 487

GFPS+VKFSPASLNIG KTV+GS+TGGTKD QEMIDFCAAN I+PNIEVIP+EYANEALE

Sbjct 276 GFPSQVKFSPASLNIGSKTVAGSVTGGTKDIQEMIDFCAANEIHPNIEVIPIEYANEALE 335

Query 486 RVINRDVKYRFVIDIENSLR 427

R+INRDVKYRFVIDIENSL+

Sbjct 336 RLINRDVKYRFVIDIENSLK 355

**22 CL1268.Contig2_All** RD22

| CL1268.Contig2_All | 1537 | 16580 | 7601 | 2308.297 | 1065.823 | -1.11486 | Down |
| --- | --- | --- | --- | --- | --- | --- | --- |
| CL1268.Contig2_All | 1537 | 16580 | 4204 | 2308.297 | 636.0882 | -1.85953 | Down |
| CL1268.Contig2_All | 1537 | 16580 | 7402 | 2308.297 | 1118.517 | -1.04524 | Down |

>CL1268.Contig2_All size 1537 gap 0 0%

GGCTTAATAAGTTTATCTTATAATTATAGGAAAAACTAGGTGAAGCAAATAACTTTATCTTATTACATGTCATATAAAATATATGATAAAAATGAGTTATGTAGTATAAATTTATTCTATCATGCTTGATATAGTGTAGCAAAGAGCCATAACATAGGGAAACAAGGAAGCCACACTATATCATACAGTATTCGTATTCAACACACATATAATGCACCATTGGAAAATATACATAGGAAGCAAAGCAATCGTCTAAACACTGCAAATATTCGGATTGCGTTCCGTAGCTGATCAATGCTACTGAAACTGGTTATTCAAGGAAGCTAGAGAAAAAGAGTTTTCACGTAAATCTACTTGGGAACCCAAACAACGTGATCCTCAGGAAGGAAGTGGCAAACCGGAACGGTTCCTGGCTTAACTTTGAGCACTTGAAAGGCCAAATGCTTAGGGTTCCATTCTGATGTGTCTGTGTGGCAAACTGCTACAGCTTTAACTCTAATCCCATCCGCACCTTCCAAAGGCACAGAATAAGCTCTAGTAGTACTCCGTTTTGTGACAGTAAAAGACAAACATAAGGGTAATTCTGTTTGTGACACACGACAGCCTTGTCCCCTGATATCTTCGTTACACCAGGTGTTATGGTGTATTTTTGCAACTGTGTTTCTTTGTCTACTTCAGTGGATACGGCCTCAACCTTTTTCCCTAGCTTGGAAGTTGTAAAATCAACCATGGATTCTAGTGAAGTTGCACAATACTTTTCCTCACCTCTAATGCCAGAATCTTCACATTCGTTAATAGTTTTCTTCATAACCTCAGCCTCCTCTGACCCAGGTTTGACAGAAAACACATTAAATATATCTTCCACCTTGTTTGATGAAAAAGGTATCGAGTCTGCAGCTTGGCGAGGCAAGAATGTGGCATCATTTGAGGTCTTAGTGAAATGCAAGTCCAATTTTGTTGCAGGGCGCAAGTCTTTTTCCAAGAAGAAGAGTGCTACATTAGGGTCATCATGTAATTGGGTCTCTCTGGCAGCATAGTTGTAAGCAAATGGACTACGTCCAGCTCCAACTCCAACATAGACAGGCTTTCCCTTTTTGCCTCCTGCGTGTACATTTACGCCACCTTTCCCCACGTTCACTGCGGTGCCTCCAGGCTTTCCTTTTGCTGCATGCACATTTACGCCTCCTTTCCCAACATTCACGGTGGTGCCTCCGGGCTTTCCTTTCCCTGCATCAACATTTACACCACCCTTCCCTACACCCACGTTGGTGCTTTTGTCTTCCACCAAATCAGGGTGAAGGGTATCACTGATGGCTTTTGGCATTGGCGTAGTGGGAAGCACGGACTTCCAGTAAACTTCAGGAGGTAAGGCGGCATGGGTTGCCACCATTACAAAATTGATTAAAGCAAATATGGGTAGGAGAGGATACTCCATATATAGGGAAGCAAAGCTGCAACCAAATGCTTGGTGGTGGTAGAGGTAGAGAATTAGAGGAACCAAAAGCGAGGCTGGAATGGAATGATGATCTCAAAGTAG

CTACTTTGAGATCATCATTCCATTCCAGCCTCGCTTTTGGTTCCTCTAATTCTCTACCTCTACCACCACCAAGCATTTGGTTGCAGCTTTGCTTCCCTATATATGGAGTATCCTCTCCTACCCATATTTGCTTTAATCAATTTTGTAATGGTGGCAACCCATGCCGCCTTACCTCCTGAAGTTTACTGGAAGTCCGTGCTTCCCACTACGCCAATGCCAAAAGCCATCAGTGATACCCTTCACCCTGATTTGGTGGAAGACAAAAGCACCAACGTGGGTGTAGGGAAGGGTGGTGTAAATGTTGATGCAGGGAAAGGAAAGCCCGGAGGCACCACCGTGAATGTTGGGAAAGGAGGCGTAAATGTGCATGCAGCAAAAGGAAAGCCTGGAGGCACCGCAGTGAACGTGGGGAAAGGTGGCGTAAATGTACACGCAGGAGGCAAAAAGGGAAAGCCTGTCTATGTTGGAGTTGGAGCTGGACGTAGTCCATTTGCTTACAACTATGCTGCCAGAGAGACCCAATTACATGATGACCCTAATGTAGCACTCTTCTTCTTGGAAAAAGACTTGCGCCCTGCAACAAAATTGGACTTGCATTTCACTAAGACCTCAAATGATGCCACATTCTTGCCTCGCCAAGCTGCAGACTCGATACCTTTTTCATCAAACAAGGTGGAAGATATATTTAATGTGTTTTCTGTCAAACCTGGGTCAGAGGAGGCTGAGGTTATGAAGAAAACTATTAACGAATGTGAAGATTCTGGCATTAGAGGTGAGGAAAAGTATTGTGCAACTTCACTAGAATCCATGGTTGATTTTACAACTTCCAAGCTAGGGAAAAAGGTTGAGGCCGTATCCACTGAAGTAGACAAAGAAACACAGTTGCAAAAATACACCATAACACCTGGTGTAACGAAGATATCAGGGGACAAGGCTGTCGTGTGTCACAAACAGAATTACCCTTATGTTTGTCTTTTACTGTCACAAAACGGAGTACTACTAGAGCTTATTCTGTGCCTTTGGAAGGTGCGGATGGGATTAGAGTTAAAGCTGTAGCAGTTTGCCACACAGACACATCAGAATGGAACCCTAAGCATTTGGCCTTTCAAGTGCTCAAAGTTAAGCCAGGAACCGTTCCGGTTTGCCACTTCCTTCCTGAGGATCACGTTGTTTGGGTTCCCAAGTAGATTTACGTGAAAACTCTTTTTCTCTAGCTTCCTTGAATAACCAGTTTCAGTAGCATTGATCAGCTACGGAACGCAATCCGAATATTTGCAGTGTTTAGACGATTGCTTTGCTTCCTATGTATATTTTCCAATGGTGCATTATATGTGTGTTGAATACGAATACTGTATGATATAGTGTGGCTTCCTTGTTTCCCTATGTTATGGCTCTTTGCTACACTATATCAAGCATGATAGAATAAATTTATACTACATAACTCATTTTTATCATATATTTTATATGACATGTAATAAGATAAAGTTATTTGCTTCACCTAGTTTTTCCTATAATTATAAGATAAACTTATTAAGCC

PREDICTED: dehydration-responsive protein RD22-like [Glycine max]

Sequence ID: [ref|XP_003522684.1|](http://www.ncbi.nlm.nih.gov/protein/356507867?report=genbank&log$=protalign&blast_rank=1&RID=ZXZR2PNA014)Length: 341Number of Matches: 3

| **Score** | **Expect** | **Method** | **Identities** | **Positives** | **Gaps** | **Frame** |
| --- | --- | --- | --- | --- | --- | --- |
| 337 bits(865) | 3e-123 | Compositional matrix adjust. | 198/289(69%) | 217/289(75%) | 21/289(7%) | +1 |

Query 103 MEYPLLPIFALINFVMVATHAALPPEVYWKSVLPTTPMPKAISDTLHPDLVEDKSTNvgv 282

MEY LLPIF L+N +VATHAALPPEVYWKSVLPTTPMPKAI+D L+ D VE+KS++V V

Sbjct 1 MEYRLLPIFTLLNIALVATHAALPPEVYWKSVLPTTPMPKAITDILYSDWVEEKSSSVHV 60

Query 283 gkggvnvdagkgkpggttvnvgkggvnvhaakgkPGGTAVNVGKGGVNVHAggkkgkpvy 462

G GGVNV GKG GTTVNVG G G V+V G

Sbjct 61 GGGGVNVHTGKGGGSGTTVNVGGKGGGGVNVHAGHKGKPVHVSVGS-------------- 106

Query 463 vgvgagRSPFAYNYAARETQLHDDPNVALFFLEKDLRPATKLDLHFTK-TSNDATFLPRQ 639

+SPF Y YAA ETQLHDDPNVALFFLEKDL TKLDLHFT+ TSN ATFL RQ

Sbjct 107 ------KSPFDYVYAATETQLHDDPNVALFFLEKDLHSGTKLDLHFTRSTSNQATFLSRQ 160

Query 640 AADSIPFSSNKVEDIFNVFSVKPGSEEAEVMKKTINECEDSGIRGEEKYCATSLESMVDF 819

ADSIPFSSNKV+ IFN FSVKPGSEEA++MK TI+ECE+ GI+GEEKYCATSLESMVDF

Sbjct 161 VADSIPFSSNKVDFIFNKFSVKPGSEEAQIMKNTISECEEGGIKGEEKYCATSLESMVDF 220

Query 820 TTSKLGKKVEAVSTEVDKETQLQKYTITPGVTKISGDKAVVCHKQNYPY 966

+TSKLG VE VSTEVDKET LQKYT+ PGV K+SGDKAVVCHKQNYPY

Sbjct 221 STSKLGNNVEVVSTEVDKETGLQKYTVAPGVKKLSGDKAVVCHKQNYPY 269

Range 2: 272 to 341[GenPept](http://www.ncbi.nlm.nih.gov/protein/356507867?report=genbank&log$=protalign&blast_rank=1&RID=ZXZR2PNA014&from=272&to=341)[Graphics](http://www.ncbi.nlm.nih.gov/protein/356507867?report=graph&rid=ZXZR2PNA014%5b356507867%5d&tracks=%5bkey:sequence_track,name:Sequence,display_name:Sequence,id:STD1,category:Sequence,annots:Sequence,ShowLabel:true%5d%5bkey:gene_model_track,CDSProductFeats:false%5d%5bkey:alignment_track,name:other%20alignments,annots:NG%20Alignments%7CRefseq%20Alignments%7CGnomon%20Alignments%7CUnnamed,shown:false%5d&v=269:344&appname=ncbiblast&link_loc=fromHSP) Next Match Previous Match [First Match](http://blast.ncbi.nlm.nih.gov/Blast.cgi#hsp356507867_1)

| Alignment statistics for match #2 | | | | | | |
| --- | --- | --- | --- | --- | --- | --- |
| **Score** | **Expect** | **Method** | **Identities** | **Positives** | **Gaps** | **Frame** |
| 133 bits(335) | 3e-123 | Compositional matrix adjust. | 61/70(87%) | 64/70(91%) | 0/70(0%) | +3 |

Query 975 FTVTKRSTTRAYSVPLEGADGIRVKAVAVCHTDTSEWNPKHLAFQVLKVKPGTVPVCHFL 1154

F K TTRAYSVPLEG +G+RVKAVAVCHTDTSEWNPKHLAFQVLKVKPGT+PVCHFL

Sbjct 272 FYCHKTETTRAYSVPLEGTNGVRVKAVAVCHTDTSEWNPKHLAFQVLKVKPGTIPVCHFL 331

Query 1155 PEDHVVWVPK 1184

PEDHVVWVPK

Sbjct 332 PEDHVVWVPK 341

Range 3: 271 to 278[GenPept](http://www.ncbi.nlm.nih.gov/protein/356507867?report=genbank&log$=protalign&blast_rank=1&RID=ZXZR2PNA014&from=271&to=278)[Graphics](http://www.ncbi.nlm.nih.gov/protein/356507867?report=graph&rid=ZXZR2PNA014%5b356507867%5d&tracks=%5bkey:sequence_track,name:Sequence,display_name:Sequence,id:STD1,category:Sequence,annots:Sequence,ShowLabel:true%5d%5bkey:gene_model_track,CDSProductFeats:false%5d%5bkey:alignment_track,name:other%20alignments,annots:NG%20Alignments%7CRefseq%20Alignments%7CGnomon%20Alignments%7CUnnamed,shown:false%5d&v=271:278&appname=ncbiblast&link_loc=fromHSP) Next Match Previous Match [First Match](http://blast.ncbi.nlm.nih.gov/Blast.cgi#hsp356507867_1)

| Alignment statistics for match #3 | | | | | | |
| --- | --- | --- | --- | --- | --- | --- |
| **Score** | **Expect** | **Method** | **Identities** | **Positives** | **Gaps** | **Frame** |
| 21.9 bits(45) | 3e-123 | Compositional matrix adjust. | 8/8(100%) | 8/8(100%) | 0/8(0%) | +2 |

Query 971 VFYCHKTE 994

VFYCHKTE

Sbjct 271 VFYCHKTE 278

OLIGO [start](http://bioinfo.ut.ee/primer3-0.4.0/primer3_www_results_help.html#PRIMER_START)  [len](http://bioinfo.ut.ee/primer3-0.4.0/primer3_www_results_help.html#PRIMER_LEN)  [tm](http://bioinfo.ut.ee/primer3-0.4.0/primer3_www_results_help.html#PRIMER_TM)  [gc%](http://bioinfo.ut.ee/primer3-0.4.0/primer3_www_results_help.html#PRIMER_GC)  [any](http://bioinfo.ut.ee/primer3-0.4.0/primer3_www_results_help.html#PRIMER_ANY)  [3'](http://bioinfo.ut.ee/primer3-0.4.0/primer3_www_results_help.html#PRIMER_REPEAT) [seq](http://bioinfo.ut.ee/primer3-0.4.0/primer3_www_results_help.html#PRIMER_OLIGO_SEQ)

LEFT PRIMER 40 20 60.09 55.00 4.00 1.00 GTCAAACCTGGGTCAGAGGA

RIGHT PRIMER 198 20 60.32 50.00 4.00 0.00 GGATACGGCCTCAACCTTTT

SEQUENCE SIZE: 300

INCLUDED REGION SIZE: 300

PRODUCT SIZE: 159, PAIR ANY COMPL: 4.00, PAIR 3' COMPL: 1.00

1 TCATCAAACAAGGTGGAAGATATATTTAATGTGTTTTCTGTCAAACCTGGGTCAGAGGAG

>>>>>>>>>>>>>>>>>>>>

61 GCTGAGGTTATGAAGAAAACTATTAACGAATGTGAAGATTCTGGCATTAGAGGTGAGGAA

121 AAGTATTGTGCAACTTCACTAGAATCCATGGTTGATTTTACAACTTCCAAGCTAGGGAAA

<<

181 AAGGTTGAGGCCGTATCCACTGAAGTAGACAAAGAAACACAGTTGCAAAAATACACCATA

<<<<<<<<<<<<<<<<<<

241 ACACCTGGTGTAACGAAGATATCAGGGGACAAGGCTGTCGTGTGTCACAAACAGAATTAC

KEYS (in order of precedence):

>>>>>> left primer

<<<<<< right primer

ADDITIONAL OLIGOS

[start](http://bioinfo.ut.ee/primer3-0.4.0/primer3_www_results_help.html#PRIMER_START)  [len](http://bioinfo.ut.ee/primer3-0.4.0/primer3_www_results_help.html#PRIMER_LEN)  [tm](http://bioinfo.ut.ee/primer3-0.4.0/primer3_www_results_help.html#PRIMER_TM)  [gc%](http://bioinfo.ut.ee/primer3-0.4.0/primer3_www_results_help.html#PRIMER_GC)  [any](http://bioinfo.ut.ee/primer3-0.4.0/primer3_www_results_help.html#PRIMER_ANY)  [3'](http://bioinfo.ut.ee/primer3-0.4.0/primer3_www_results_help.html#PRIMER_REPEAT) [seq](http://bioinfo.ut.ee/primer3-0.4.0/primer3_www_results_help.html#PRIMER_OLIGO_SEQ)

1 LEFT PRIMER 40 20 60.09 55.00 4.00 1.00 GTCAAACCTGGGTCAGAGGA

RIGHT PRIMER 192 20 60.42 50.00 4.00 2.00 GGCCTCAACCTTTTTCCCTA

PRODUCT SIZE: 153, PAIR ANY COMPL: 4.00, PAIR 3' COMPL: 2.00

2 LEFT PRIMER 41 20 60.23 55.00 4.00 0.00 TCAAACCTGGGTCAGAGGAG

RIGHT PRIMER 198 20 60.32 50.00 4.00 0.00 GGATACGGCCTCAACCTTTT

PRODUCT SIZE: 158, PAIR ANY COMPL: 4.00, PAIR 3' COMPL: 1.00

3 LEFT PRIMER 41 20 60.23 55.00 4.00 0.00 TCAAACCTGGGTCAGAGGAG

RIGHT PRIMER 192 20 60.42 50.00 4.00 2.00 GGCCTCAACCTTTTTCCCTA

PRODUCT SIZE: 152, PAIR ANY COMPL: 4.00, PAIR 3' COMPL: 1.00

4 LEFT PRIMER 40 20 60.09 55.00 4.00 1.00 GTCAAACCTGGGTCAGAGGA

RIGHT PRIMER 201 20 60.90 55.00 4.00 2.00 AGTGGATACGGCCTCAACCT

PRODUCT SIZE: 162, PAIR ANY COMPL: 4.00, PAIR 3' COMPL: 2.00
